# Design, synthesis, and biological evaluation of new arylamide derivatives possessing sulfonate or sulfamate moieties as steroid sulfatase enzyme inhibitors

**DOI:** 10.1016/j.bmc.2016.04.040

**Published:** 2016-06-15

**Authors:** Mohammed I. El-Gamal, Mohammad H. Semreen, Paul A. Foster, Barry V.L. Potter

**Affiliations:** a Department of Medicinal Chemistry, College of Pharmacy, University of Sharjah, Sharjah 27272, United Arab Emirates; b Sharjah Institute for Medical Research, University of Sharjah, Sharjah 27272, United Arab Emirates; c Department of Medicinal Chemistry, Faculty of Pharmacy, University of Mansoura, Mansoura 35516, Egypt; d Institute of Metabolism and Systems Research, University of Birmingham, Birmingham B15 2TT, United Kingdom; e Centre for Endocrinology, Diabetes and Metabolism, Birmingham Health Partners, Birmingham B15 2HT, United Kingdom; f Department of Pharmacology, University of Oxford, Mansfield Road, Oxford OX1 3QT, United Kingdom

**Keywords:** Arylamide, JEG-3, Steroid sulfatase, Sulfamate, Sulfonate

## Abstract

A series of new arylamide derivatives possessing terminal sulfonate or sulfamate moieties was designed and synthesized. The target compounds were tested for in vitro inhibitory effects against the steroid sulfatase (STS) enzyme in a cell-free assay system. The free sulfamate derivative **1j** was the most active. It inhibited the enzymatic activity by 72.0% and 55.7% at 20 μM and 10 μM, respectively. Compound **1j** was further tested for STS inhibition in JEG-3 placental carcinoma cells with high STS enzyme activity. It inhibited 93.9% of the enzyme activity in JEG-3 placental carcinoma cells at 20 μM with an efficacy near to that of the well-established drug STX64 as reference. At 10 μM, **1j** inhibited 86.1% of the STS activity of JEG-3. Its IC_50_ value against the STS enzyme in JEG-3 cells was 0.421 μM. Thus, **1j** represents an attractive new non-steroidal lead for further optimization.

## Graphical abstract

**Figure:**
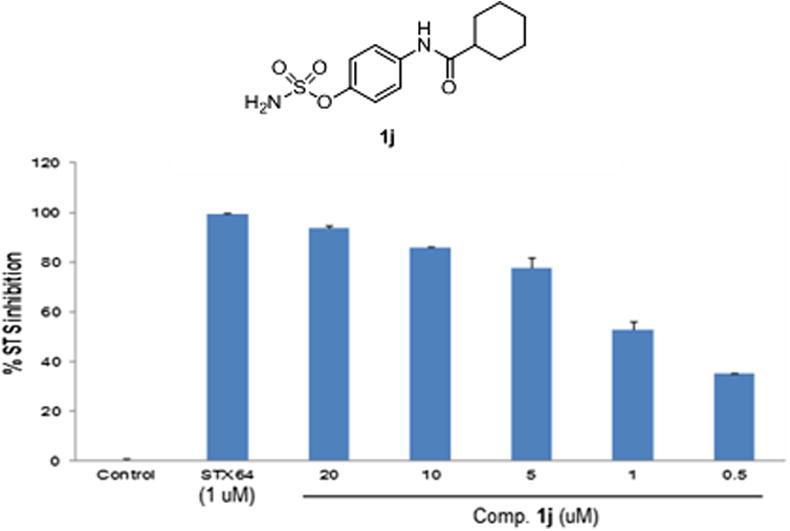

## 1 Introduction

The steroid sulfatase (STS) enzyme catalyzes the hydrolysis of inactive sulfate metabolites such as estrone sulfate and dehydroepiandrosterone sulfate to the more active estrone and dehydroepiandrosterone, respectively. The production of 90% of androstenediol (Adiol) comes from dehydroepiandrosterone released through the STS pathway.^1^ Despite the androgenic structure of Adiol, it still possesses some estrogenic properties. Adiol is about 100 times weaker than estradiol,^2^, ^3^, ^4^, ^5^ with lower affinity for the estrogen receptor.^6^ However, the Adiol concentration in the circulation is 100-fold higher than estradiol. This led to speculation that it might be equipotent to estradiol.^7^ In addition, the STS pathway produces a significant amount of estrogen besides that produced by aromatase, the enzyme which catalyzes the aromatization of androgen to estrogen. This has been supported by: (1) STS activity in liver, normal breast tissues, and breast cancer tissues is million fold higher than aromatase activity;^8^ (2) estrone produced from estrone sulfate through the STS pathway is about 10-fold higher than that produced from androstenedione through aromatase action;^9^ and (3) STS expression is a very essential prognostic factor in human breast carcinoma.^10^, ^11^ Thus, STS is an attractive target for the treatment of hormone-dependent breast,^12^ endometrial,^13^ prostate cancers, and endometriosis.^14^

**Figure 1:**
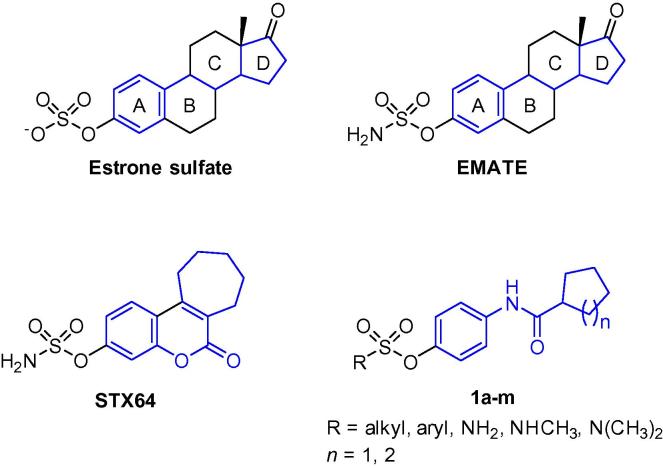
Structures of Estrone sulfate, EMATE, STX64, and the target compounds **1a**–**m**.

Several articles have recently highlighted different steroidal and non-steroidal agents capable of inhibiting STS.^12^, ^15^, ^16^, ^17^, ^18^, ^19^, ^20^, ^21^ Estrone 3-*O*-sulfamate (EMATE, Fig. 1) is an example of a potent steroidal STS inhibitors, but when orally tested in vivo it exerted estrogenic side effects as demonstrated by its ability to increase the uterine weight in ovariectomized Wistar rats.^22^ Attention was therefore switched to non-steroidal STS inhibitors to avoid such effects. The coumarin sulfamate derivative STX64 (Irosustat, 667 COUMATE, Fig. 1) has been the most potent and successful STS inhibitor to date. It is currently being investigated in clinical trials for treatment of estrogen-dependent breast cancer, and has been trialed in endometrial cancer and prostate cancer. STX64 is an irreversible STS inhibitor due to the presence of the sulfamate moiety that covalently binds to the enzyme.^16^ On the other hand, some estrone sulfonate derivatives have been reported as reversible STS inhibitors because the sulfonate moiety is unable to make a covalent bond with the enzyme as the sulfamate analogues.^23^, ^24^

It is attractive to explore novel non-steroidal templates as potential sulfatase inhibitors. In the present study, a series of arylamide derivatives possessing sulfonate or sulfamate moieties was designed to mimic estrone sulfate and dehydroepiandrosterone sulfate, the substrates of STS. As illustrated in Figure 1, the two ring system of the target compounds mimic rings A and D of estrone sulfate and EMATE with a 2-atom spacer. In another orientation, it can also mimic the aromatic and the cycloheptane rings of STX64 with an amide linker as an isostere of the coumarin ester moiety. Thirteen target compounds were synthesized and evaluated for STS inhibitory effect in a cell-free enzymatic assay. The most promising compound was further tested for its STS inhibitory effect in whole JEG-3 placental carcinoma cells that have high STS enzyme activity. The results and experimental protocols are set out below.

## 2 Results and discussion

### 2.1 Chemistry

**Scheme 1:**
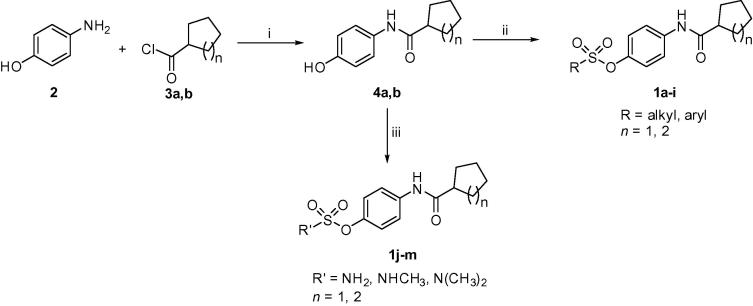
Reagents and conditions: (i) anhydrous K_2_CO_3_, acetone, 0 °C, rt, 4 h; (ii) appropriate sulfonyl chloride derivative, triethylamine, anhydrous THF, 0 °C, rt, 2 h, 80–88% (two steps); (iii) appropriate sulfamoyl chloride derivative, NaH, anhydrous DMF, 0 °C, rt, overnight, 83–90% (two steps).

The target compounds **1a**–**m** were synthesized via the pathway illustrated in Scheme 1. 4-Aminophenol (**2**) was reacted with cyclohexanecarbonyl chloride (**3a**) or cyclopentanecarbonyl chloride (**3b**) in the presence of anhydrous potassium carbonate to afford the phenolic intermediates **4a**,**b**. Some precautions were taken into consideration in this reaction to avoid disubstitution, such as the order of addition, rate of addition, dilution with solvent, and stirring while adding the acid chlorides to 4-aminophenol. Interaction of the hydroxyl intermediates **4a**,**b** with the appropriate sulfonyl chloride derivatives in the presence of triethylamine produced the target sulfonate compounds **1a**–**i**. To obtain the target sulfamate analogues **1j**–**m**, compounds **4a**,**b** were reacted with the appropriate sulfamoyl chloride reagents in presence of anhydrous sodium hydride under N_2_. The detailed structures of the target compounds are illustrated in Table 1.

**Table 1:** Structures of the target compounds **1a**–**m**

**Figure:**
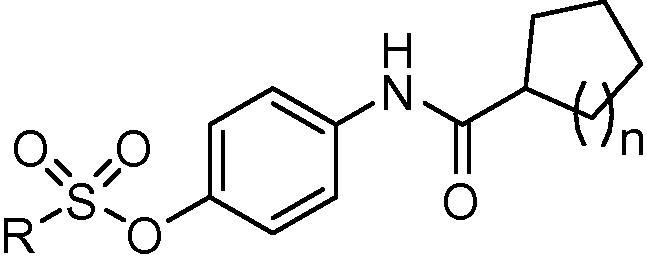

Compound No.	R	*n*
**1a**	Me	2
**1b**	Et	2
**1c**	*n*-Pr	2
**1d**	Ph	2
**1e**	4-Me(C_6_H_4_)	2
**1f**	4-*tert*-Butyl(C_6_H_4_)	2
**1g**	4-F(C_6_H_4_)	2
**1h**	4-CF_3_(C_6_H_4_)	2
**1i**	4-Me(C_6_H_4_)	1
**1j**	NH_2_	2
**1k**	NHMe	2
**1l**	N(Me)_2_	2
**1m**	NHMe	1

### 2.2 Biological screening

#### 2.2.1 Cell-free enzyme inhibition testing

**Figure 2:**
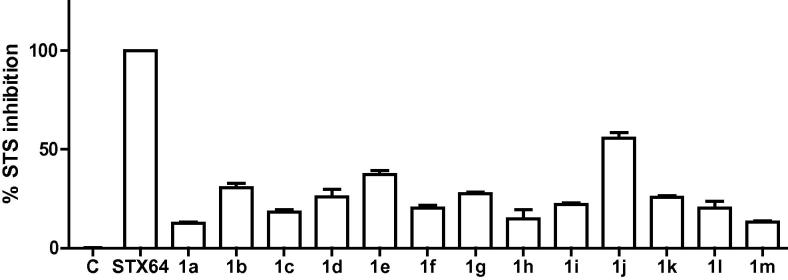
Inhibition percentages expressed by compounds **1a**–**m** at 10 μM concentration against steroid sulfatase enzyme.

All the thirteen target compounds **1a**–**m** were tested at a single-dose concentration of 10 μM against the STS enzyme. The inhibitory effects are depicted in Figure 2. The results show that compound **1j** is the most active amongst this series of compounds. It possesses a free sulfamate ‘warhead’ moiety, similar to the lead compound STX64. Irosustat (STX64) has been reported as an irreversible inhibitor of STS. The irreversible inhibitors are usually stronger than the corresponding reversible inhibitory agents. This explains the stronger activity of the free sulfamate analogue **1j** that likely irreversibly also inhibits the enzyme similar to STX64, compared to the sulfonate derivatives that were less active.

The free sulfamate compound **1j** was significantly more active than the *N*-substituted sulfamate derivatives **1k** and **1l**. This finding complies with earlier data reported for STX64 and its steroidal counterparts compared with the corresponding substituted sulfamate analogues.^16^ The substituted sulfamate moieties have been reported as reversible inhibitors and non-covalent binders relative to the free sulfamate.^25^ This can rationalize the stronger activity of free sulfamate derivatives compared to the substituted sulfamates.

Among the aliphatic sulfonate analogues, the ethanesulfonate **1b** was the most active, and the *p*-tosylate derivative **1e** was more active than the other aromatic sulfonates. Upon investigating the effect of the cycloalkyl ring size on activity, the cyclohexyl derivatives **1e** and **1k** were more active than the corresponding cyclopentyl analogues **1i** and **1m**. So the bulkier cyclohexyl ring is more optimal for activity maybe due to stronger hydrophobic interactions and/or steric influence. Any or both of these effects might enhance the affinity to the enzyme and hence confer a stronger inhibitory effect.

**Figure 3:**
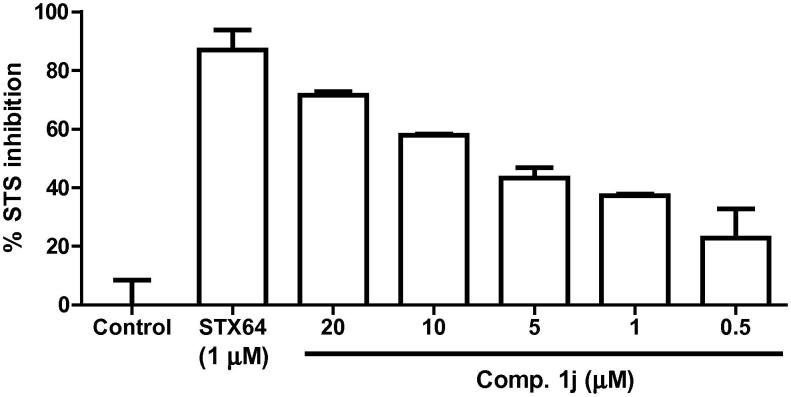
% inhibition exerted by STX64 and compound **1j** at different concentrations against cell-free STS enzyme. The results are expressed as mean of triplicate assay ± SEM.

The most promising compound **1j** was further studied in 5-dose testing mode at 20, 10, 5, 1, and 0.5 μM concentration in comparison with STX64. The results are illustrated in Figure 3. Compound **1j** inhibited the enzyme in a dose-dependent manner. It inhibited 72.0% of the enzymatic activity at 20 μM concentration. This compound could be a useful template for future lead optimization to design new STS inhibitors.

#### 2.2.2 Cell-based enzyme inhibition testing

**Figure 4:**
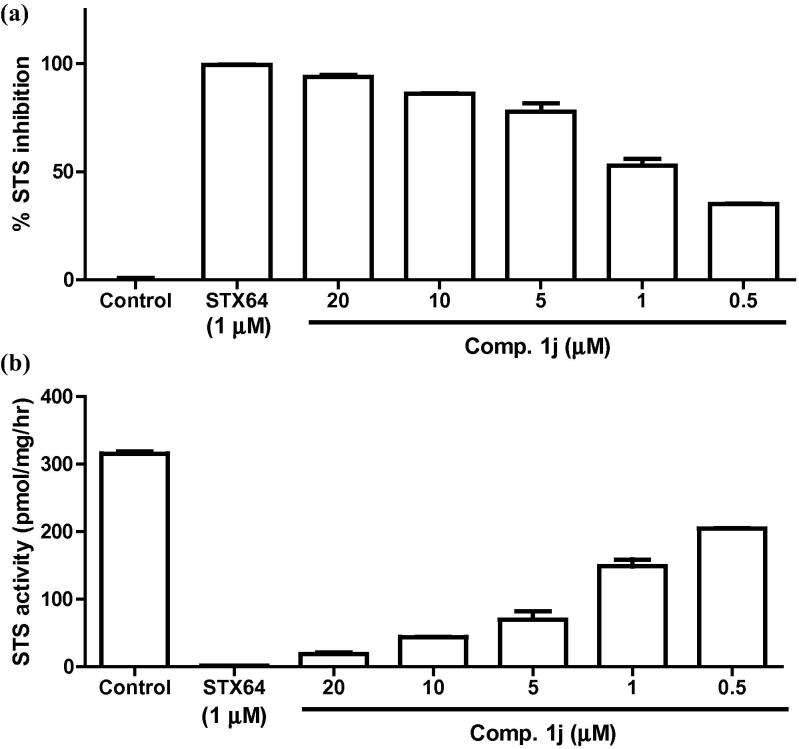
(a) % inhibition exerted by STX64 and compound **1j** at different concentrations against STS enzyme in JEG-3 placental carcinoma cells. (b) Residual STS enzyme activity after treatment with STX64 and compound **1j**. The results are expressed as mean of a triplicate assay ± SEM.

Compound **1j** was further tested for STS enzyme inhibition in a JEG-3 placental carcinoma cell line with over-expressed STS. It was tested at five different concentrations starting from 20 μM, and compared with STX64 (1 μM) as a reference standard. Compound **1j** could clearly penetrate the cell membrane to enter the cells, so it is hydrophobic enough to this end. It inhibited the JEG-3 STS in a dose-dependent manner as illustrated in Figure 4. The inhibition at 20 μM was 93.9%, very close to the result for STX64 result; the enzyme activity was inhibited by 86.1% at a 10 μM concentration of **1j**.

**Figure 5:**
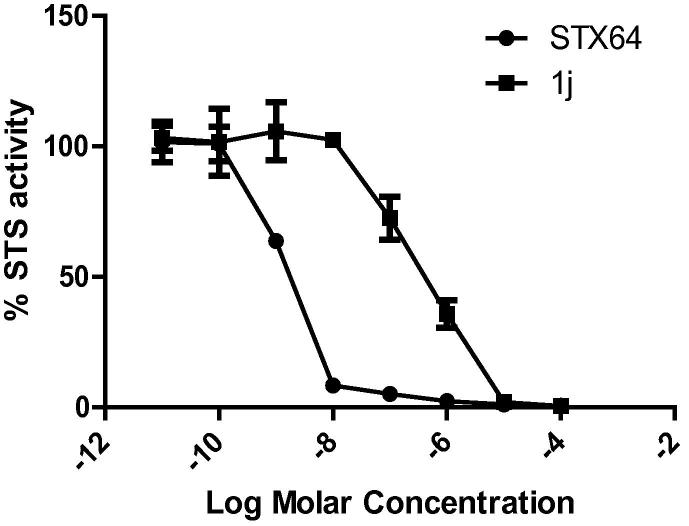
% STS enzyme activity in JEG-3 cells upon treatment with different concentrations of STX64 and compound **1j**.

Compounds **1j** and STX64 were further tested in an 8-dose testing mode in order to study the dose-STS response (Fig. 5) and calculate the IC_50_ values of both compounds. The IC_50_ values of STX64 and compound **1j** against STS activity were 1.7 nM and 0.421 μM, respectively.

## 3 Conclusion

In the present study, a new series of sulfonate- or sulfamate-containing arylamide compounds was designed and synthesized. All the target compounds were characterized by ^1^H NMR, ^13^C NMR, IR, and LC–MS analyses. They were tested for STS inhibitory effects against JEG-3 lysate. The free sulfamate compound **1j** was the most active among this series of compounds; it inhibited the enzyme in a dose-dependent manner and it was much more active than the sulfonate or *N*-substituted sulfamate analogues. The cyclohexyl motif was found more favorable for activity than cyclopentyl. Compound **1j** could penetrate the cell membrane of JEG-3 placental carcinoma cells rich in STS activity to inhibit the enzyme inside the cells in a dose-dependent manner. It showed very high and promising STS inhibitory effects against JEG-3 cells, albeit weaker than Irosustat. Thus, this compound provides a promising hit for future lead optimization in order to develop perhaps still more potent and more promising STS enzyme inhibitors. Further decoration of the two ring systems to this end should be straightforward to accomplish.

## 4 Experimental

### 4.1 General

The target compounds were purified by column chromatography using silica gel (0.040–0.063 mm, 230–400 mesh) and technical grade solvents. IR spectra (KBr discs) were recorded with a Bruker FT-IR instrument (Bruker Bioscience, Billerica, MA, USA). ^1^H NMR and ^13^C NMR spectra were recorded on a Bruker Avance 400 spectrometer using tetramethylsilane as an internal standard. LC–MS analyses were carried out in positive ion mode by Electrospray Ionization (ESI) on (Waters) ACQUITY UPLC triple Quadrupole (Xevo TQD) instrument equipped with MassLynx software. The samples were dissolved in methanol diluted in spray solution (methanol/water 1:1 ^v^/_v_ 0.1% formic acid) and infused directly in combined mode with a flow rate of 0.3 mL/min. Melting points were obtained on a Walden Precision Apparatus Electrothermal 9300 apparatus and are uncorrected. Solvents and liquid reagents were transferred using hypodermic syringes. All solvents and reagents were commercially available and used without further purification.

### 4.2 Synthesis of *N*-(4-hydroxyphenyl)cyclohexanecarboxamide (**4a**) and *N*-(4-hydroxyphenyl)cyclopentanecarboxamide (**4b**)

To a solution of 4-aminophenol (100 mg, 0.916 mmol) in acetone (15 mL), anhydrous K_2_CO_3_ (152 mg, 1.1 mmol) was added. The reaction mixture was stirred at room temperature for 15 min, then cooled to 0 °C. A solution of cyclohexanecarbonyl chloride or cyclopentanecarbonyl chloride (0.833 mmol) in acetone (10 mL) was added dropwise to the reaction mixture at 0 °C with continuous stirring. After complete addition, the reaction temperature was raised to room temperature, and stirring was continued for 4 h. The reaction mixture was filtered, and the filtered solid was washed with acetone (2 × 10 mL). The combined filtrate and wash were evaporated to dryness. The residue was dissolved in ethyl acetate (10 mL) and extracted with dilute HCl. The organic layer was then washed with saline (2 × 10 mL), and dried with anhydrous sodium sulfate. The organic solvent was evaporated under reduced pressure to get the intermediate title compounds. They were used in the next steps without further purification.

### 4.3 Synthesis of the target sulfonate compounds **1a**–**i**

A solution of compound **4a**,**b** (0.456 mmol) in dry THF (10 mL) was cooled to 0 °C, and triethylamine (0.25 mL, 2.47 mmol) was added thereto. A solution of the appropriate sulfonyl chloride (0.90 mmol) in dry THF (3 mL) was added dropwise to the reaction mixture at the same temperature. The reaction mixture was stirred at room temperature for 2 h. After reaction completion, the mixture was quenched with ethyl acetate (10 mL) and water (10 mL). The organic layer was separated, and the aqueous layer was extracted with ethyl acetate (3 × 5 mL). The combined organic layer extract were washed with saline (3 × 10 mL), and dried over anhydrous sodium sulfate. The organic solvent was evaporated under reduced pressure, and the crude residue was purified by column chromatography (silica gel, appropriate ratio of hexane/ethyl acetate) to obtain the pure product.

#### 4.3.1 4-(Cyclohexanecarboxamido)phenyl methanesulfonate (**1a**)

Yield: 87%; mp: 173–6 °C; IR (KBr disc, cm^−1^): 3324 (NH), 2931, 2853 (C—H stretching), 1668 (C=O), 1524, 1371 (OSO_2_); ^1^H NMR (400 MHz, CDCl_3_) *δ* 7.58 (d, 2H, Ar-H, *J* = 8.0 Hz), 7.43 (s, 1H, NH), 7.21 (d, 2H, Ar-H, *J* = 8.0), 3.12 (s, 3H, CH_3_), 2.28–2.20 (m, 1H, cyclohexyl-H), 1.94 (d, 2H, cyclohexyl-H, *J* = 12.0 Hz), 1.86–1.82 (m, 2H, cyclohexyl-H), 1.72–1.67 (m, 2H, cyclohexyl-H), 1.58–1.49 (m, 2H, cyclohexyl-H), 1.29–1.26 (m, 2H, cyclohexyl-H); ^13^C NMR (100 MHz, CDCl_3_) *δ* 174.6 (C=O), 145.0, 137.4, 122.5 (2C), 121.1 (2C) [Ar-C], 46.4, 37.2, 29.6 (2C), 25.6 (2C) [aliph. C]; LC–MS: *m*/*z* 298.07 [M^+^ +1].

#### 4.3.2 4-(Cyclohexanecarboxamido)phenyl ethanesulfonate (**1b**)

Yield: 85%; mp: 140–2 °C; IR (KBr disc, cm^−1^): 3309 (NH), 2924, 2853 (C—H stretching), 1660 (C=O), 1527, 1349 (OSO_2_); ^1^H NMR (400 MHz, CDCl_3_) *δ* 7.59 (br s, 1H, NH), 7.57 (d, 2H, Ar-H, *J* = 8.0 Hz), 7.19 (d, 2H, Ar-H, *J* = 12.0 Hz), 3.26 (q, 2H, C*H*_2_CH_3_, *J* = 8.0 Hz), 2.28–2.20 (m, 1H, aliph.-H), 1.94–1.91 (m, 3H, cyclohexyl-H), 1.85–1.81 (m, 2H, cyclohexyl-H), 1.71–1.68 (m, 1H, cyclohexyl-H), 1.52 (t, 3H, CH_2_C*H*_3_, *J* = 8.0 Hz), 1.28–1.26 (m, 3H); ^13^C NMR (100 MHz, CDCl_3_) *δ* 174.7 (C=O), 144.8, 137.3, 122.5 (2C), 121.1 (2C) [Ar-C], 46.4, 44,9, 29.6 (2C), 25.6 (2C), 8.2 [aliph. C]; LC–MS: *m*/*z* 312.24 [M^+^ +1].

#### 4.3.3 4-(Cyclohexanecarboxamido)phenyl propane-1-sulfonate (**1c**)

Yield: 86%; mp: 150–3 °C; IR (KBr disc, cm^−1^): 3313 (NH), 2924, 2853 (C—H stretching), 1661 (C=O), 1528, 1335 (OSO_2_); ^1^H NMR (400 MHz, CDCl_3_) *δ* 7.59 (br s, 1H, NH), 7.56 (d, 2H, Ar-H, *J* = 8.0 Hz), 7.18 (d, 2H, Ar-H, *J* = 8.0 Hz), 3.22–3.18 (m, 2H, aliph.-H), 2.28–2.20 (m, 1H), 2.03–2.00 (m, 1H, aliph.-H), 1.98 (d, 1H, aliph.-H, *J* = 8.0 Hz), 1.92 (d, 2H, cyclohexyl-H, *J* = 16.0 Hz), 1.84–1.77 (m, 2H, cyclohexyl-H), 1.71–1.69 (m, 1H, cyclohexyl-H), 1.58–1.48 (m, 2H, cyclohexyl-H), 1.27–1.25 (m, 4H, cyclohexyl-H), 1.11 (t, 3H, CH_2_CH_2_C*H*_3_, *J* = 8.0 Hz); ^13^C NMR (100 MHz, CDCl_3_) *δ* 174.8 (C=O), 144.8, 137.2, 122.5 (2C), 121.1 (2C) [Ar-C], 51.9, 46.3, 29.6 (2C), 25.6 (2C), 17.3, 12.8 [aliph. C]; LC–MS: *m*/*z* 326.0 [M^+^ +1].

#### 4.3.4 4-(Cyclohexanecarboxamido)phenyl benzenesulfonate (**1d**)

Yield: 80%; mp: 156–9 °C; IR (KBr disc, cm^−1^): 3319 (NH), 2927, 2854 (C—H stretching), 1665 (C=O), 1519, 1377 (OSO_2_); ^1^H NMR (400 MHz, CDCl_3_) *δ* 7.81–7.79 (m, 2H, Ar-H), 7.68–7.64 (m, 2H, Ar-H), 7.53–7.46 (m, 4H, Ar-H), 6.87 (d, 2H, NH, *J* = 8.0), 2.26–2.18 (m, 1H, cyclohexyl-H), 1.89 (d, 2H, cyclohexyl-H, *J* = 12.0 Hz), 1.80–1.77 (m, 2H, cyclohexyl-H), 1.67 (d, 1H, cyclohexyl-H, *J* = 8.0 Hz), 1.50 (d, 2H, cyclohexyl-H, *J* = 12.0 Hz), 1.26–1.21 (m, 3H, cyclohexyl-H); ^13^C NMR (100 MHz, CDCl_3_) *δ* 174.8 (C=O), 145.2, 137.4, 135.1, 134.3, 129.2 (2C), 128.5 (2C), 122.7 (2C), 120.7 (2C) [Ar-C], 46.4, 29.7 (2C), 29.6, 25.6 (2C), 25.5 [aliph. C]; LC–MS: *m*/*z* 360.2 [M^+^ +1].

#### 4.3.5 4-(Cyclohexanecarboxamido)phenyl 4-methylbenzenesulfonate (**1e**)

Yield: 88%; mp: 171–4 °C; IR (KBr disc, cm^−1^): 3740 (NH), 2927, 2855 (C—H stretching), 1656 (C=O), 1528, 1377 (OSO_2_); ^1^H NMR (400 MHz, CDCl_3_) *δ* 7.68 (d, 2H, Ar-H, *J* = 8.0 Hz), 7.45 (d, 2H, Ar-H, *J* = 8.0 Hz), 7.30 (d, 2H, Ar-H, *J* = 8.0 Hz), 7.25 (br s, 1H, NH), 6.90 (d, 2H, Ar-H, *J* = 8.0 Hz), 2.44 (s, 3H, CH_3_), 2.20–2.17 (m, 1H, cyclohexyl-H), 1.92 (d, 2H, cyclohexyl-H, *J* = 12.0 Hz), 1.85–1.81 (m, 2H, cyclohexyl-H), 1.71–1.68 (m, 1H, cyclohexyl-H), 1.55–1.46 (m, 2H, cyclohexyl-H), 1.32–1.30 (m, 2H, cyclohexyl-H); ^13^C NMR (100 MHz, CDCl_3_) *δ* 174.4 (C=O), 145.4, 137.0, 132.2, 129.8 (2C), 128.6 (2C), 122.9 (2C), 120.5 (2C) [Ar-C], 46.5, 29.6 (2C), 25.6 (2C), 21.7, 14.1 [aliph. C]; LC–MS: *m*/*z* 373.91 [M^+^ +1].

#### 4.3.6 4-(Cyclohexanecarboxamido)phenyl 4-(tert-butyl)benzenesulfonate (**1f**)

Yield: 85%; mp: 174–7 °C; IR (KBr disc, cm^−1^): 3369 (NH), 2956, 2922, 2851 (C—H stretching), 1671 (C=O), 1406, 1378 (OSO_2_); ^1^H NMR (400 MHz, CDCl_3_) *δ* 7.74 (d, 2H, Ar-H, *J* = 8.0 Hz), 7.52 (d, 2H, NH, *J* = 4.0 Hz), 7.46 (d, 2H, Ar-H, *J* = 8.0 Hz), 7.34 (br s, 1H, NH), 6.92 (d, 2H, Ar-H, *J* = 8.0 Hz) 2.25–2.17 (m, 1H, cyclohexyl-H), 1.92 (d, 2H, *J* = 12.0 Hz), 1.84–1.80 (m, 2H, cyclohexyl-H), 1.70–1.66 (m, 2H, cyclohexyl-H), 1.55–1.46 (m, 2H, cyclohexyl-H), 1.34 (s, 9H, *tert*-butyl-9H), 1.27–1.24 (m, 2H, cyclohexyl-H); ^13^C NMR (100 MHz, CDCl_3_) *δ* 174.5 (C=O), 145.4, 137.1, 132.2, 128.4 (2C), 126.2 (2C), 122.9 (2C), 120.5 (2C) [Ar-C], 46.5, 29.6 (2C), 25.6 (3C) [aliph. C]. LC–MS: *m*/*z* 416.21 [M^+^ +1].

#### 4.3.7 4-(Cyclohexanecarboxamido)phenyl 4-fluorobenzenesulfonate (**1g**)

Yield: 87%; mp: 154–5 °C; IR (KBr disc, cm^−1^): 3316 (NH), 2929, 2853 (C—H stretching), 1665 (C=O), 1519, 1379 (OSO_2_); ^1^H NMR (400 MHz, CDCl_3_) *δ* 7.85–7.81(m, 2H, Ar-H), (d, 2H, Ar-H, *J* = 8.0 Hz), 7.47 (d, 2H, Ar-H, *J* = 8.0 Hz), 7.27 (br s, 1H, NH), 7.22–7.17 (m, 2H), 6.91 (d, 2H, Ar-H, *J* = 8.0 Hz), 2.44 (s, 3H, CH_3_), 2.25–2.17 (m, 1H, cyclohexyl-H), 1.93 (d, 2H, cyclohexyl-H, *J* = 12.0 Hz), 1.85–1.81 (m, 2H, cyclohexyl-H), 1.71–1.69 (m, 1H, cyclohexyl-H), 1.57–1.47 (m, 2H, cyclohexyl-H), 1.35–1.21 (m, 3H, cyclohexyl-H); ^13^C NMR (100 MHz, CDCl_3_) *δ* 174.5 (C=O), 145.2, 137.2, 131.5 (2C), 131.4, 122.9 (2C), 120.6, 116.7 (2C), 116.5 (2C) [Ar-C], 46.5, 29.6 (2C), 25.6 (3C) [aliph. C]; LC–MS: *m*/*z* 378.23 [M^+^ +1].

#### 4.3.8 4-(Cyclohexanecarboxamido)phenyl 4-(trifluoromethyl)benzenesulfonate (**1h**)

Yield: 85%; mp: 171–2 °C; IR (KBr disc, cm^−1^): 3327 (NH), 2931, 2850 (C—H stretching), 1661 (C=O), 1407, 1386 (OSO_2_); ^1^H NMR (400 MHz, CDCl_3_) *δ* 7.96 (d, 2H, Ar-H, *J* = 8.0 Hz), 7.80 (d, 2H, Ar-H, *J* = 8.0 Hz), 7.49 (d, 2H, Ar-H, *J* = 8.0 Hz), 7.36 (br s, 1H, NH), 6.92 (d, 2H, Ar-H, *J* = 8.0 Hz), 2.25–2.18 (m, 1H, cyclohexyl-H), 1.92 (d, 2H, cyclohexyl-H, *J* = 12.0 Hz), 1.85–1.81 (m, 2H, cyclohexyl-H), 1.71–1.68 (m, 2H, cyclohexyl-H), 1.56–1.47 (m, 2H, cyclohexyl-H), 1.31–1.24 (m, 2H, cyclohexyl-H); ^13^C NMR (100 MHz, CDCl_3_) *δ* 174.6 (C=O), 145.0, 138.8, 137.5, 136.0, 129.1 (2C), 126.4 (2C), 126.3, 122.7 (2C), 120.7 (2C) [Ar-C], 46.5, 29.6 (2C), 25.6 (3C) [aliph. C]; LC–MS: *m*/*z* 427.94 [M^+^ +1].

#### 4.3.9 4-(Cyclopentanecarboxamido)phenyl 4-methylbenzenesulfonate (**1i**)

Yield: 80%; mp: 151–3 °C; IR (KBr disc, cm^−1^): 3731 (NH), 2917, 2845 (C—H stretching), 1655 (C=O), 1527, 1375 (OSO_2_); ^1^H NMR (400 MHz, CDCl_3_) *δ* 7.69 (d, 2H, Ar-H, *J* = 12.0 Hz), 7.62 (br s, 1H, NH), 7.47 (d, 2H, Ar-H, *J* = 8.0 Hz), 7.31 (d, 2H, Ar-H, *J* = 8.0 Hz), 6.89 (d, 2H, Ar-H, *J* = 8.0 Hz), 2.71–2.63 (m, 1H, cyclopentyl-H), 2.45 (s, 3H, CH_3_), 1.92–1.74 (m, 6H, cyclopentyl-H), 1.61–1.57 (m, 2H, cyclopentyl-H); ^13^C NMR (100 MHz, CDCl_3_) *δ* 175.0 (C=O), 145.5, 145.2, 137.3, 132.1, 129.8 (2C), 128.5 (2C), 122.8 (2C), 120.5 (2C) [Ar-C], 46.4, 30.5 (2C), 26.0 (2C), 21.7 [aliph. C]; LC–MS: *m*/*z* 359.75 [M^+^ +1].

### 4.4 Synthesis of the target sulfamate compounds **1j**–**m**

A solution of compound **4a**,**b** (0.456 mmol) in dry DMF (10 mL) was cooled to 0 °C, and NaH (60% dispersion in mineral oil, 18.2 mg, 0.456 mmol) was added thereto under nitrogen atmosphere. A solution of the appropriate sulfamoyl chloride (2.0 mmol) in dry DMF (3 mL) was added dropwise to the reaction mixture at the same temperature. The reaction mixture was stirred at room temperature overnight. After reaction completion, the mixture was quenched with ethyl acetate (10 mL) and water (10 mL). The organic layer was separated, and the aqueous layer was extracted with ethyl acetate (3 × 5 mL). The combined organic layer extract were washed with saline (3 × 10 mL), and dried over anhydrous sodium sulfate. The organic solvent was evaporated under reduced pressure, and crude residue was purified by column chromatography (silica gel, appropriate ratio of hexane/ethyl acetate) to obtain the pure product.

#### 4.4.1 4-(Cyclohexanecarboxamido)phenyl sulfamate (**1j**)

Yield: 83%; mp: 174–6 °C; IR (KBr disc, cm^−1^): 3393 (NH), 3299 (NH_2_), 2932, 2855 (C—H stretching), 1661 (C=O), 1532, 1374 (OSO_2_); ^1^H NMR (400 MHz, CDCl_3_) *δ* 7.63 (d, 2H, Ar-H, *J* = 12.0 Hz), 7.27 (d, 2H, Ar-H, *J* = 8.0 Hz), 2.42–2.35 (m, 1H, aliph.-H), 1.92–1.74 (m, 5H, aliph.-H), 1.60–1.50 (m, 2H), 1.41–1.27 (m, 2H, aliph.-H); ^13^C NMR (100 MHz, CDCl_3_) *δ* 176.3 (C=O), 146.5, 137.2, 122.3 (2C), 120.8 (2C) [Ar-C], 45.7, 29.3 (2C), 25.5, 25.4 (2C) [aliph. C]; LC–Ms: *m*/*z* 299.08 [M^+^ +1].

#### 4.4.2 4-(Cyclohexanecarboxamido)phenyl methylsulfamate (**1k**)

Yield: 90%; mp: 162–5 °C; IR (KBr disc, cm^−1^): 3364 (NH), 3177 (NH), 2936, 2853 (C—H stretching), 1671 (C=O), 1538, 1340 (OSO_2_); ^1^H NMR (400 MHz, CDCl_3_) *δ* 7.63 (d, 2H, Ar-H, *J* = 8.0 Hz), 7.25 (d, 2H, Ar-H, *J* = 8.0 Hz), 2.81 (s, 3H, CH_3_), 2.42–2.35 (m, 1H, aliphatic C—H), 1.92–1.84 (m, 4H, aliphatic C—H), 1.77–1.74 (m, 1H, aliphatic C—H), 1.60–1.50 (m, 2H, aliphatic C—H), 1.44–1.28 (m, 3H, aliphatic C—H); ^13^C NMR (100 MHz, CDCl_3_) *δ* 176.3 (C=O), 146.2, 137.3, 122.8 (2C), 120.9(2C) [Ar-C], 45.7 (CH_3_), 29.3 (2C), 28.5, 25.5, 25.4 (2C) [aliph. C]; LC–Ms: *m*/*z* 312.99 [M^+^ +1].

#### 4.4.3 4-(Cyclohexanecarboxamido)phenyl dimethylsulfamate (**1l**)

Yield: 89%; mp: 155–8 °C; IR (KBr disc, cm^−1^): 3333 (NH), 2926, 2851 (C—H stretching), 1661 (C=O), 1522, 1365 (OSO_2_); ^1^H NMR (400 MHz, CDCl_3_) *δ* 8.07 (br s, 1H, NH), 7.56 (d, 2H, Ar-H, *J* = 8.0 Hz), 7.17–7.15 (m, 2H, Ar-H), 2.93 (s, 6H, N(CH_3_)_2_), 2.29–2.21 (m, 1H,aliphatic C—H), 1.89 (d, 2H, aliphatic C—H, *J* = 8.0 Hz), 1.79 (d, 2H, aliphatic C—H, *J* = 4.0 Hz), 1.67 (s, 1H,aliphatic C—H) 1.52–1.47 (m, 2H,aliphatic C—H), 1.24 (d, 3H,aliphatic C—H, *J* = 8.0 Hz); ^13^C NMR (100 MHz, CDCl_3_) *δ* 175.1 (C=O), 145.0, 137.2, 122.1 (2C), 121.0 (2C) [Ar-C], 46.2, 38.7, 29.6 (2C), 25.6 (2C), 25.5 (2C) [aliph. C]; LC–Ms: *m*/*z* 327.22 [M^+^ +1].

#### 4.4.4 4-(Cyclopentanecarboxamido)phenyl methylsulfamate (**1m**)

Yield: 89%; mp: 142–4 °C; IR (KBr disc, cm^−1^): 3288 (NH), 2925, 2855 (C—H stretching), 1660 (C=O), 1540, 1506 (OSO_2_); ^1^H NMR (400 MHz, CDCl_3_) *δ* 7.64 (d, 2H, Ar-H, *J* = 8.0 Hz), 7.25 (d, 2H, Ar-H, *J* = 8.0 Hz), 2.81 (s, 3H, CH_3_), 1.98–1.94 (m, 2H, cyclohexyl-H), 1.87–1.74 (m, 4H, cyclohexyl-H), 1.69–1.65 (m, 2H, cyclohexyl-H);^13^C NMR (100 MHz, CDCl_3_) *δ* 176.4 (C=O), 146.2, 137.3, 122.0 (2C), 120.0 (2C) [Ar-C], 45.8, 30.2 (2C), 28.5, 25.7 (2C) [aliph. C]; LC–MS: *m*/*z* 298.95 [M^+^ +1].

### 4.5 Biology

STS inhibitory assays were performed as described previously.^26^ A compound’s ability to inhibit STS activity was determined using the lysate of JEG-3, a human placenta choriocarcinoma cell line. To determine STS inhibition, activity was measured in the presence of the inhibitor (0.5–10 μM) using [^3^H]E_1_S (4 × 10^5^ dpm, Perkin Elmer) adjusted to 20 μM with unlabelled E_1_S substrate. After incubation of the substrate-inhibitor with JEG-3 lysate (125 μg of protein/mL) for 1 h, the product formed was isolated from the mixture by extraction with toluene (4 mL), using [4-^14^C]E_1_ (American Radiolabeled Chemicals) to monitor procedural losses.

Intact monolayers of JEG-3 cells were incubated for 20 h at 37 °C with [^3^H]E1S (5 pmol, 7 × 10^5^ dpm, 60 Ci/mmol) in serum-free Eagle’s Minimal Essential Medium (1.0 mL) with or without inhibitors (10^−11^–100 μM). After incubation, medium (0.5 mL) was removed and product estrone separated from E_1_S by solvent partition using toluene (4 mL). [^14^C]Estrone (7 × 10^3^ dpm, 52 mCi/mmol) was used to correct for procedural losses. An aliquot of the organic phase was added to scintillation fluid and the ^3^H and ^14^C content measured by scintillation spectrometry. The mass of E_1_S hydrolyzed was calculated from the ^3^H counts detected (corrected for the volume of medium and organic solvent used and for recovery of ^14^C counts) and the specific activity of the substrate.

## Supplementary data

Supplementary data IR, ^1^H NMR, ^13^C NMR, and LC–MS charts of the final compounds.
