# Supplementary material for: Design, synthesis, and biological evaluation of new arylamide derivatives possessing sulfonate or sulfamate moieties as steroid sulfatase enzyme inhibitors
Source: Bioorg Med Chem. 2016 Jun 15;24(12):2762–7. doi: 10.1016/j.bmc.2016.04.040 (PMC4896991; doi:10.1016/j.bmc.2016.04.040)

**Design, synthesis, and biological evaluation of new arylamide derivatives possessing sulfonate or sulfamate moieties as steroid sulfatase enzyme inhibitors**

**Mohammed I. El-Gamal ^a,b,c,*^, Mohammad H. Semreen ^a,b^, Paul A. Foster ^d,e,*^, and Barry V.L. Potter ^f^**

^a^ Department of Medicinal Chemistry, College of Pharmacy, University of Sharjah, Sharjah 27272, United Arab Emirates.

^b^ Sharjah Institute for Medical Research, University of Sharjah, Sharjah 27272, United Arab Emirates.

^c^ Department of Medicinal Chemistry, Faculty of Pharmacy, University of Mansoura, Mansoura 35516, Egypt.

^d^ Institute of Metabolism and Systems Research, University of Birmingham, Birmingham B15 2TT, United Kingdom.

^e^ Centre for Endocrinology, Diabetes and Metabolism, Birmingham Health Partners, Birmingham, B15 2HT, United Kingdom.

^f^ Department of Pharmacology, University of Oxford, Mansfield Road, Oxford OX13QT, United Kingdom.

^*^ E-mail addresses of the corresponding authors: [drmelgamal2002@gmail.com](mailto:drmelgamal2002@gmail.com) (M.I. El-Gamal) & [P.A.Foster@bham.ac.uk](mailto:P.A.Foster@bham.ac.uk) (P.A. Foster).


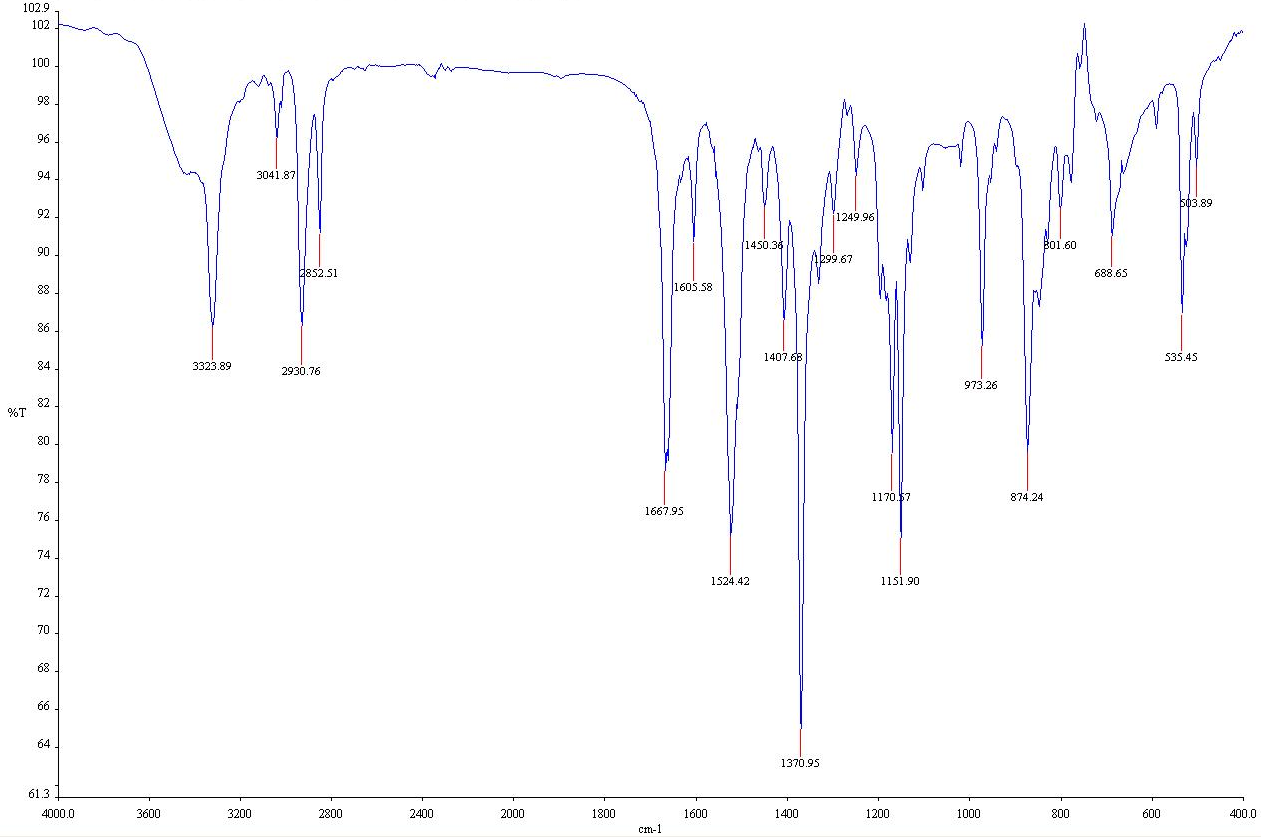


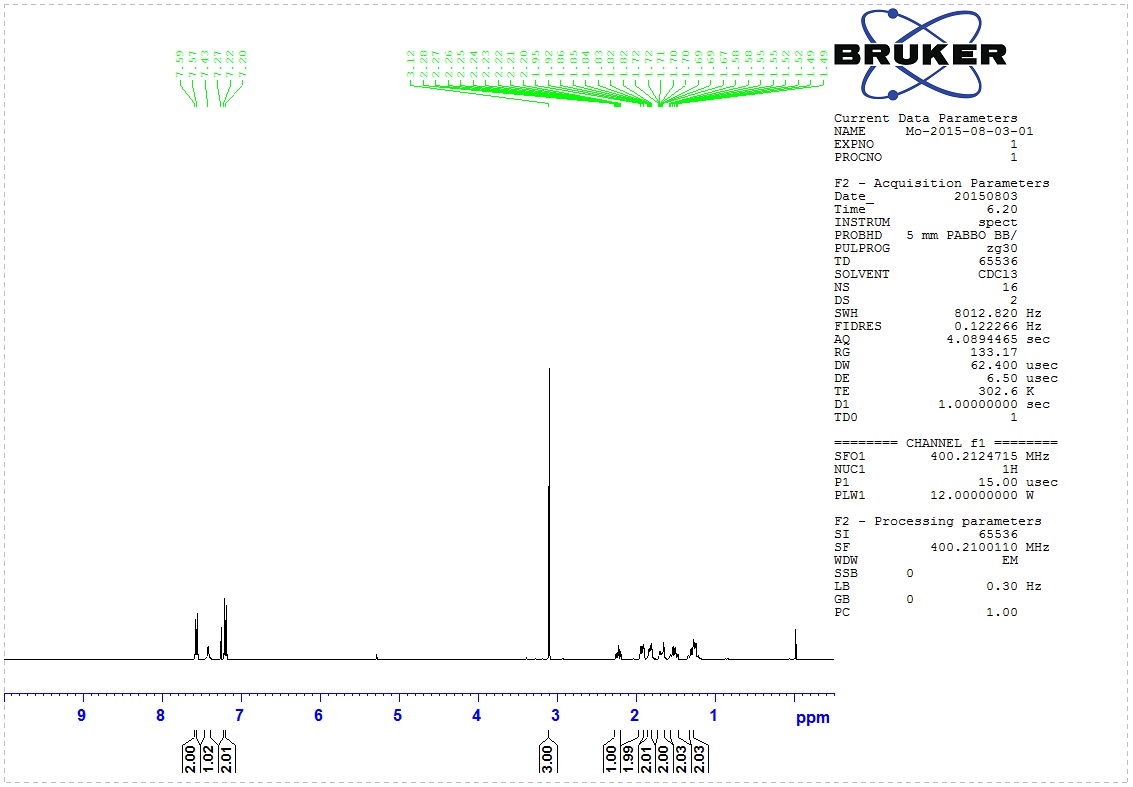


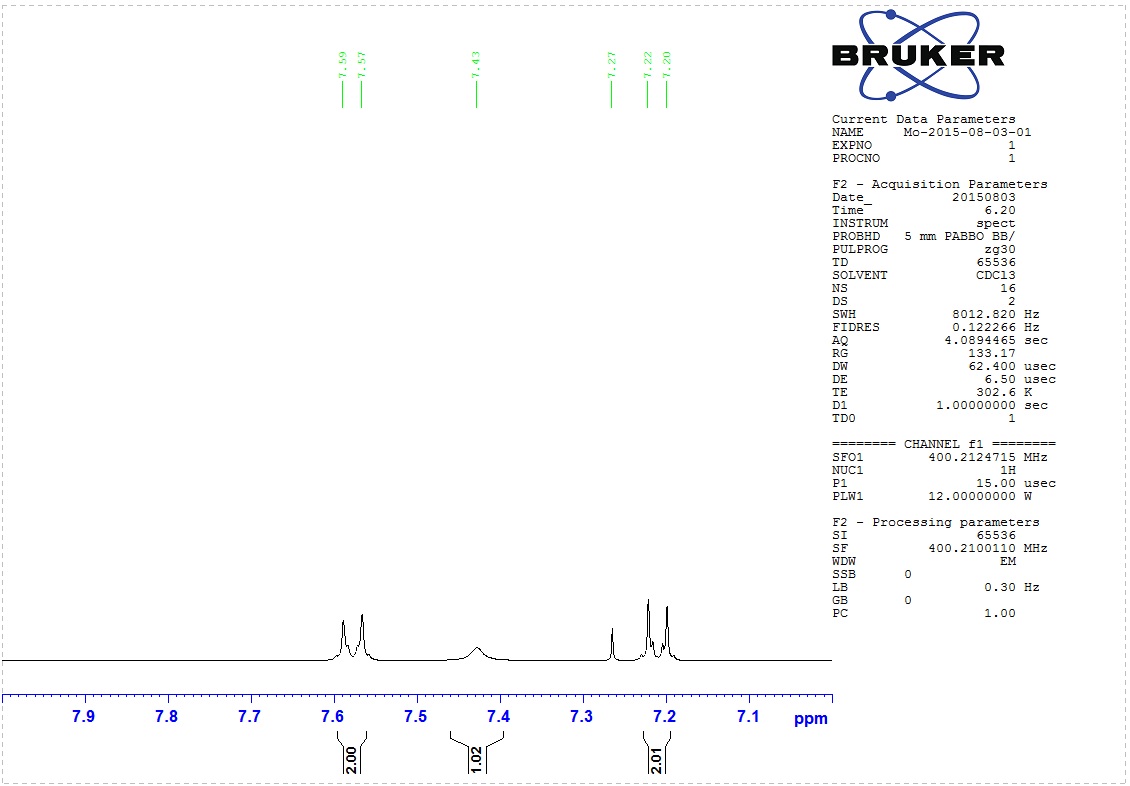


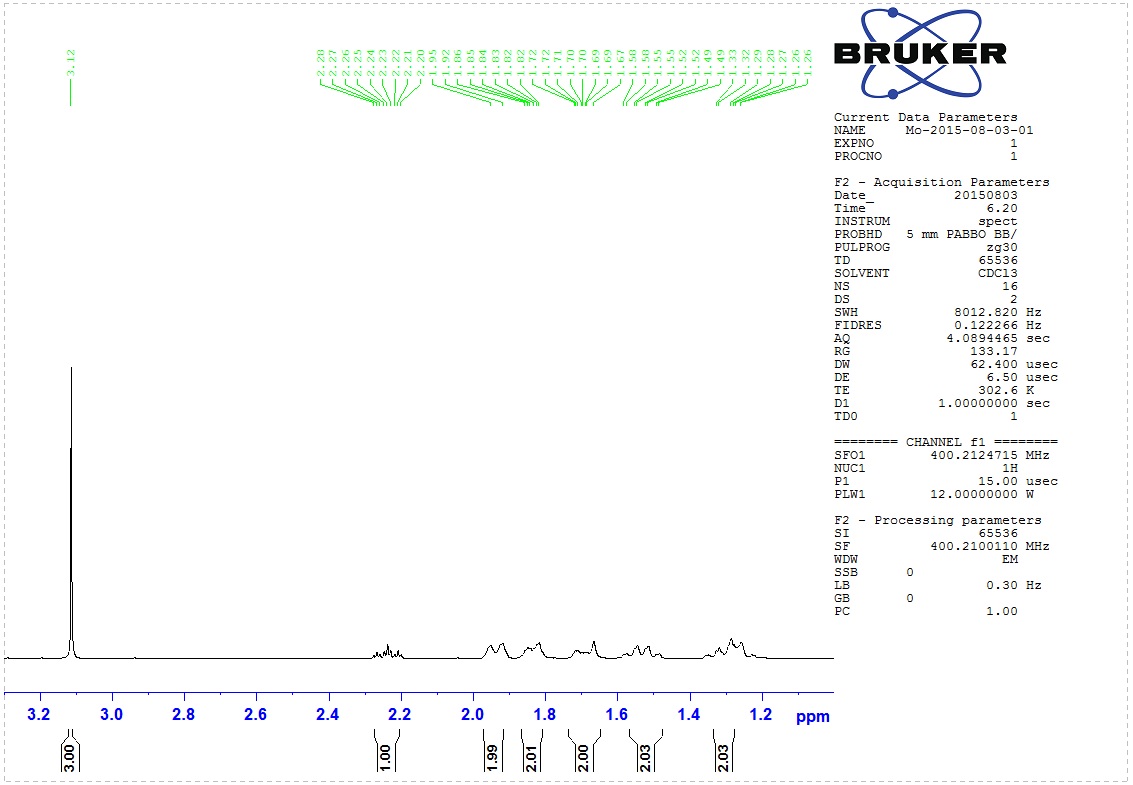


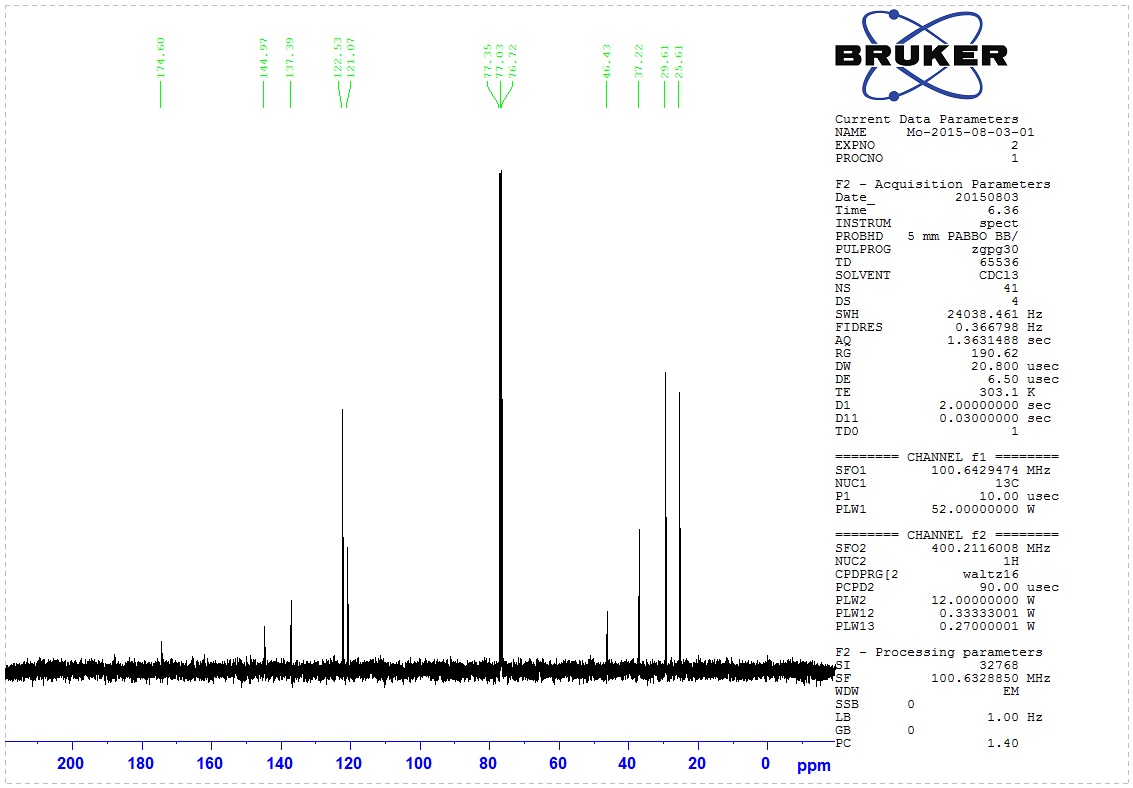


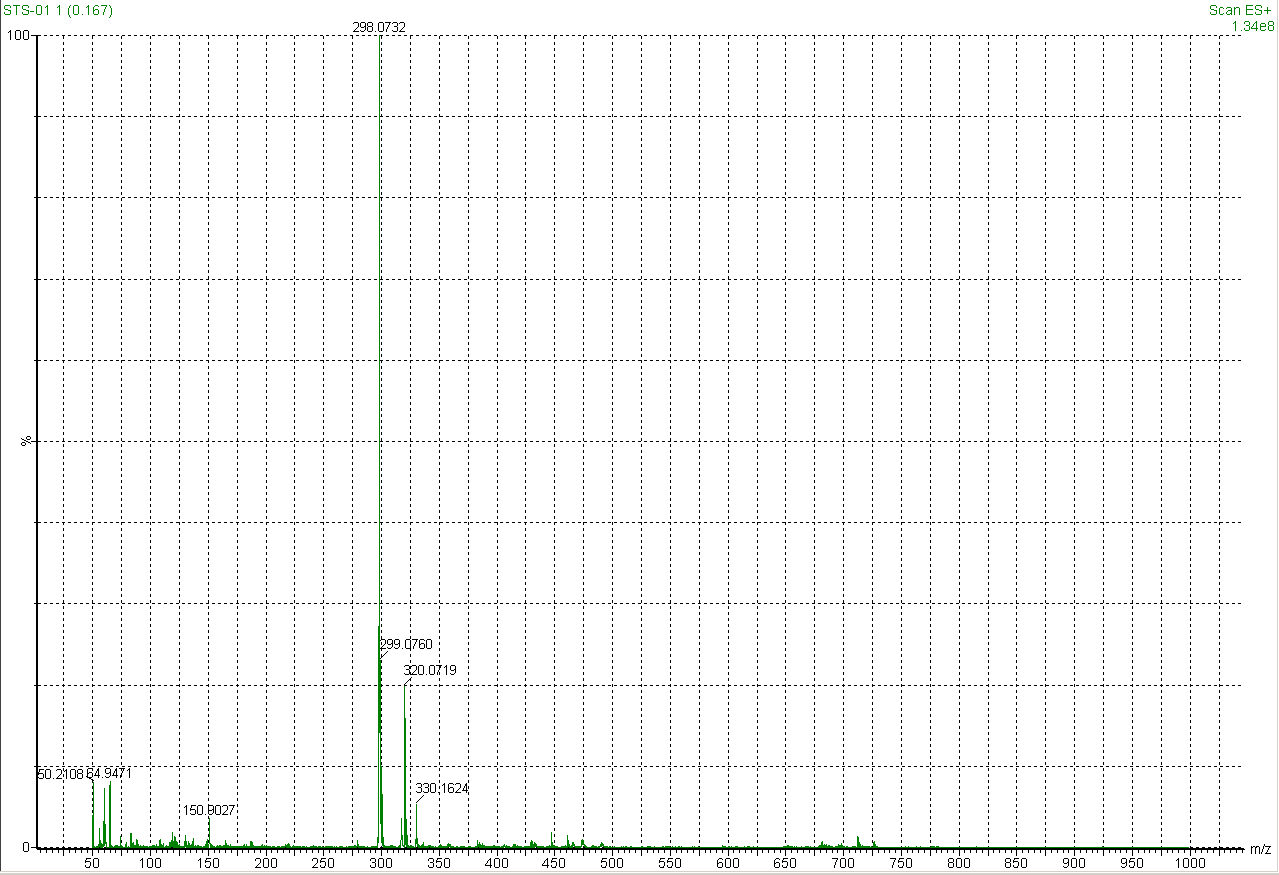


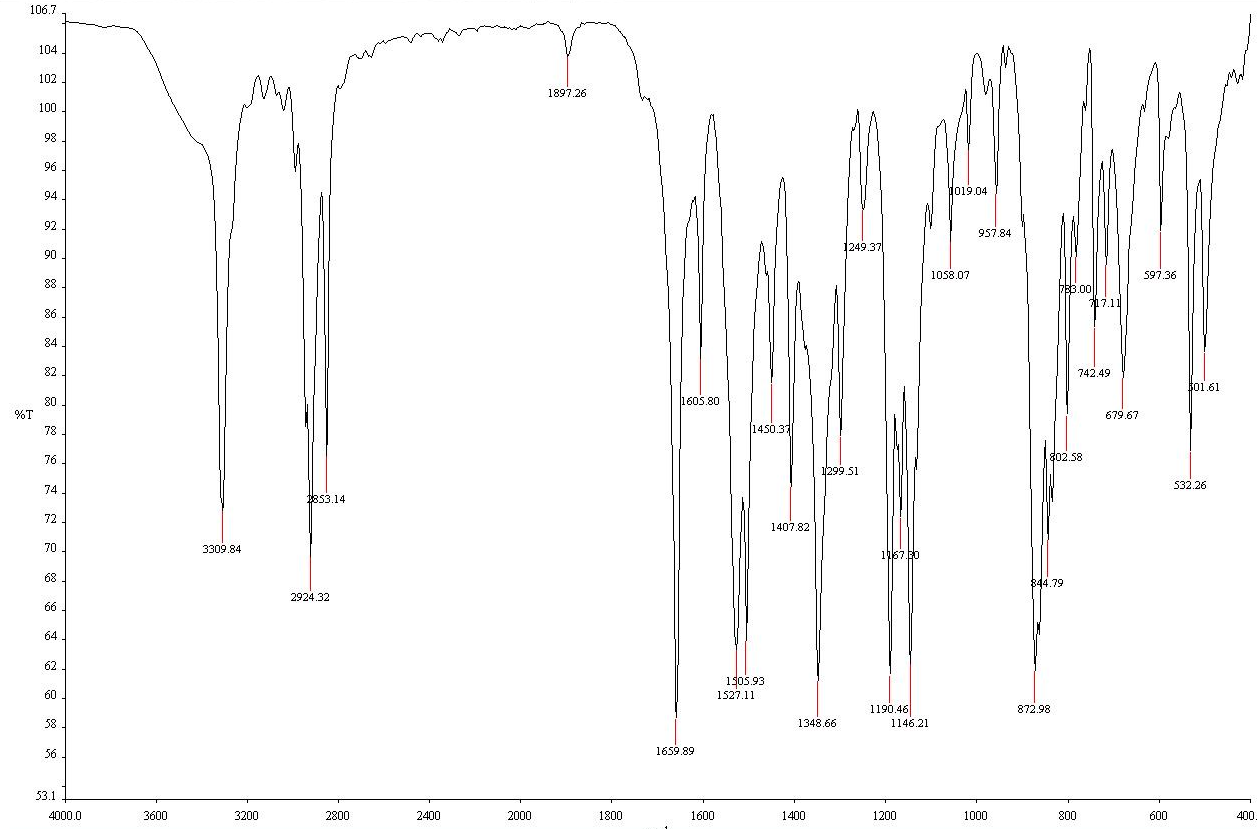


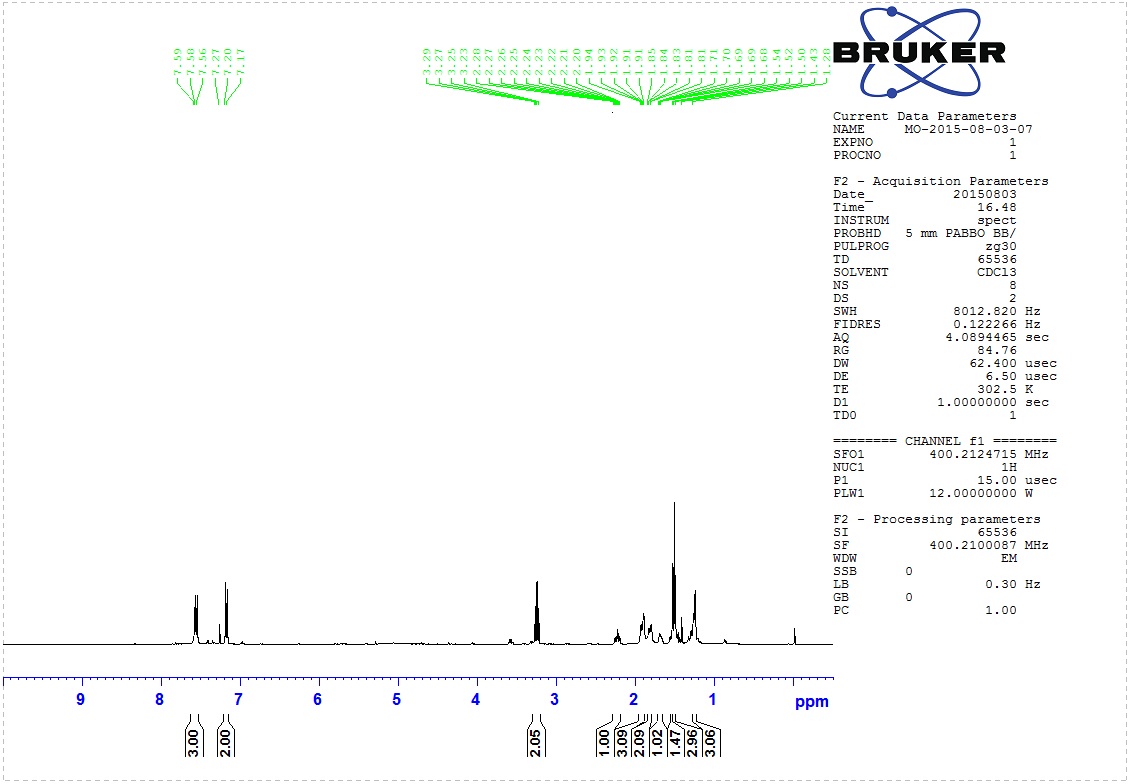


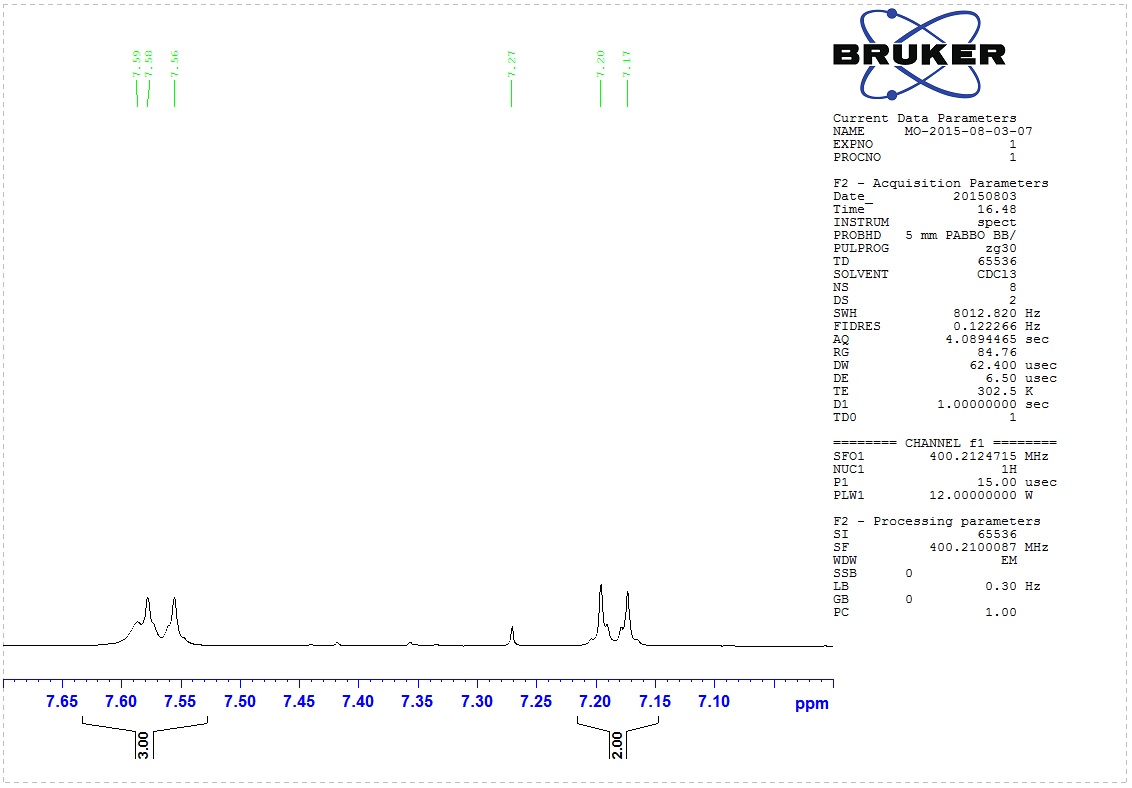


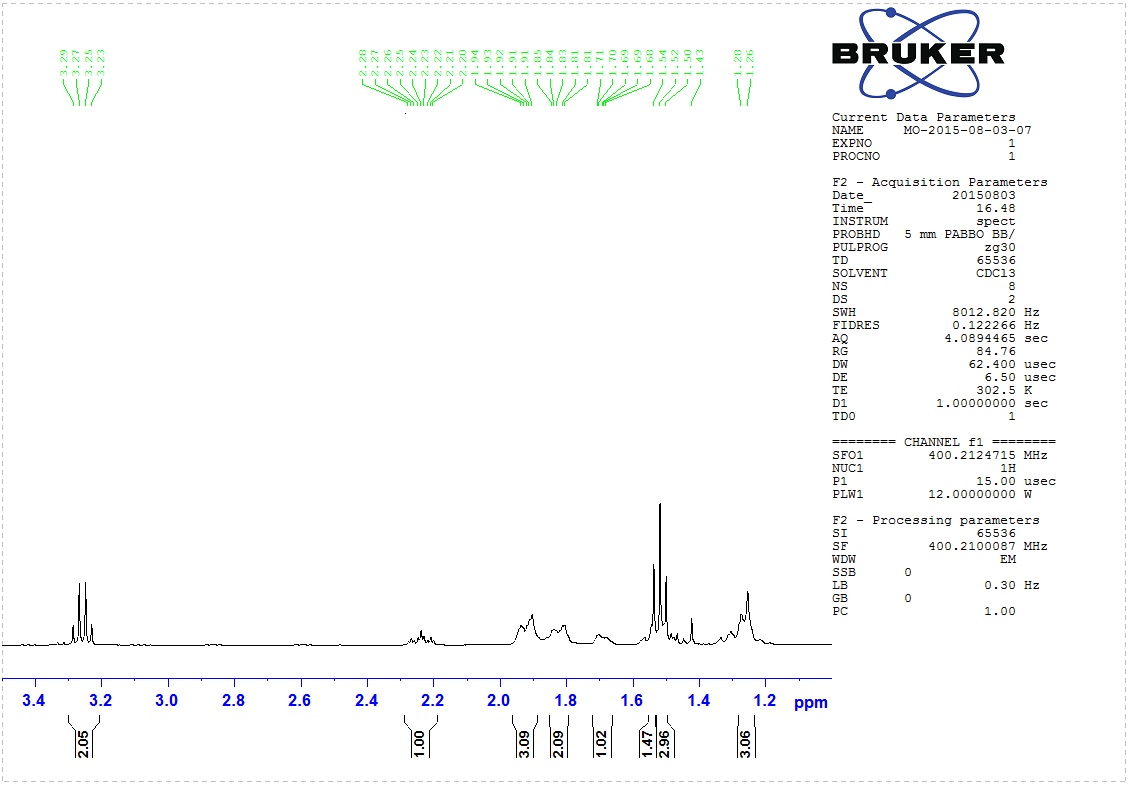


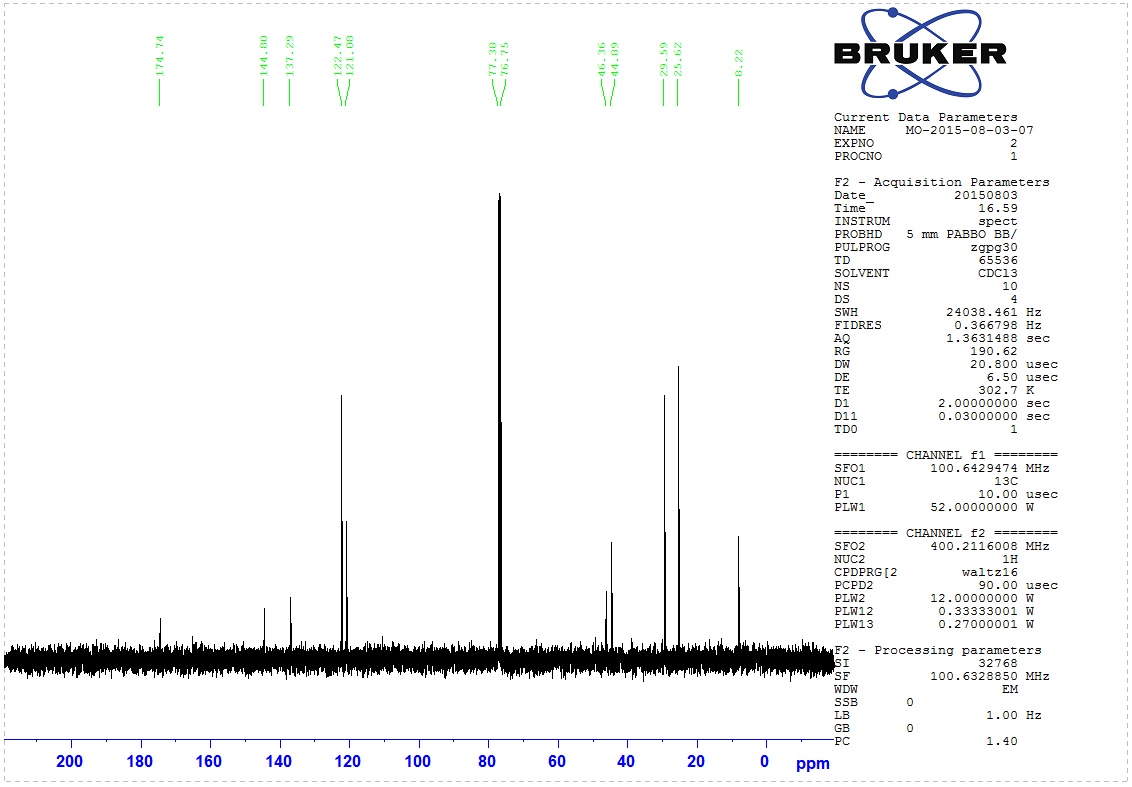


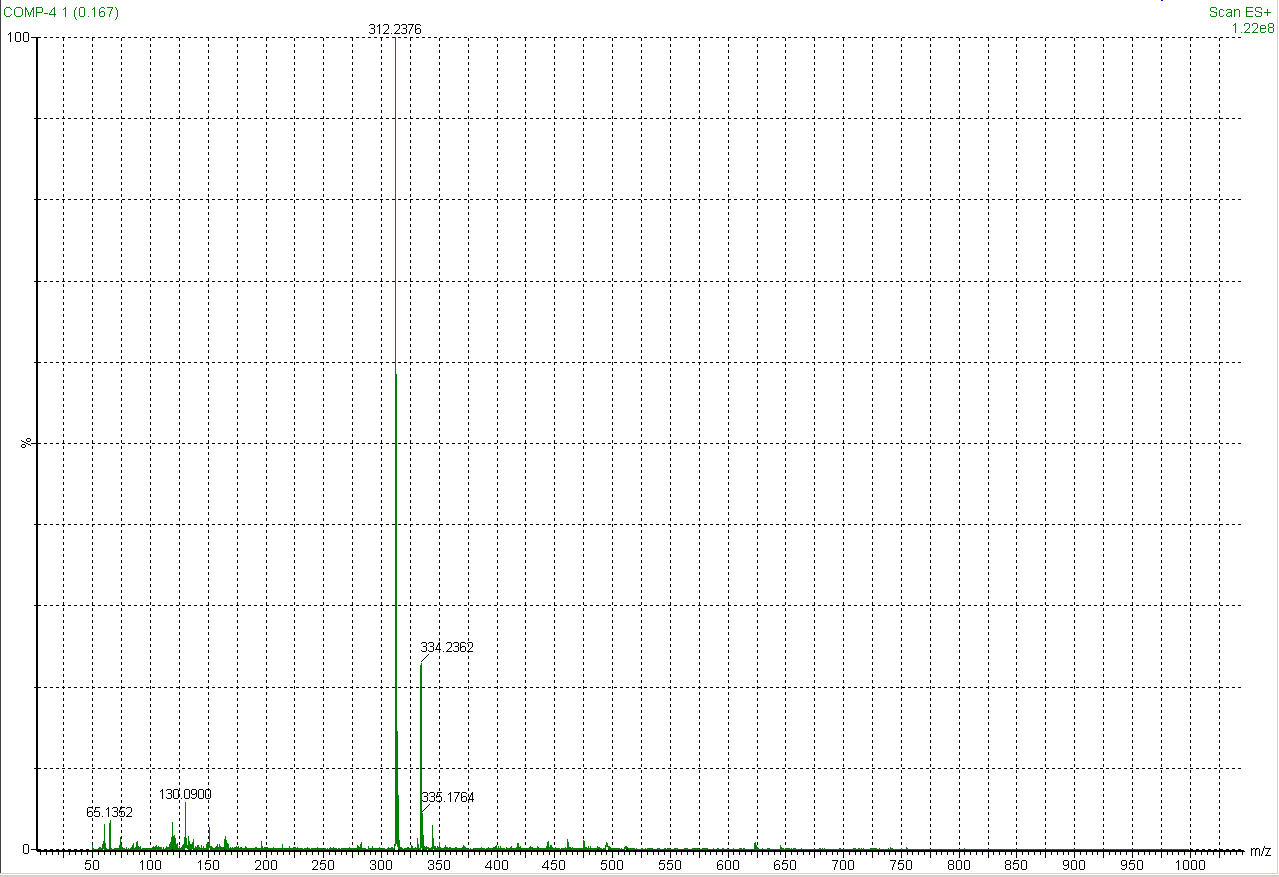


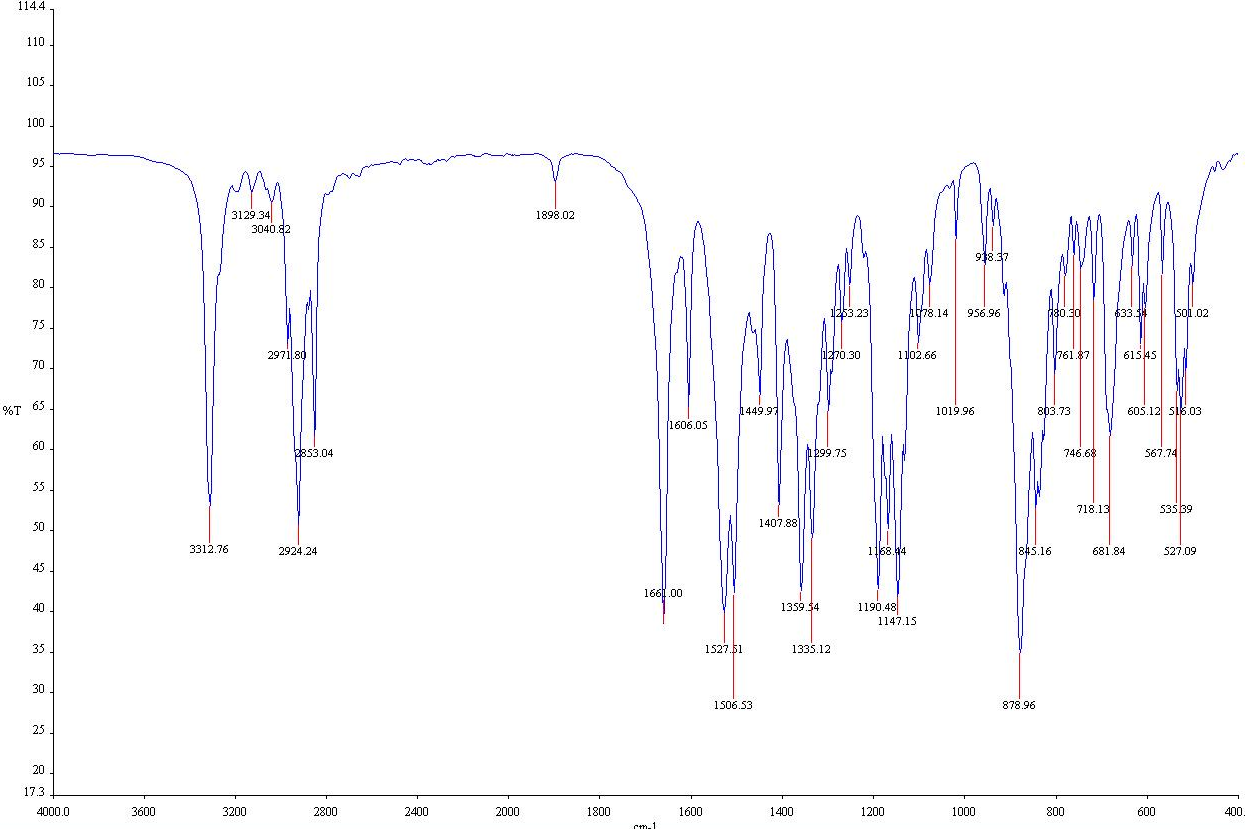


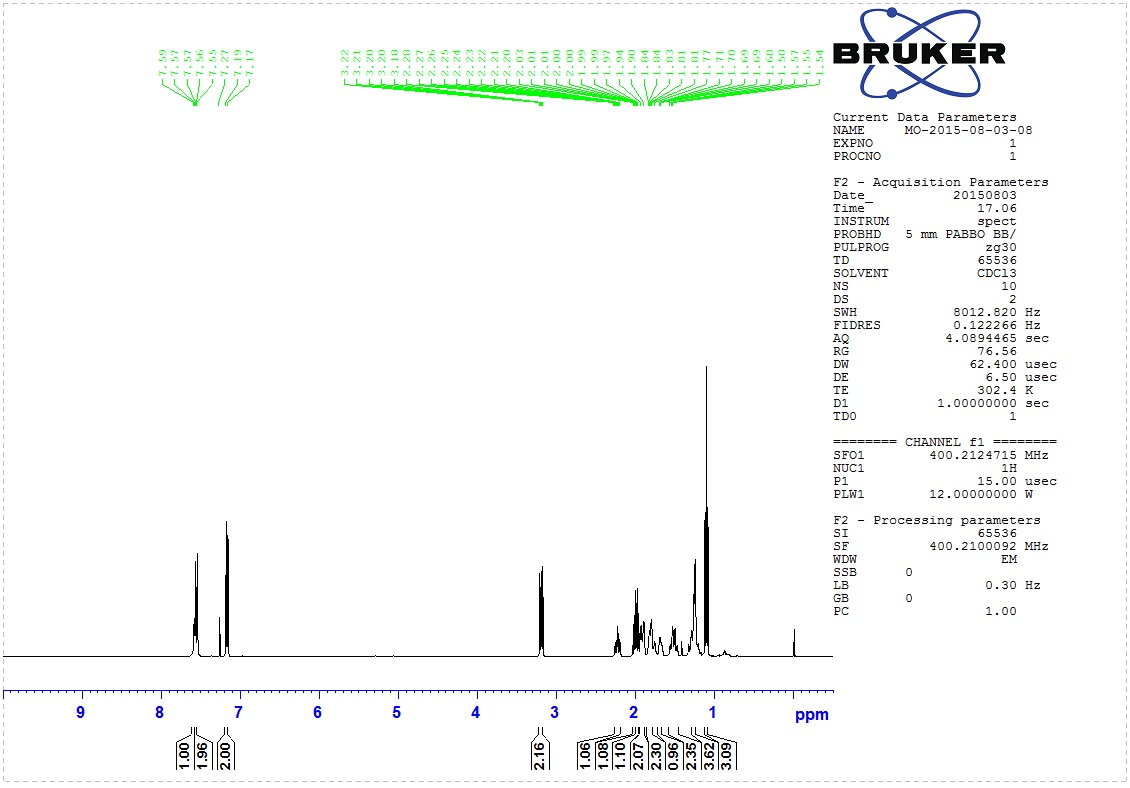


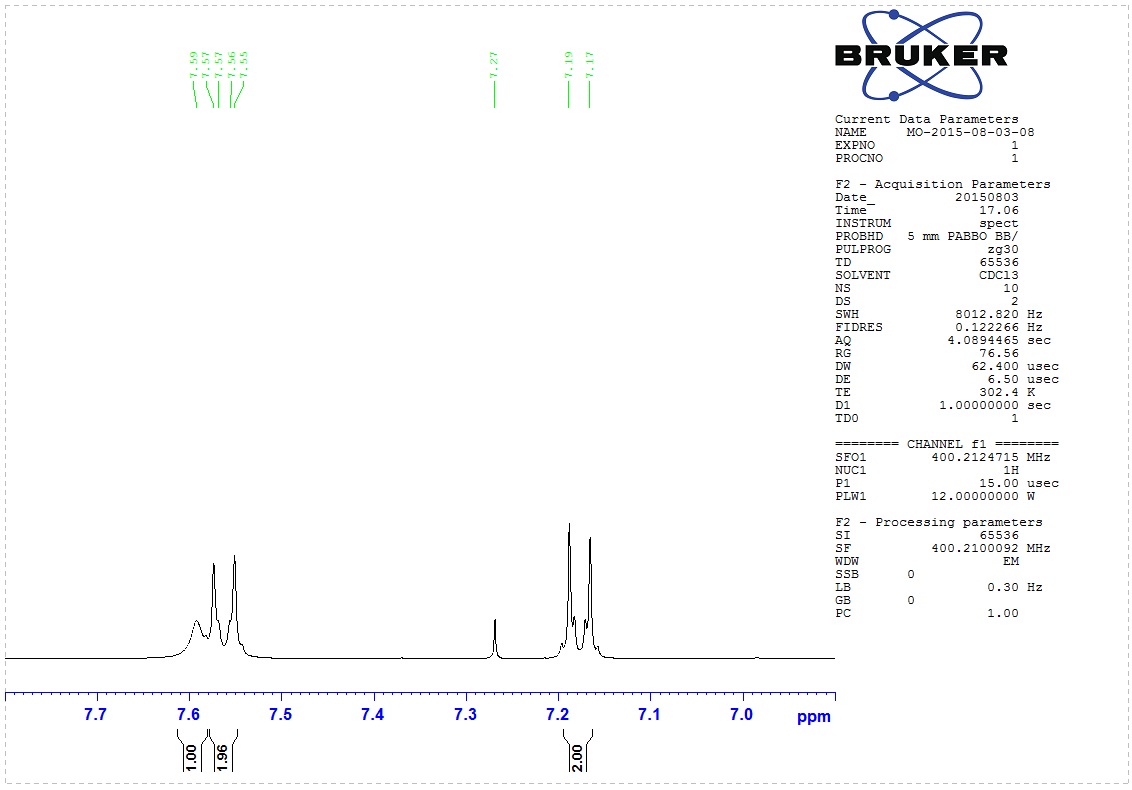


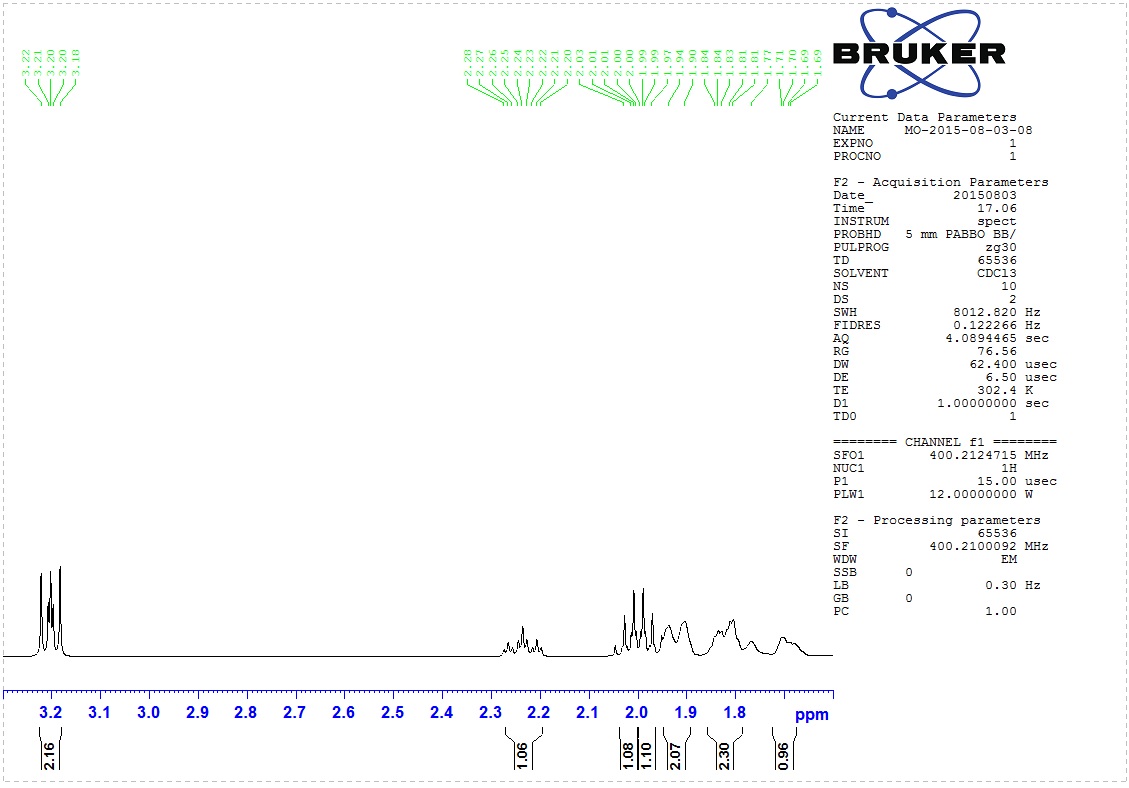


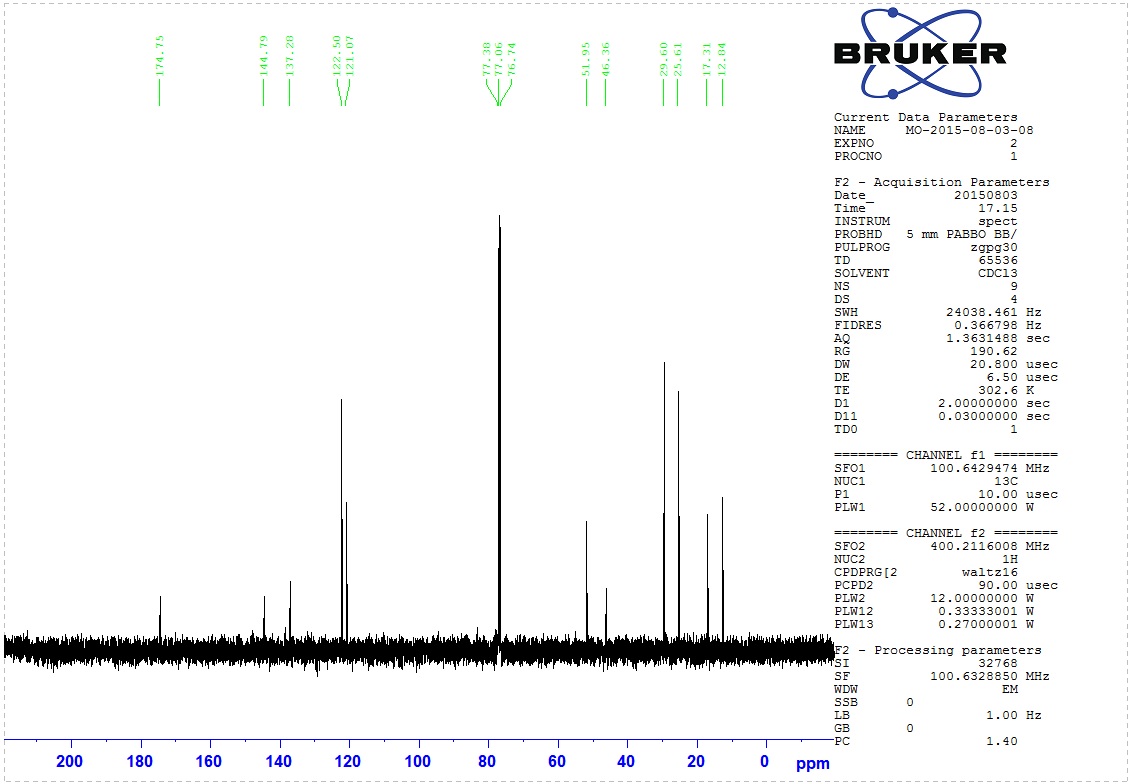


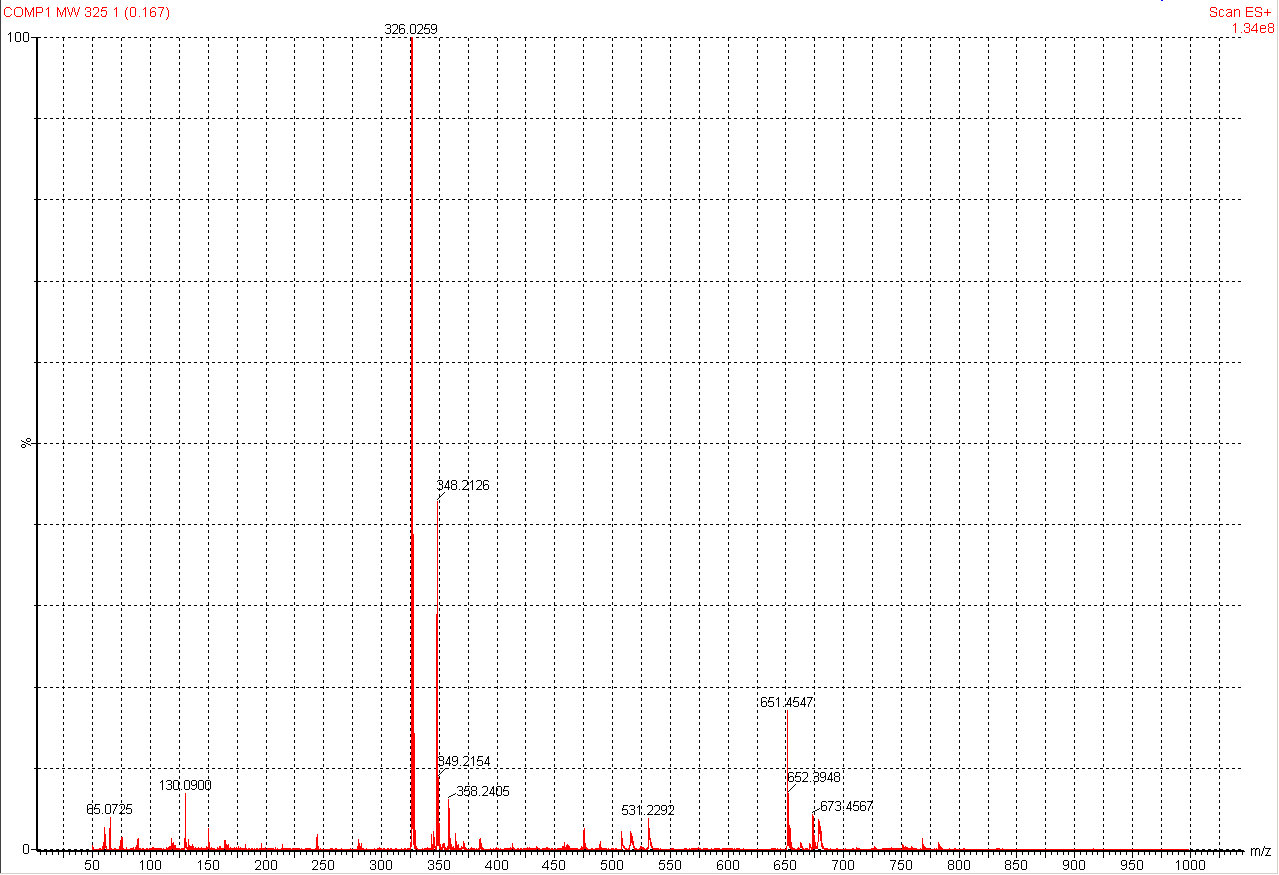


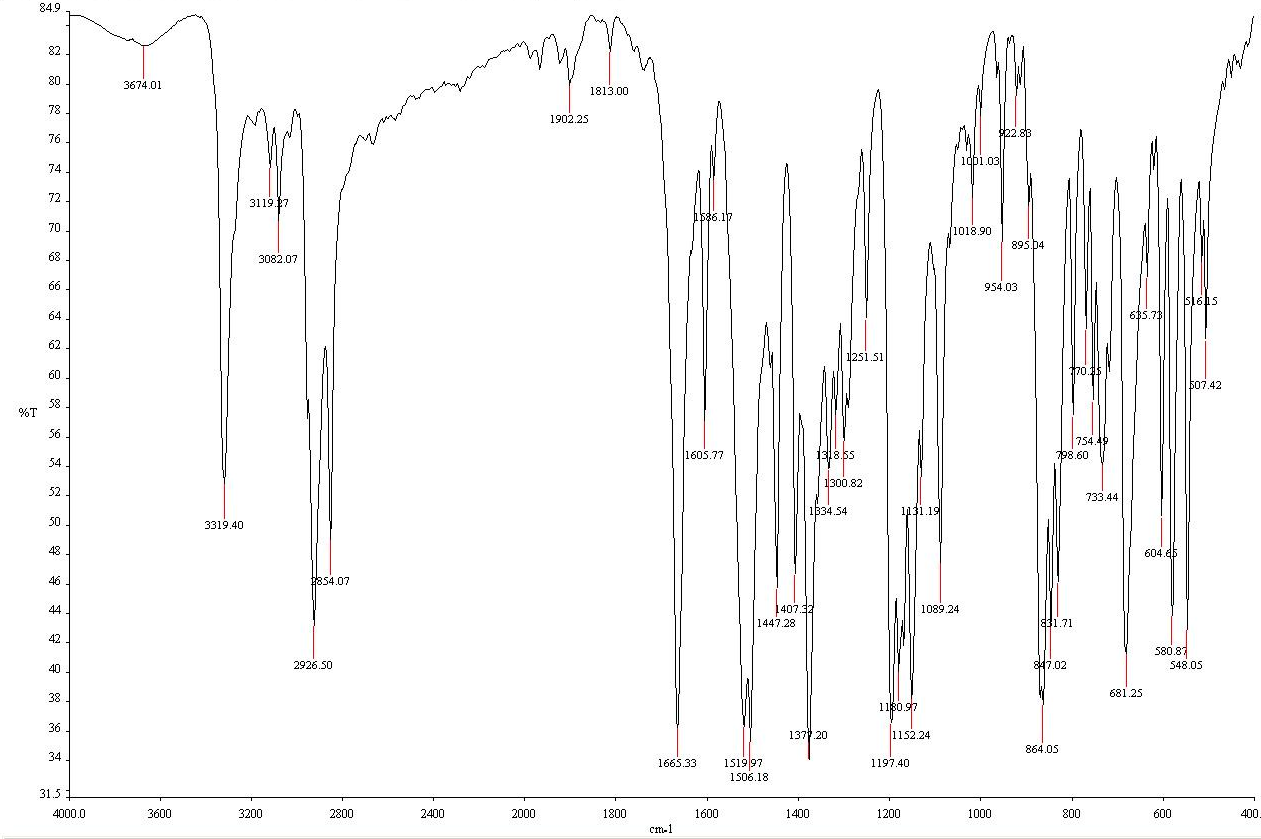


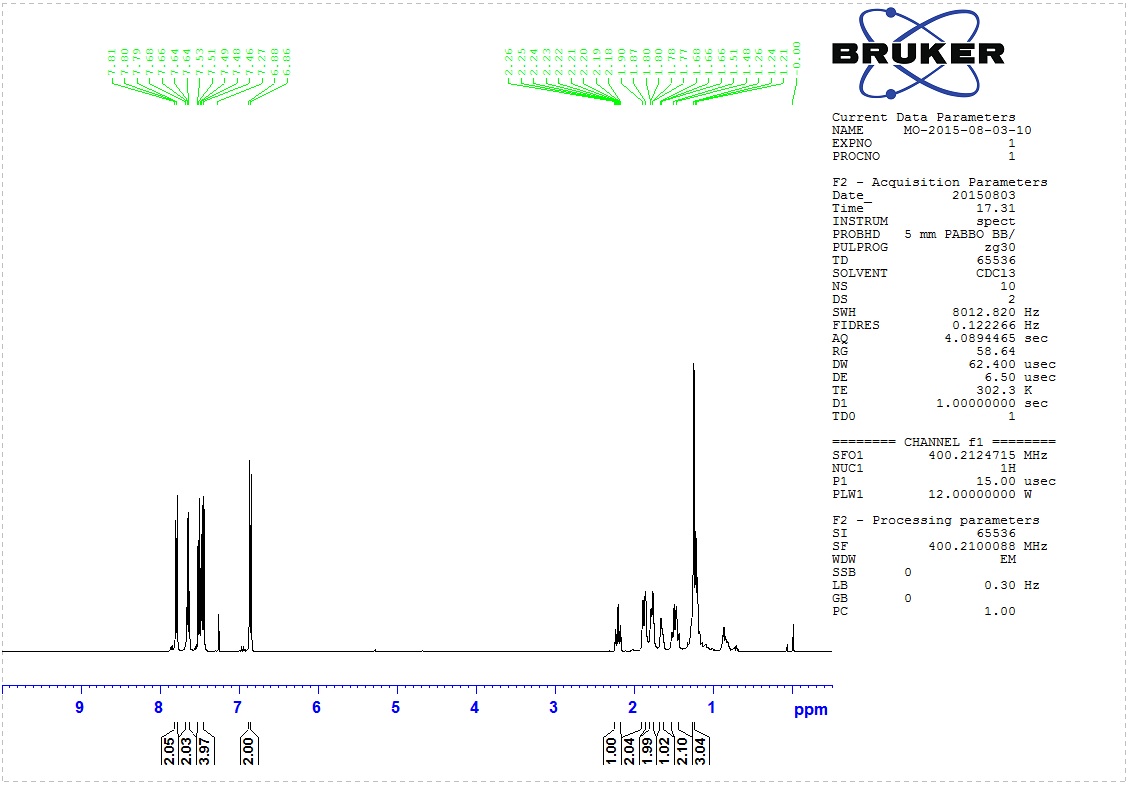


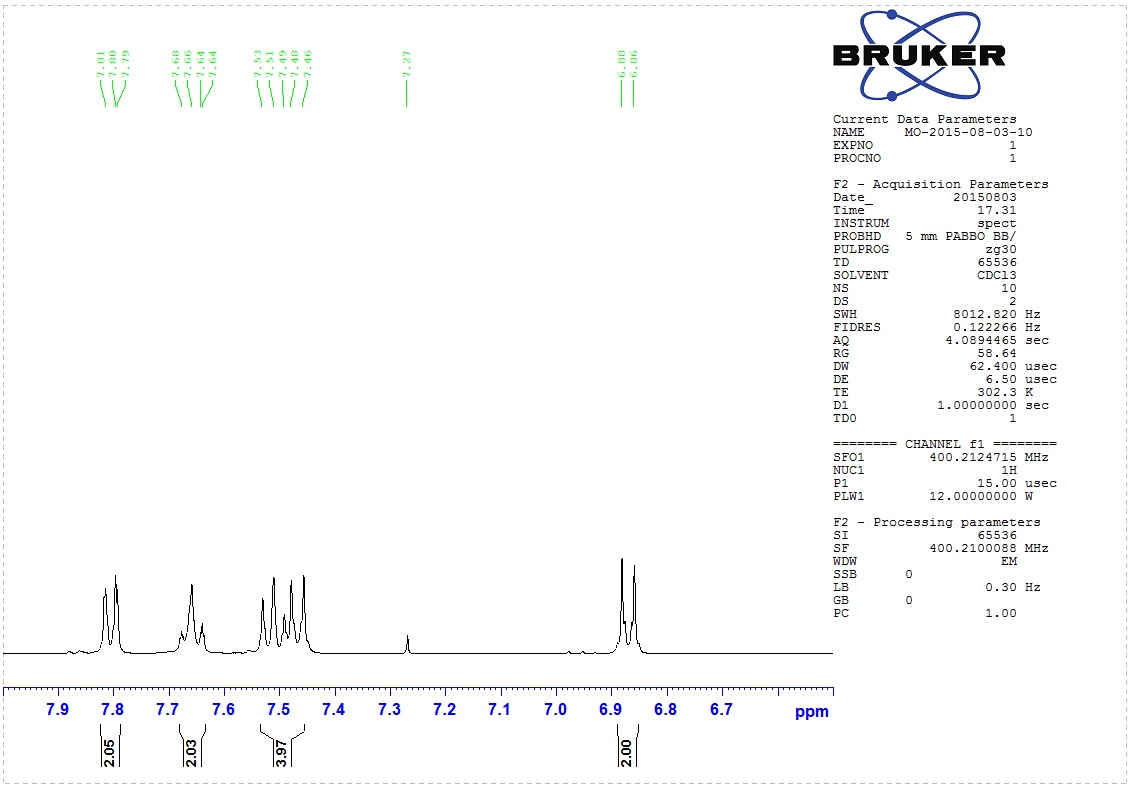


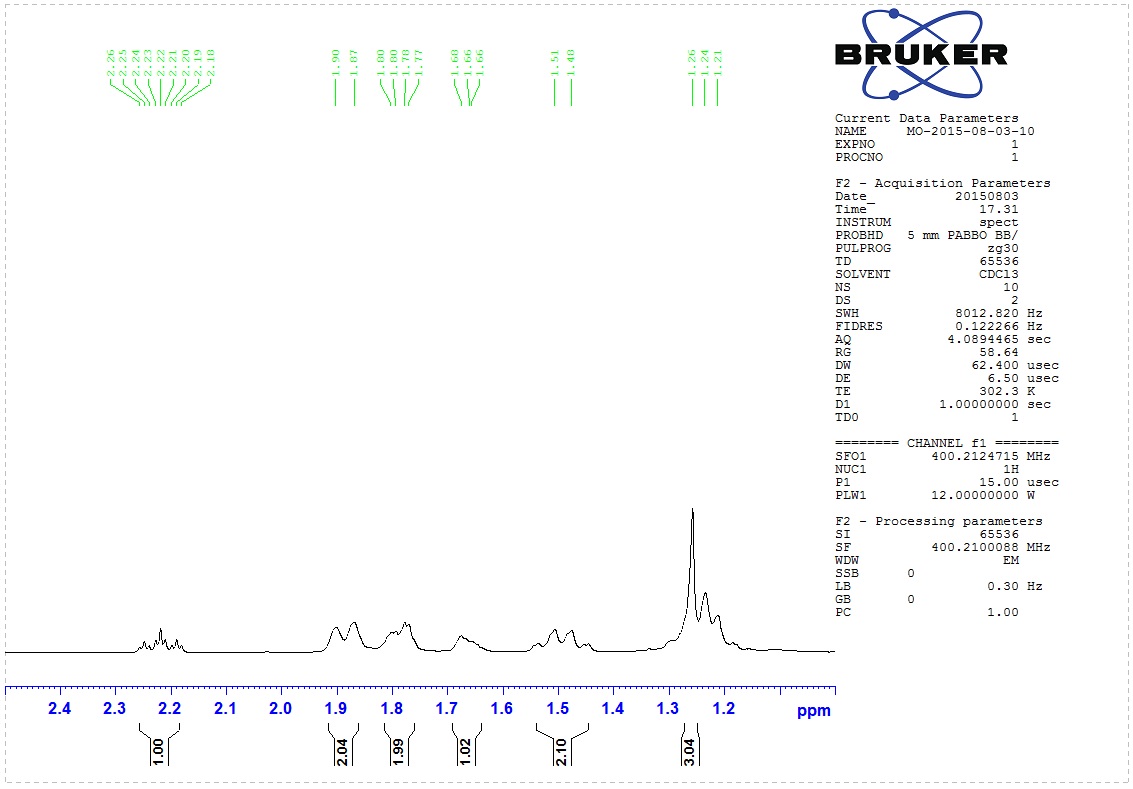


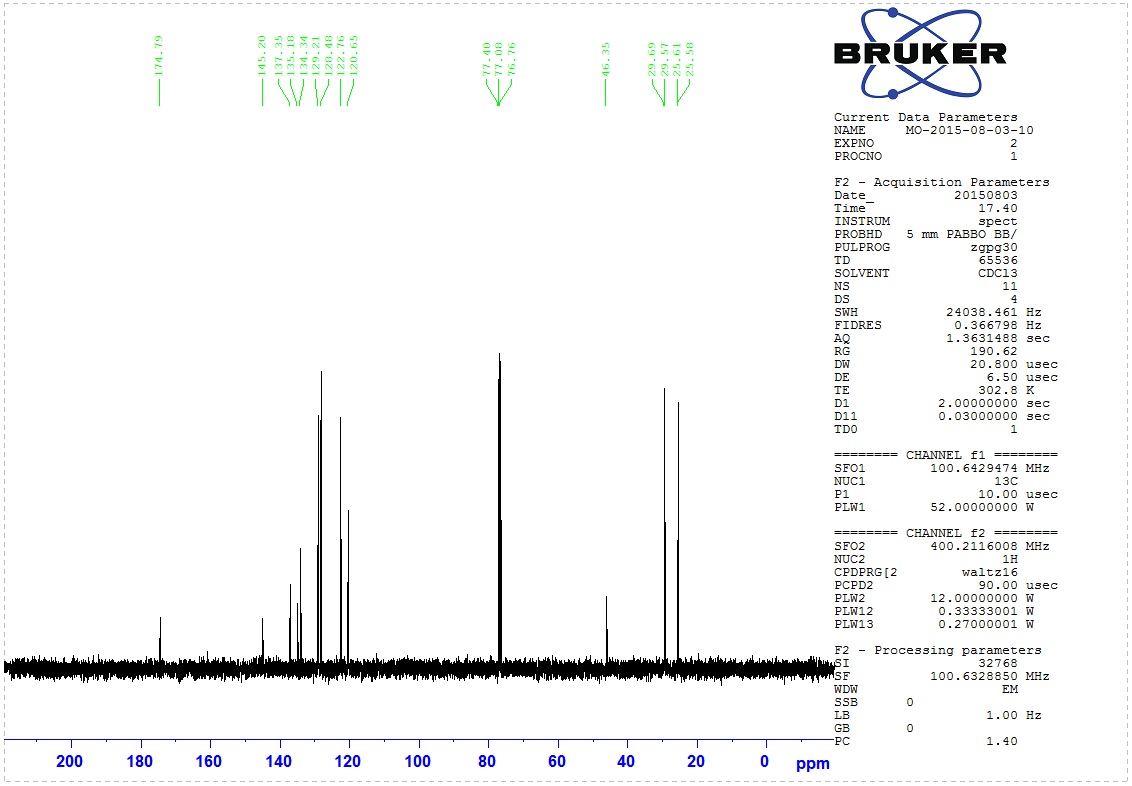


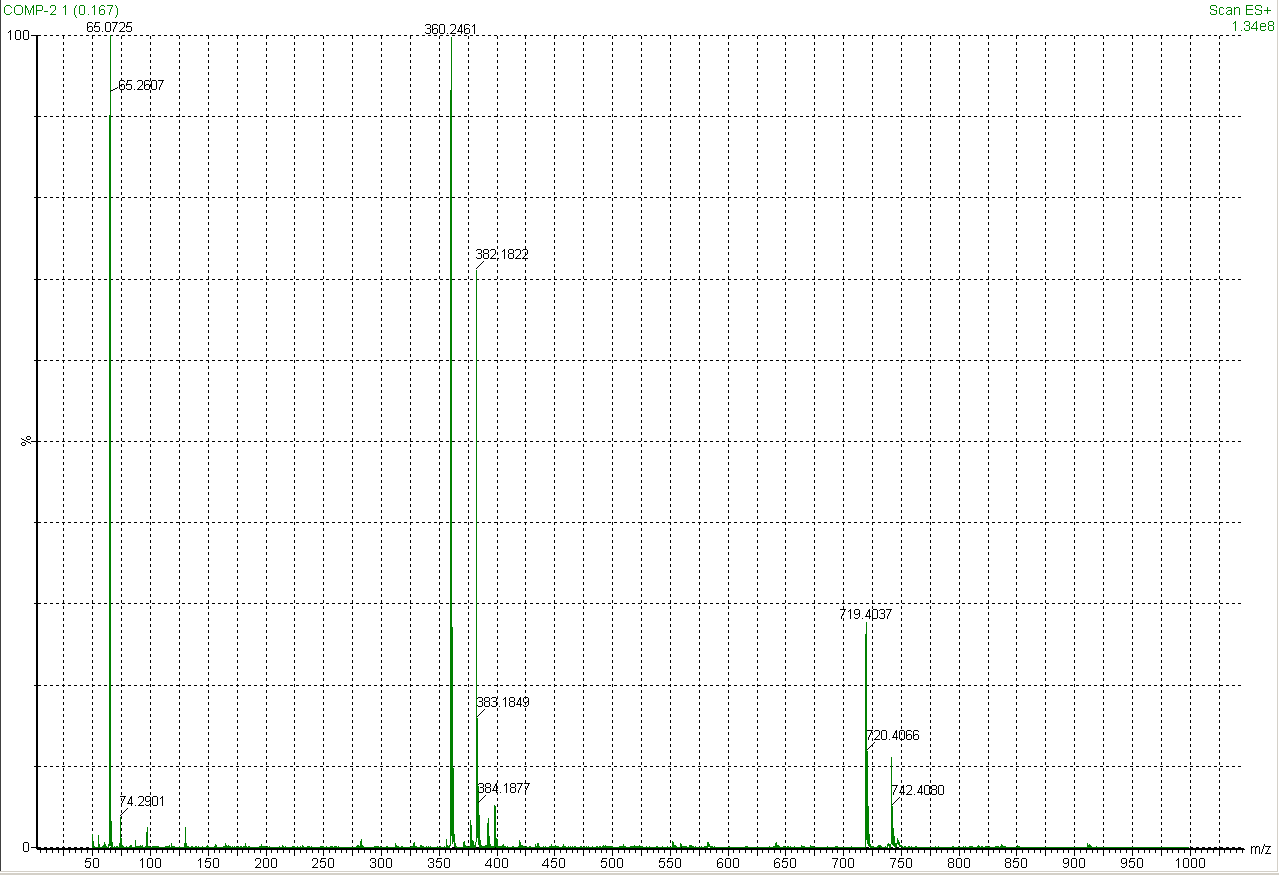


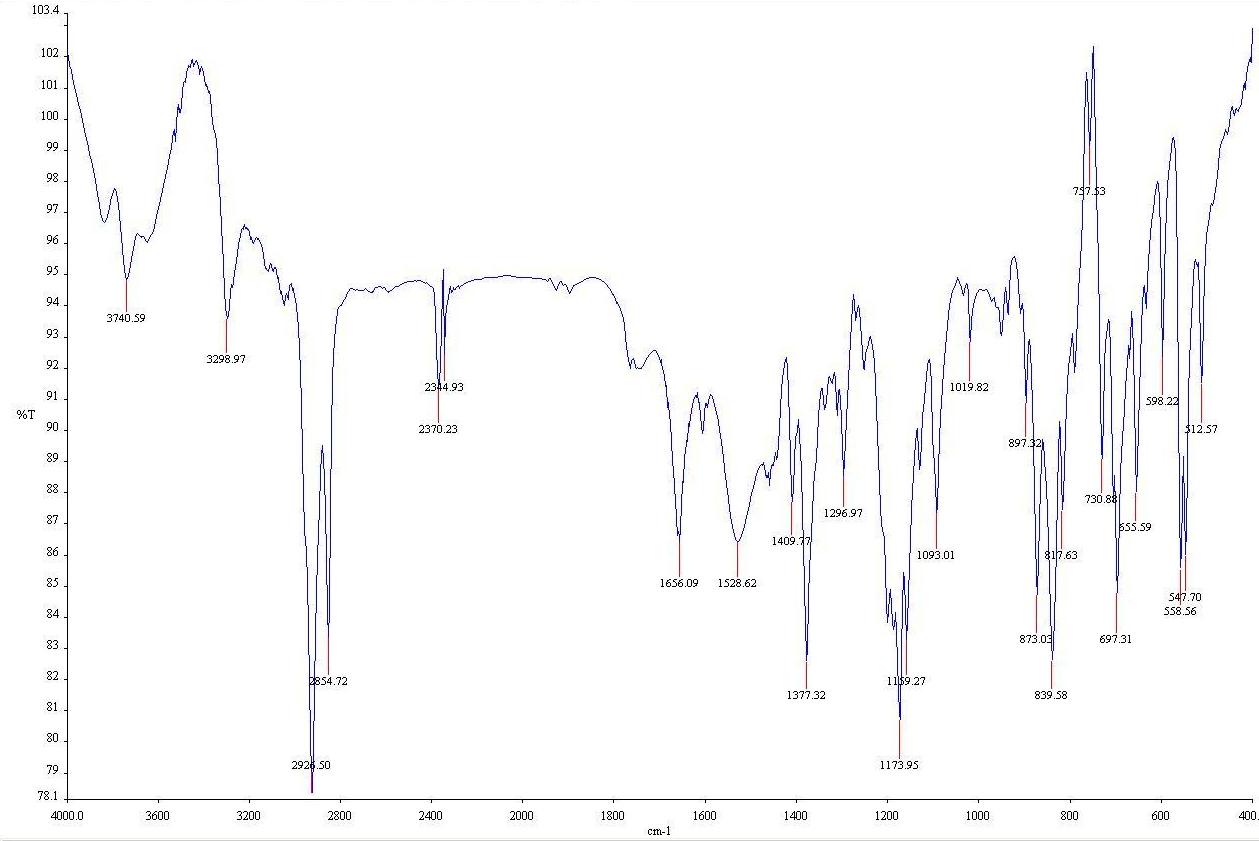


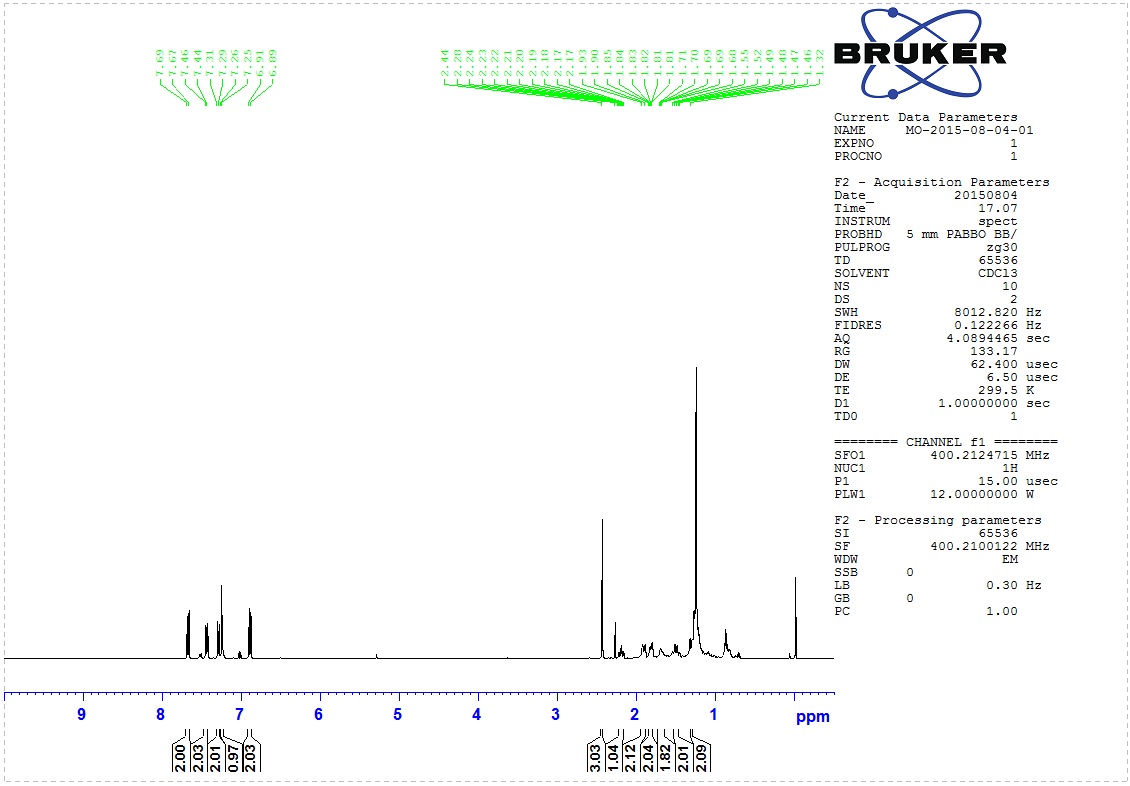


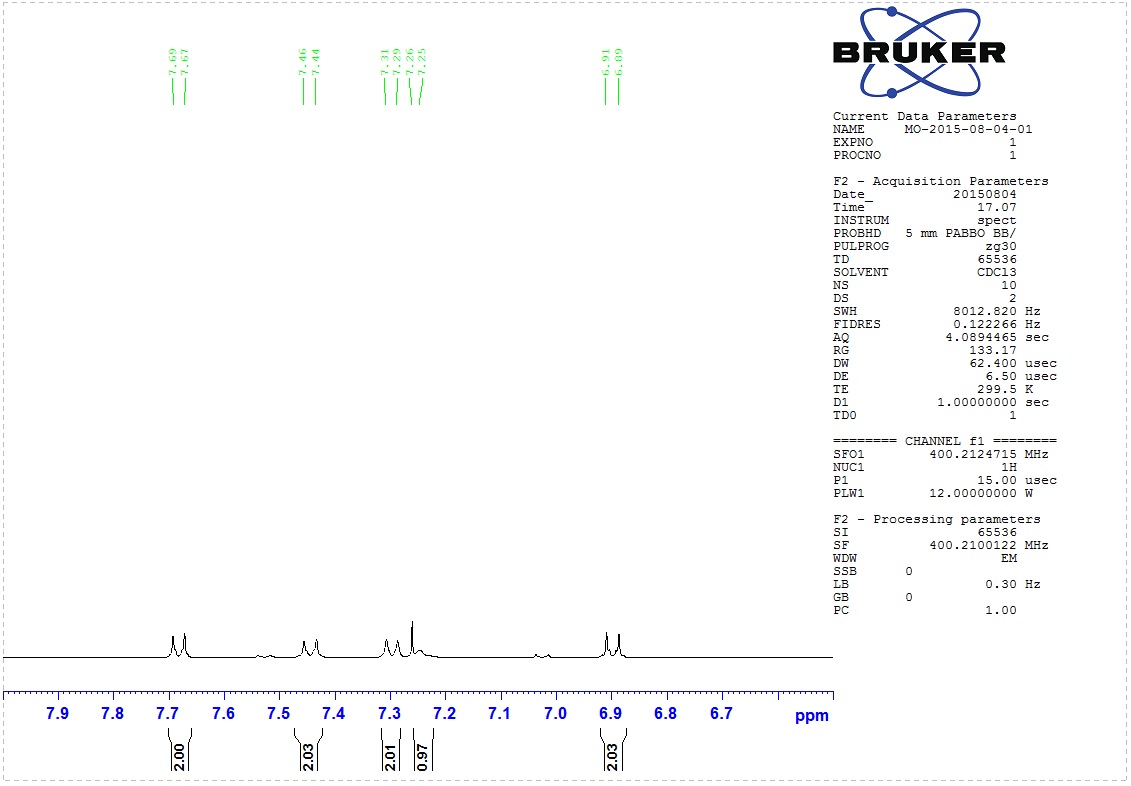


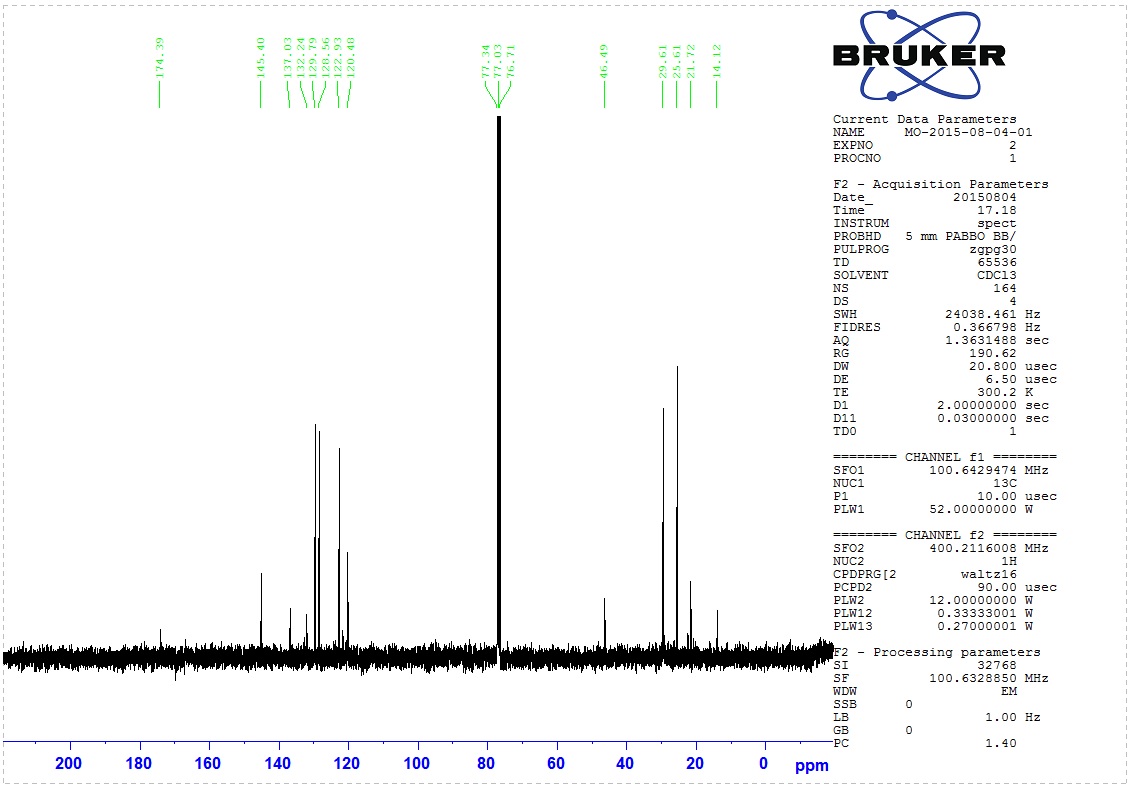


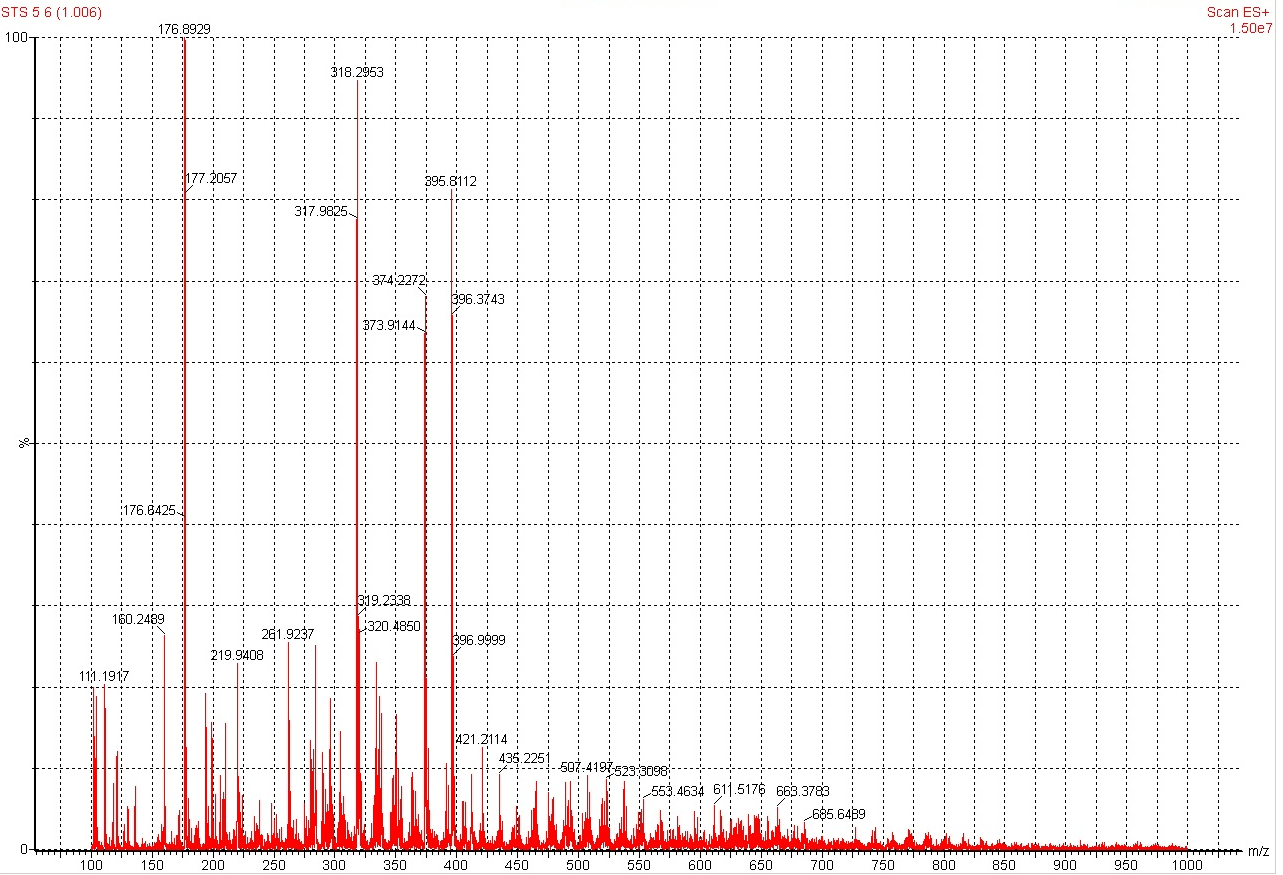


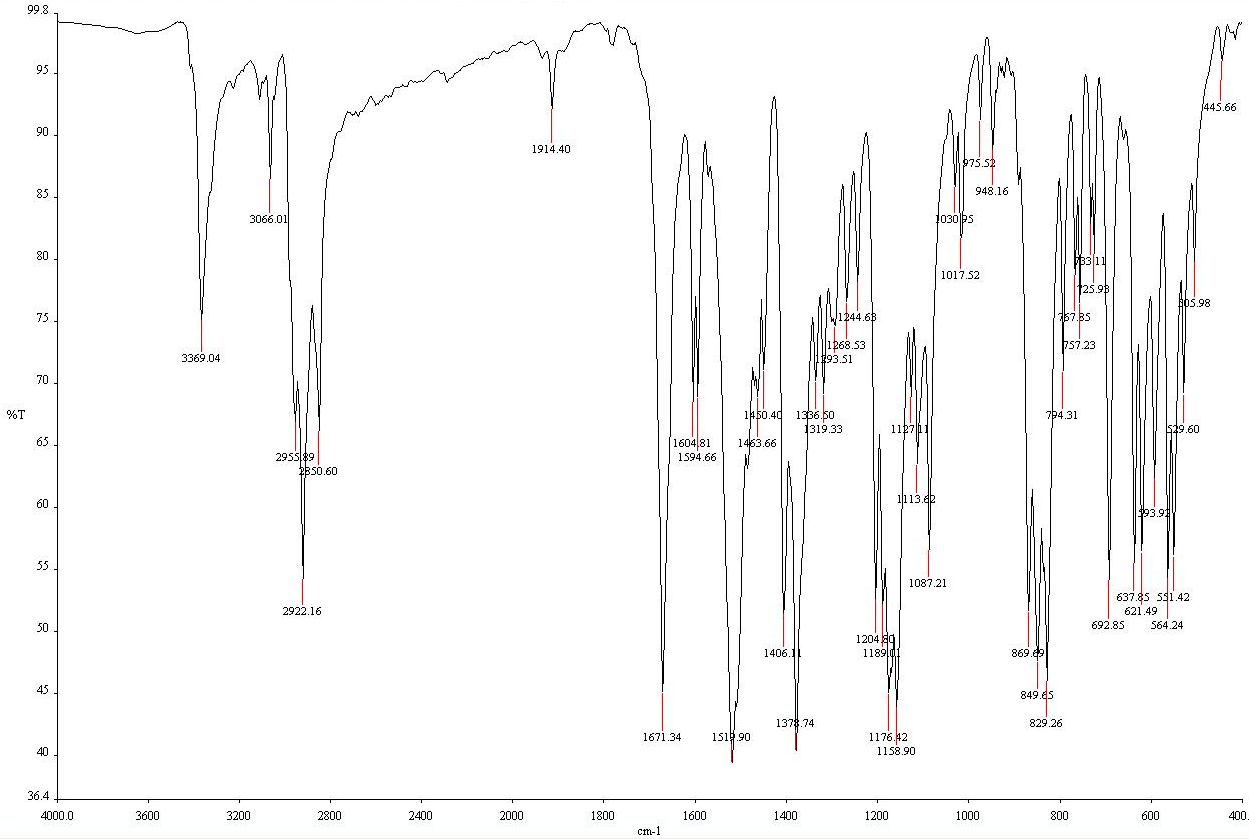


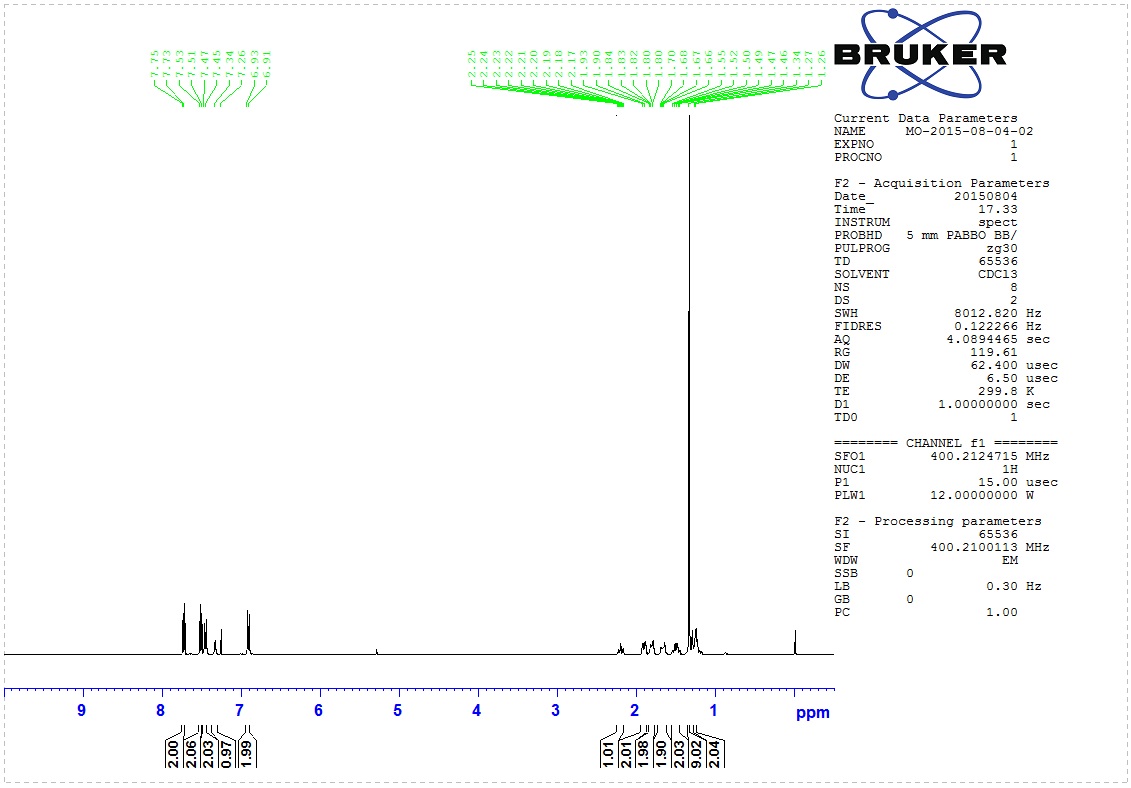


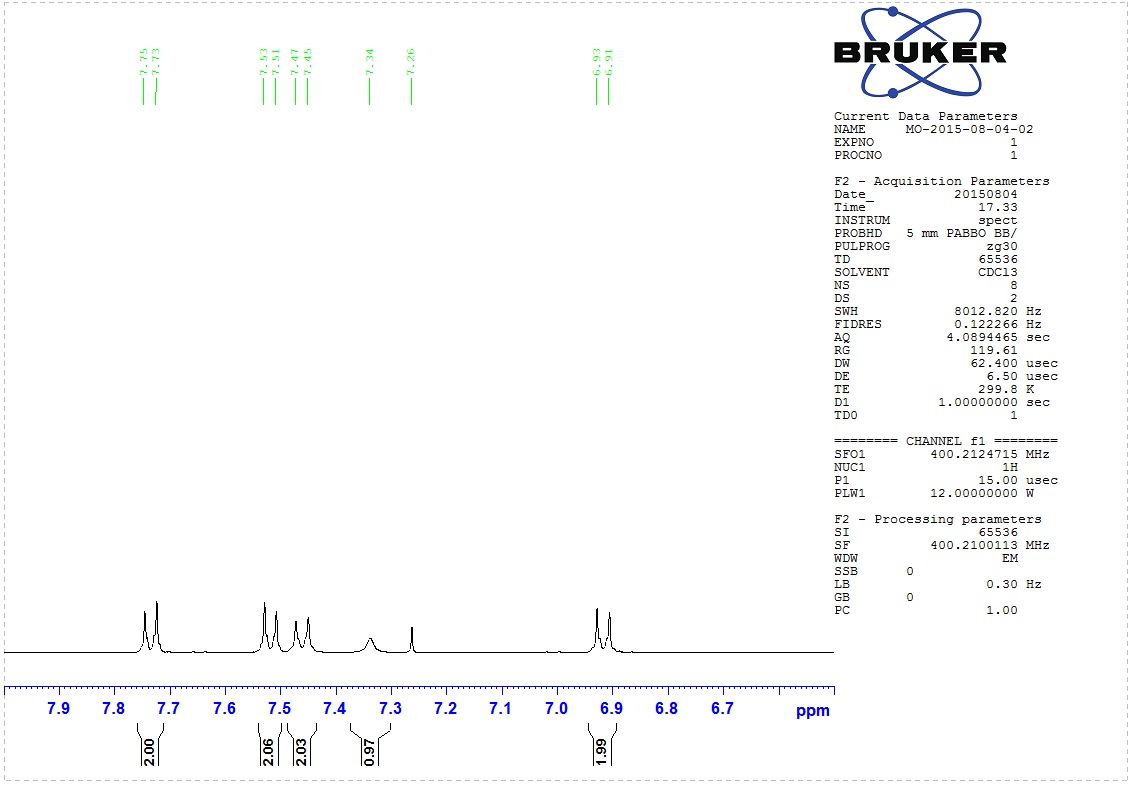


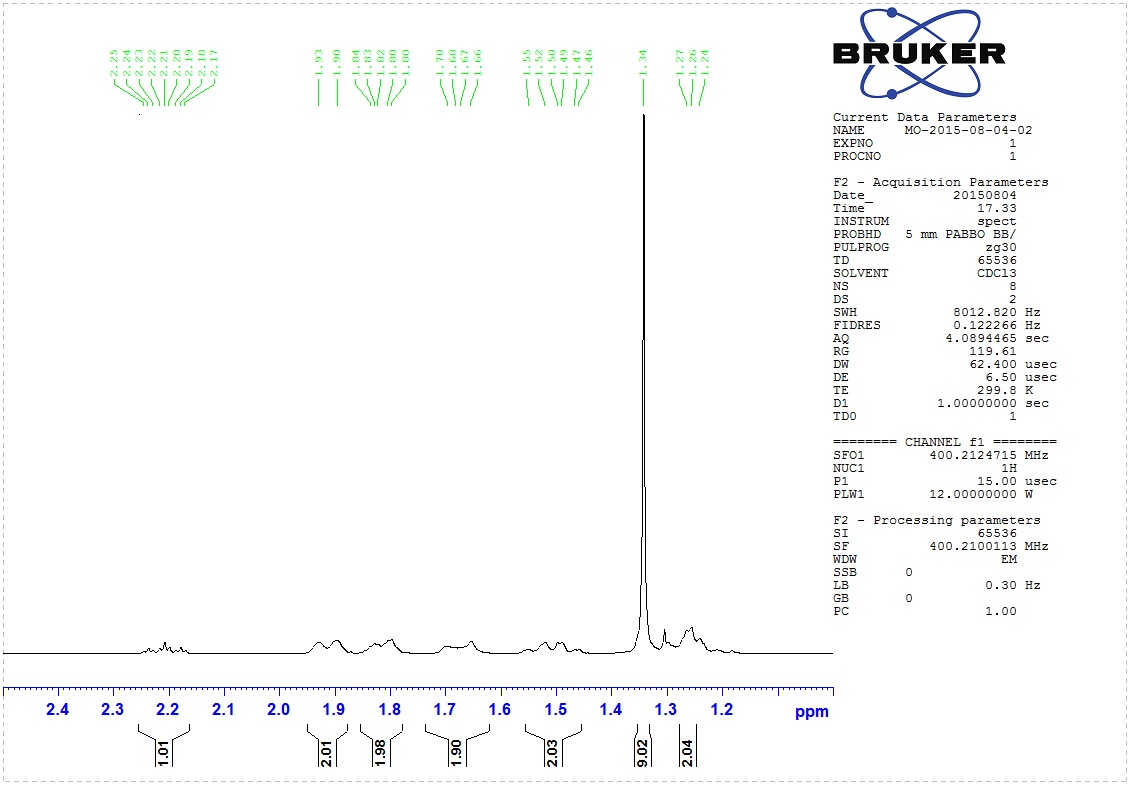


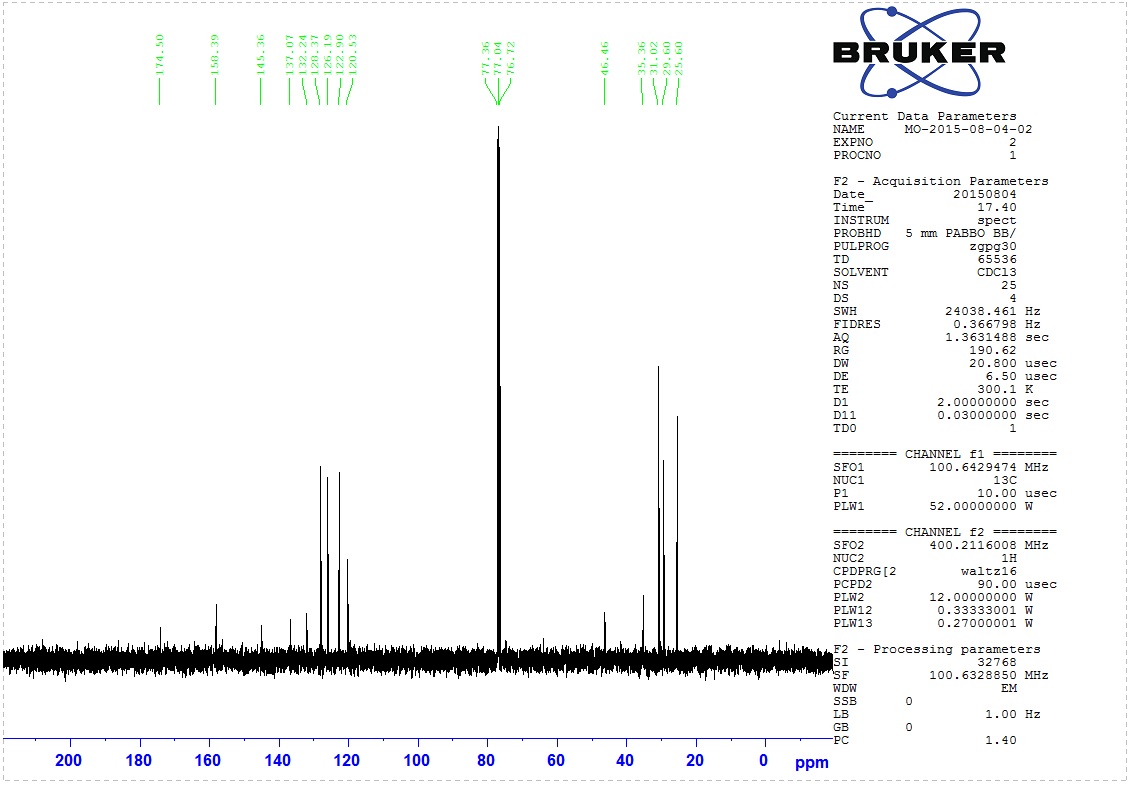


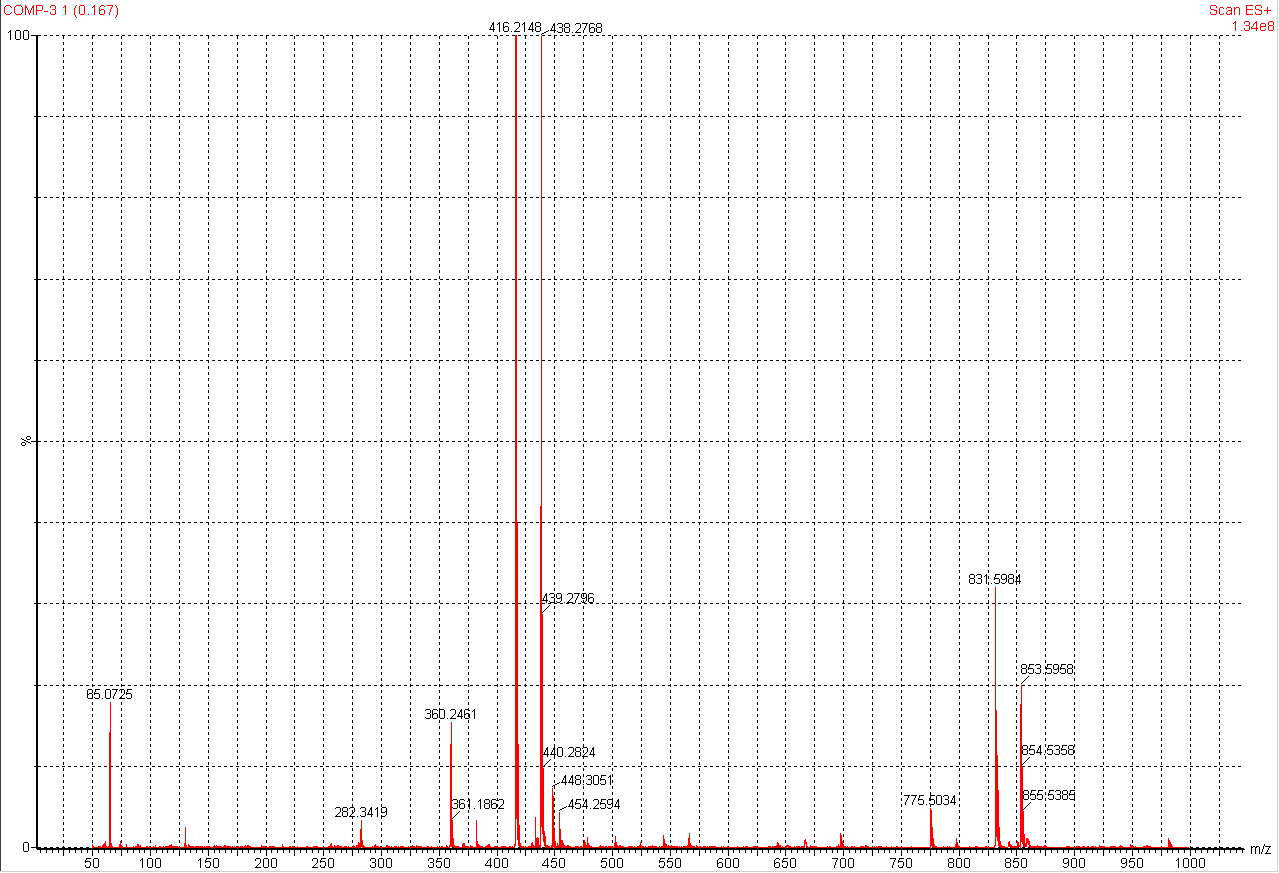


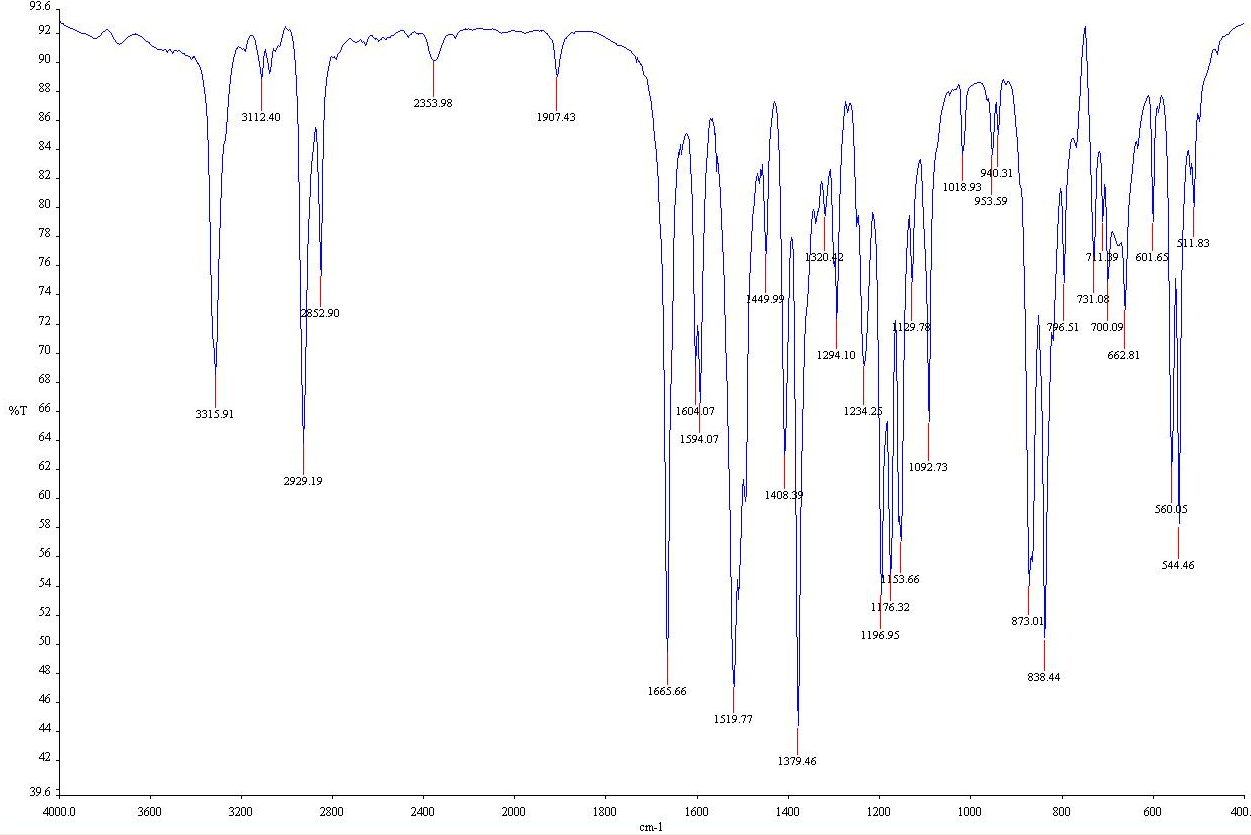


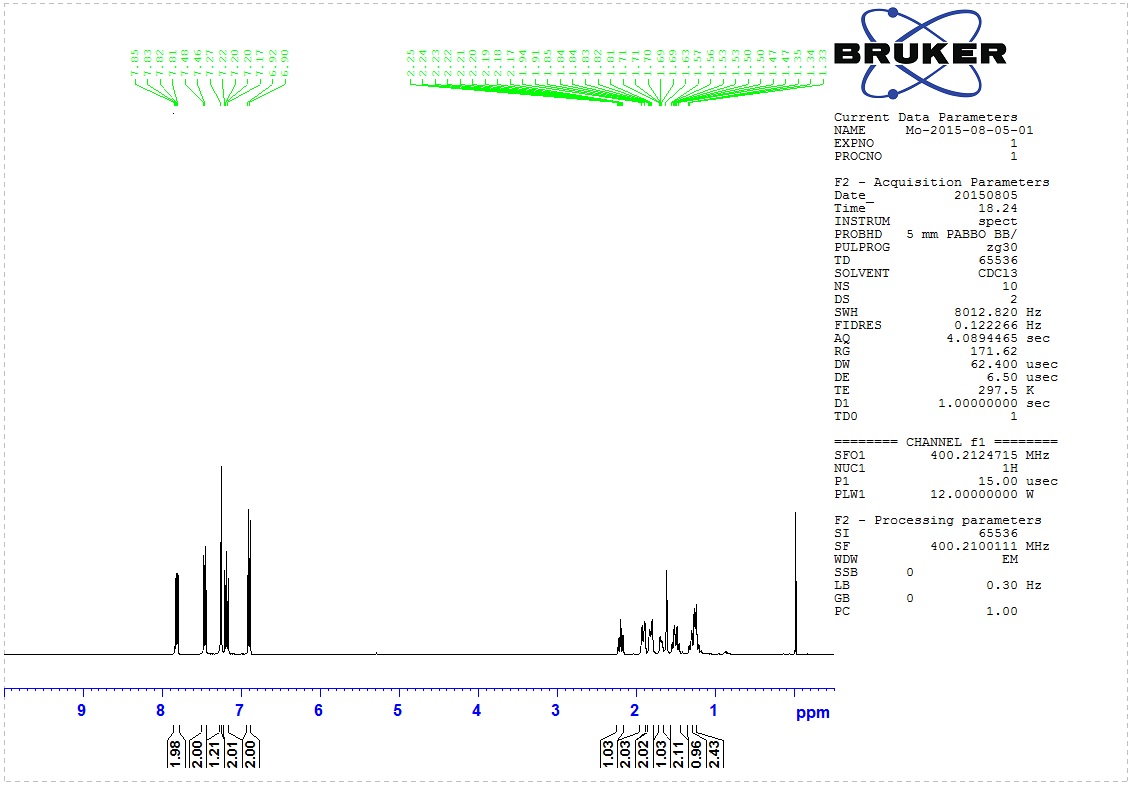


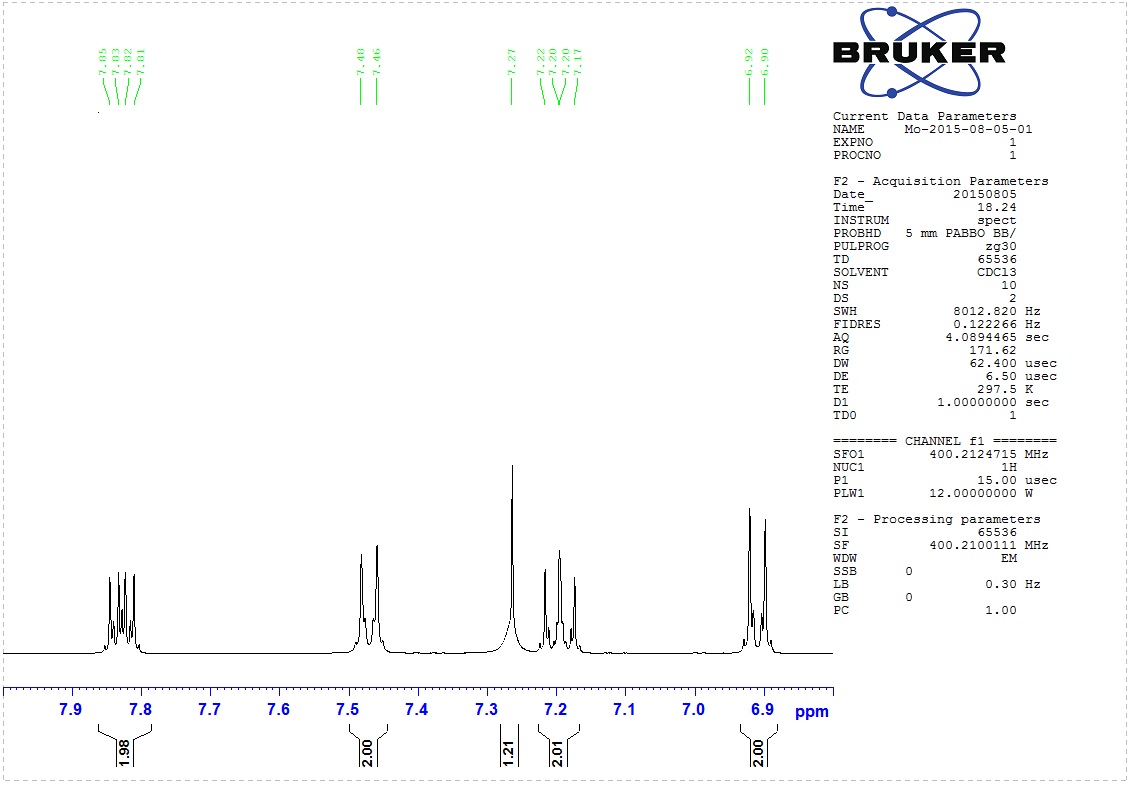


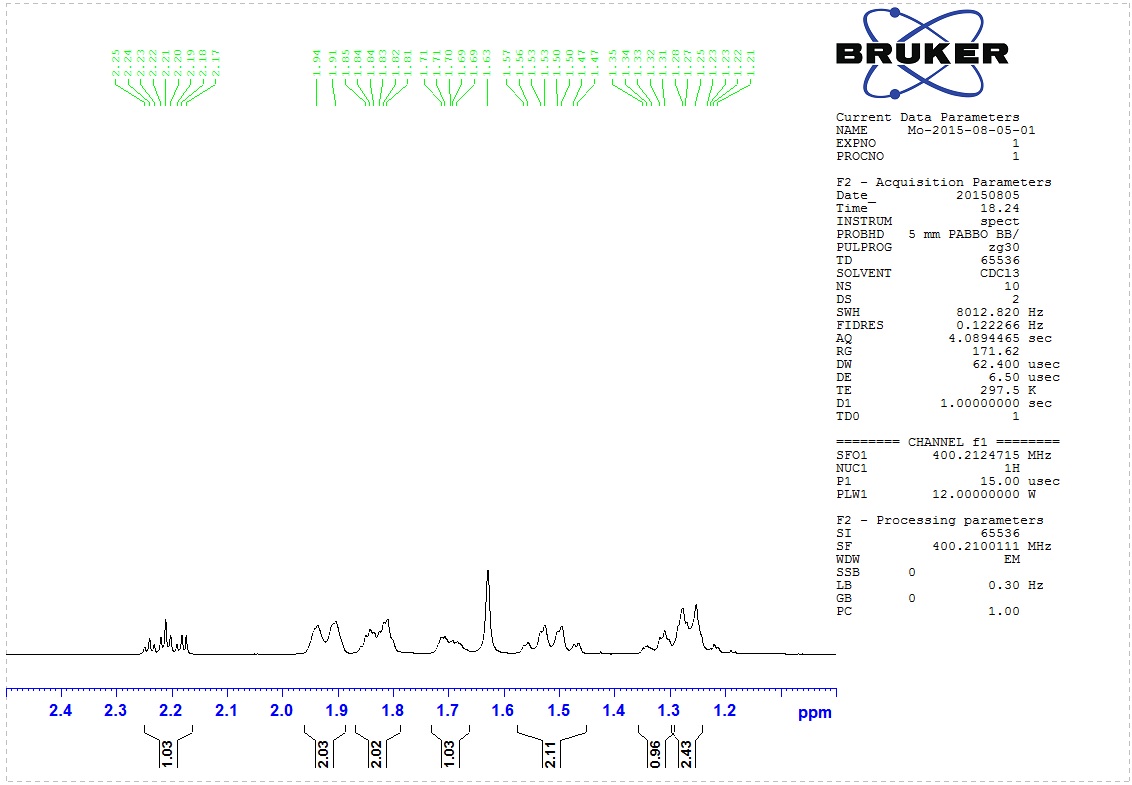


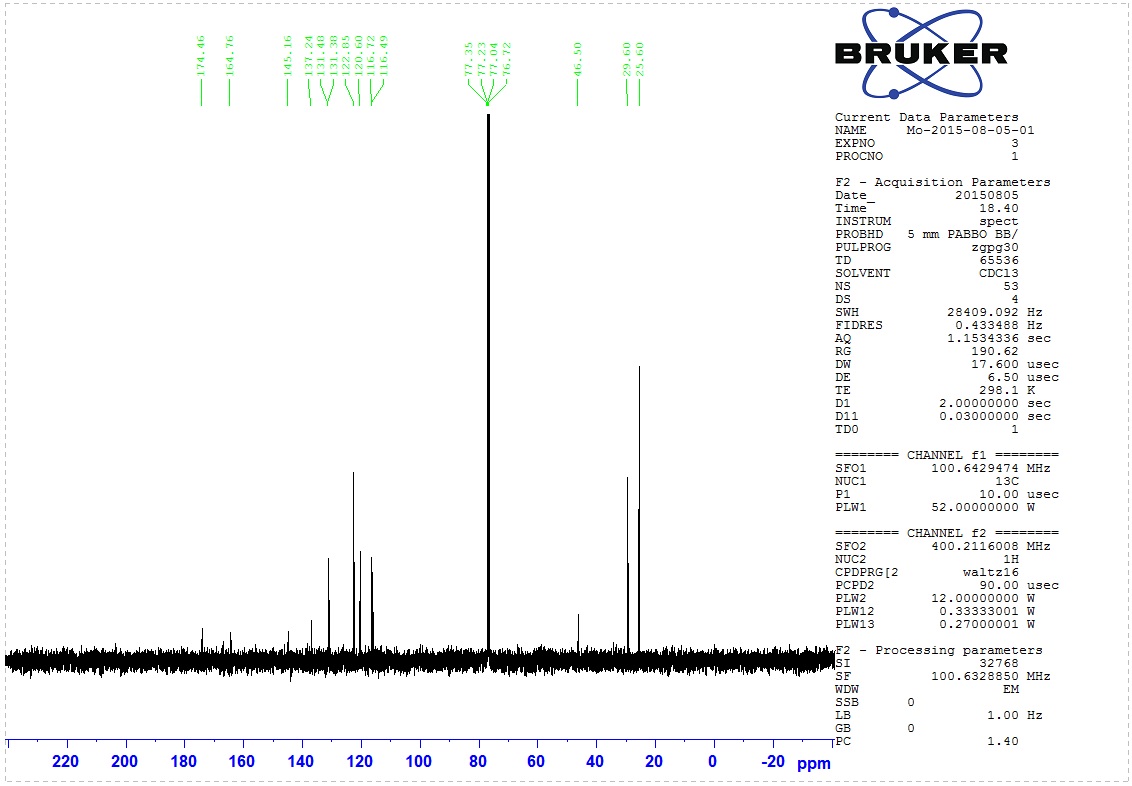


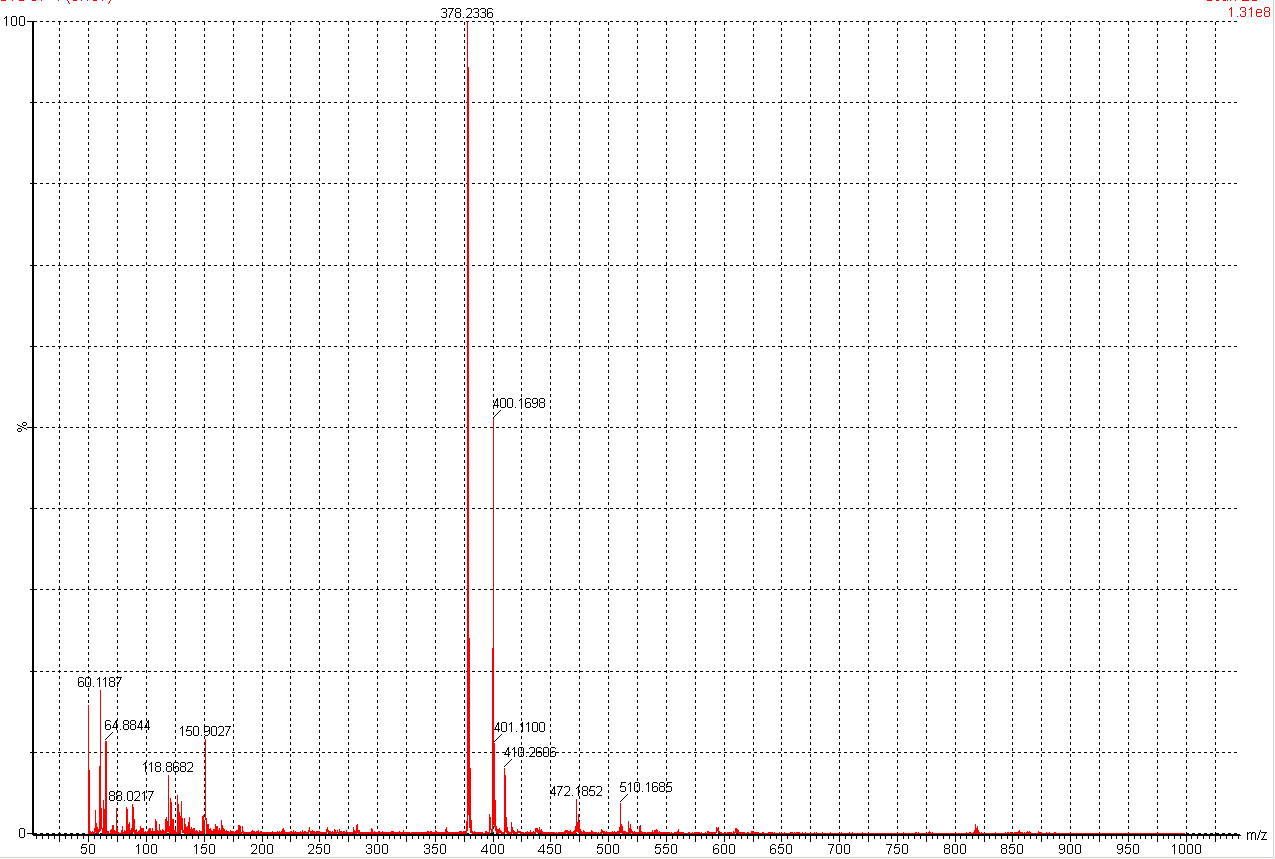


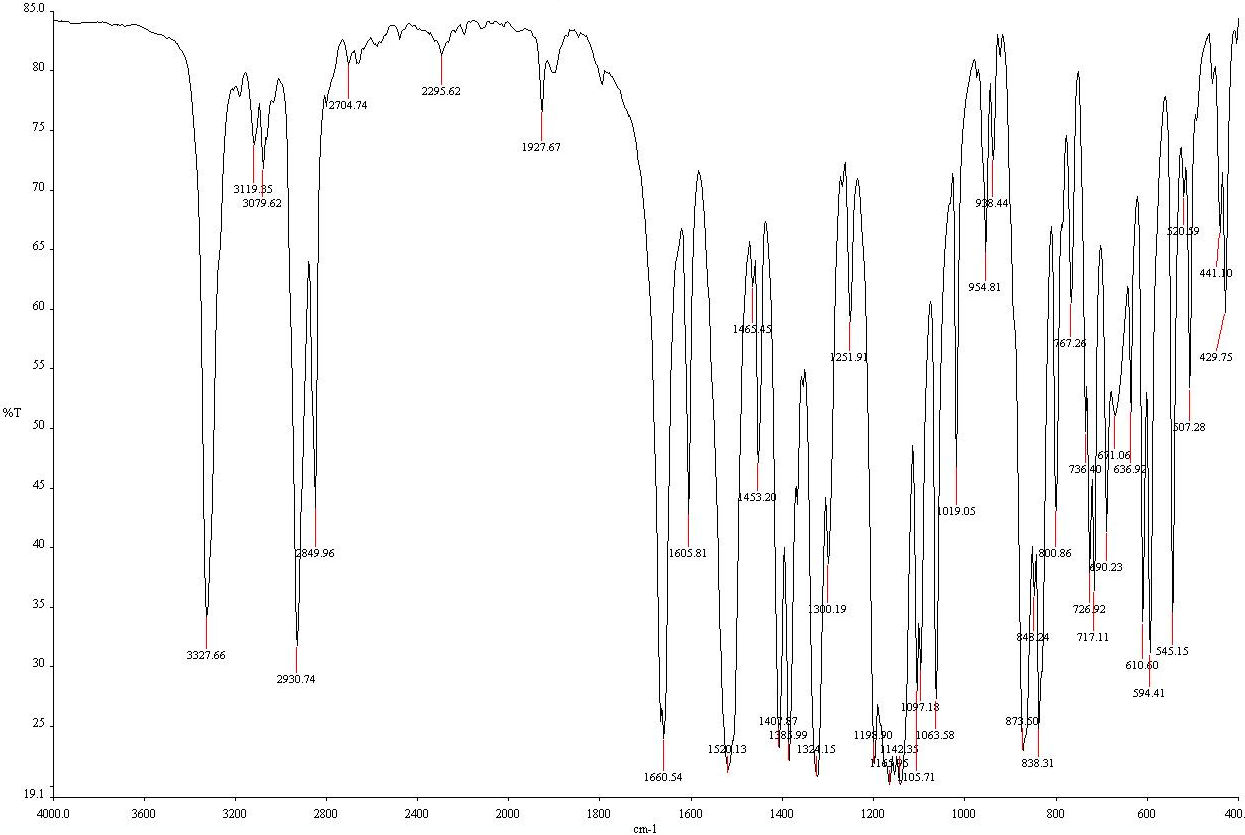


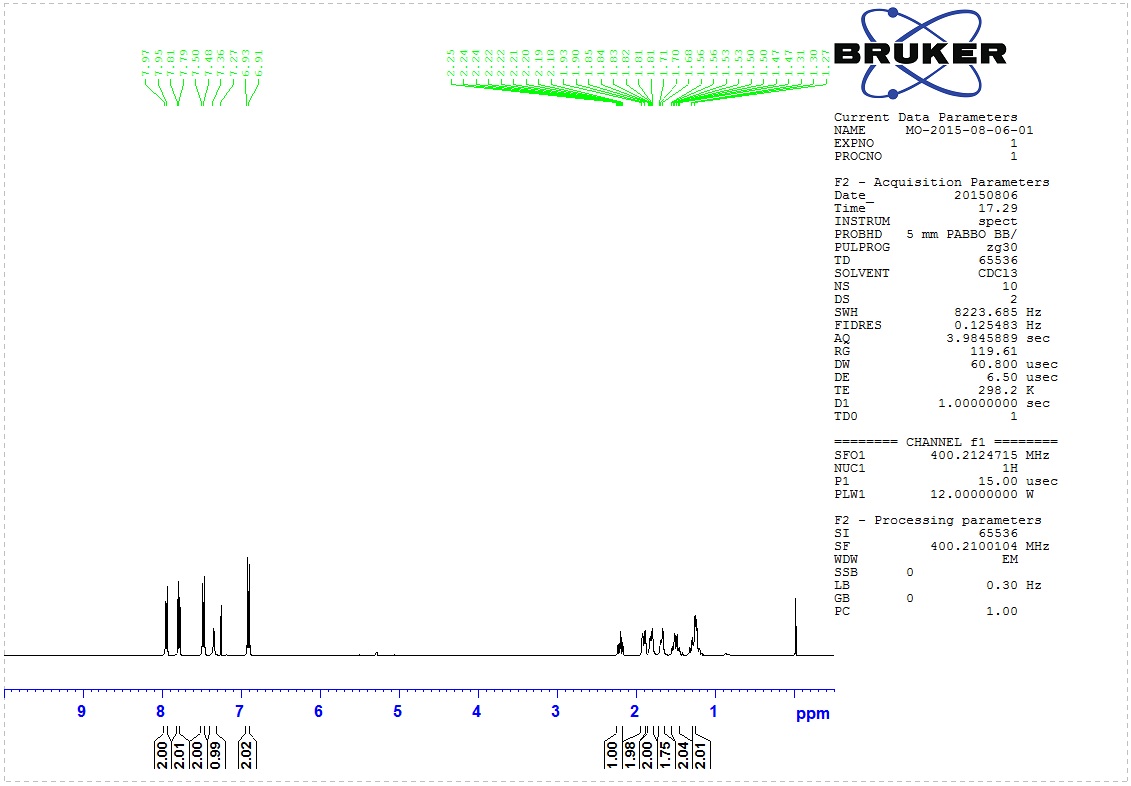


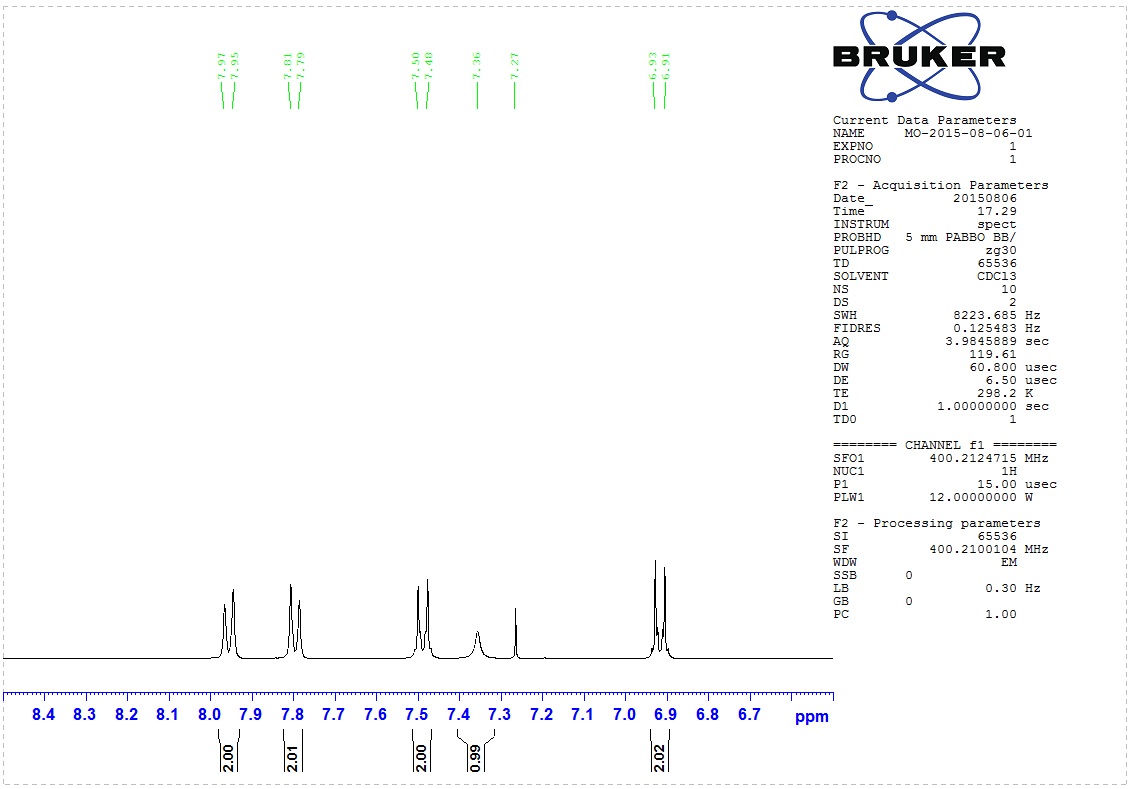


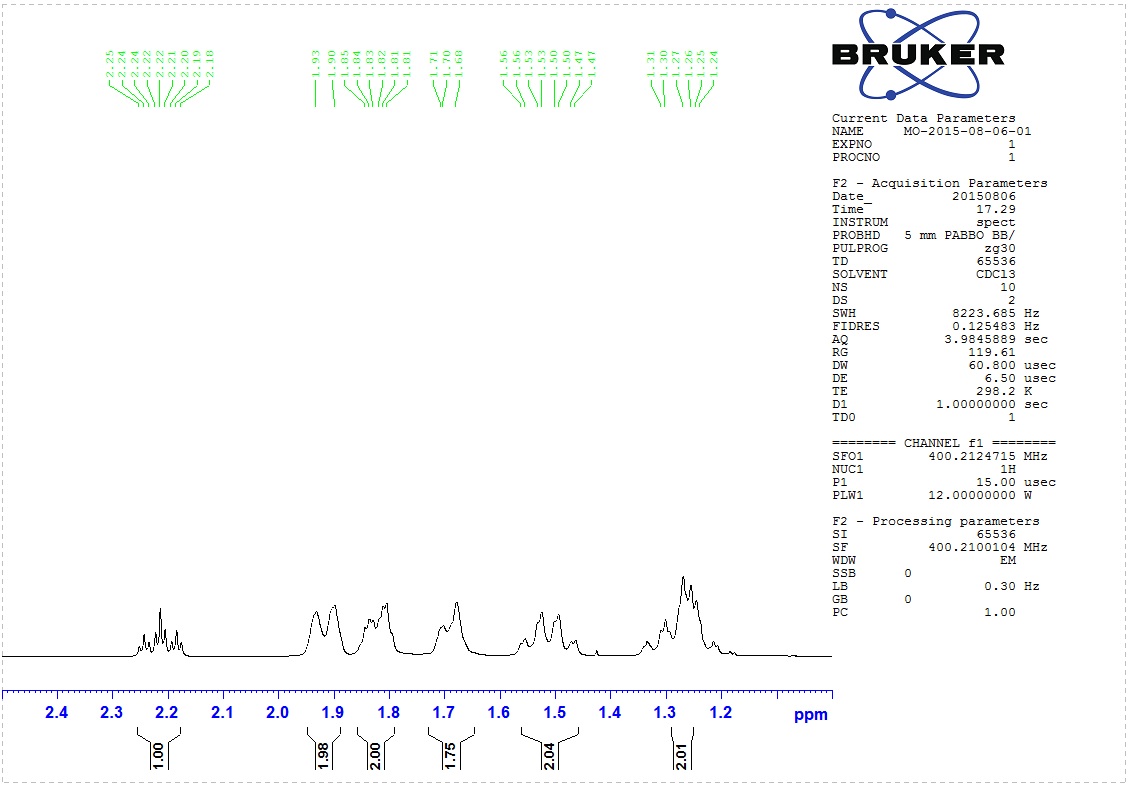


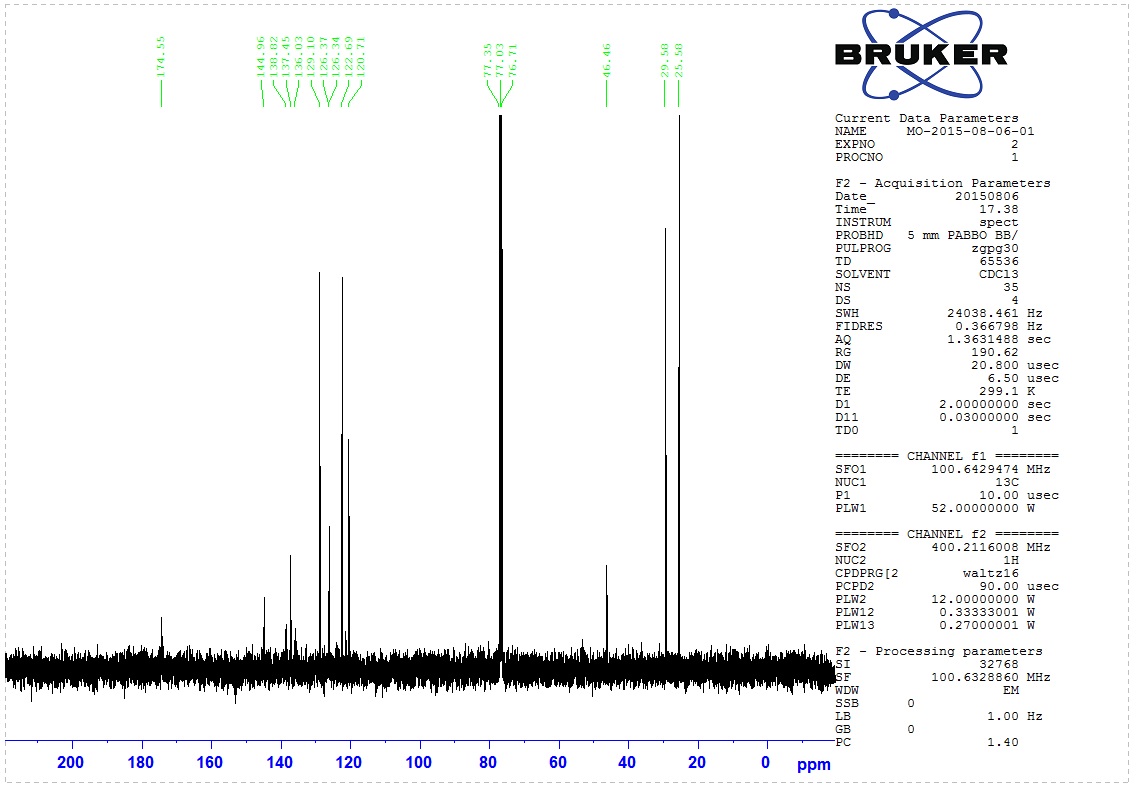


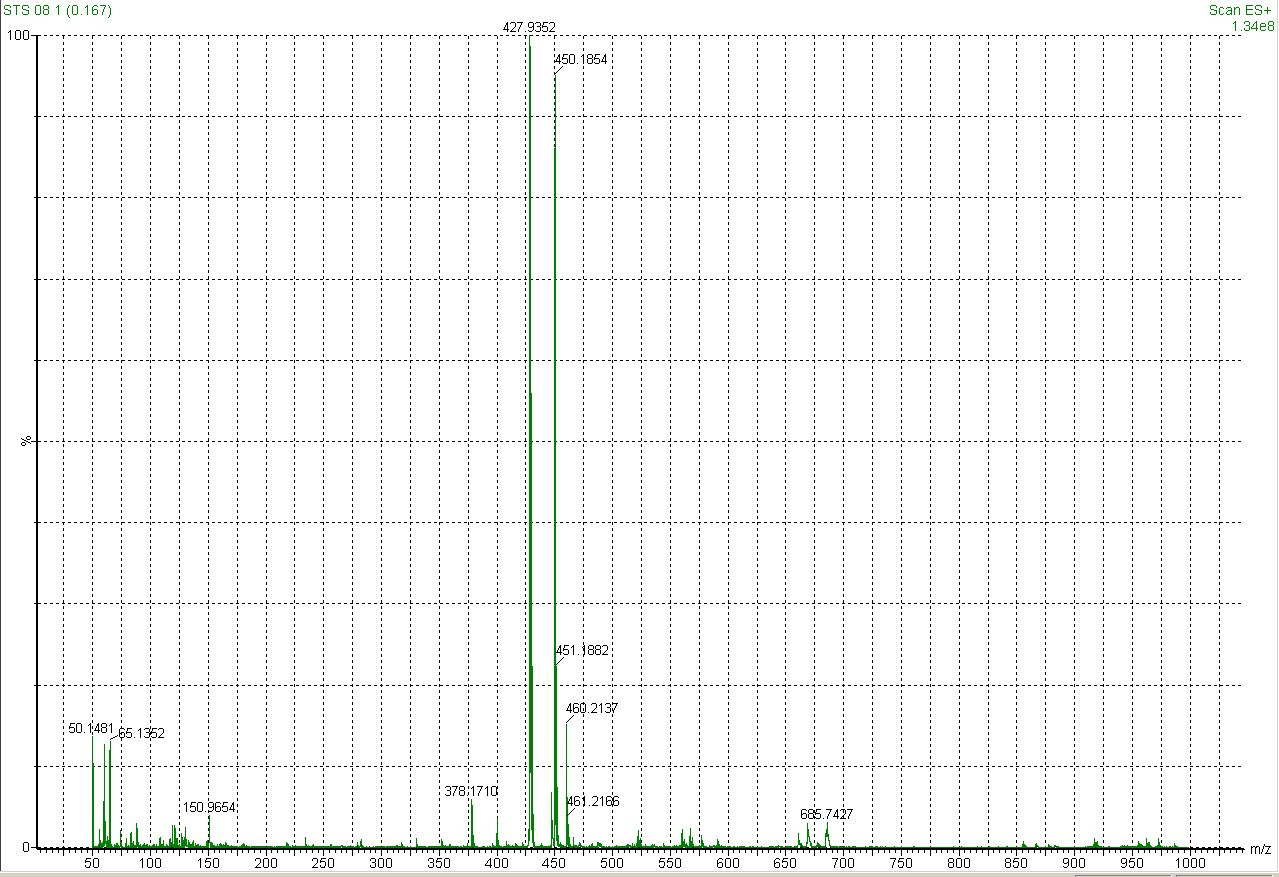


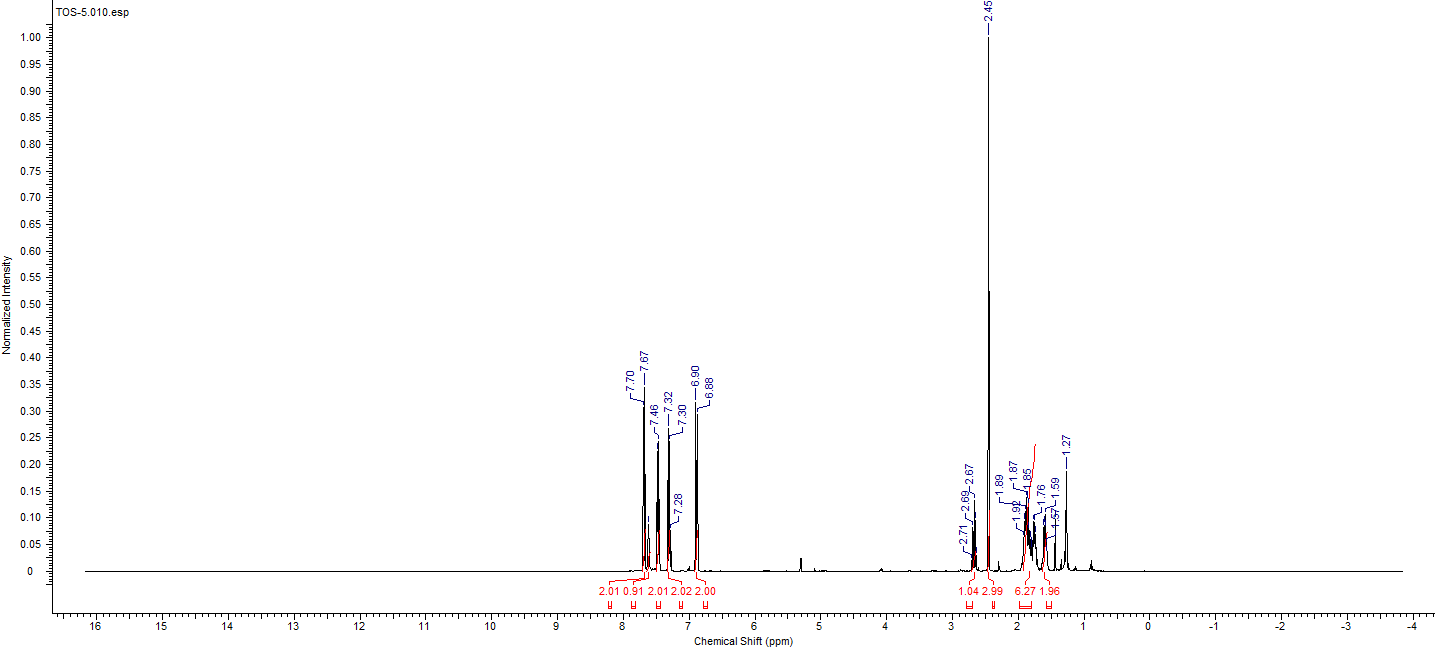


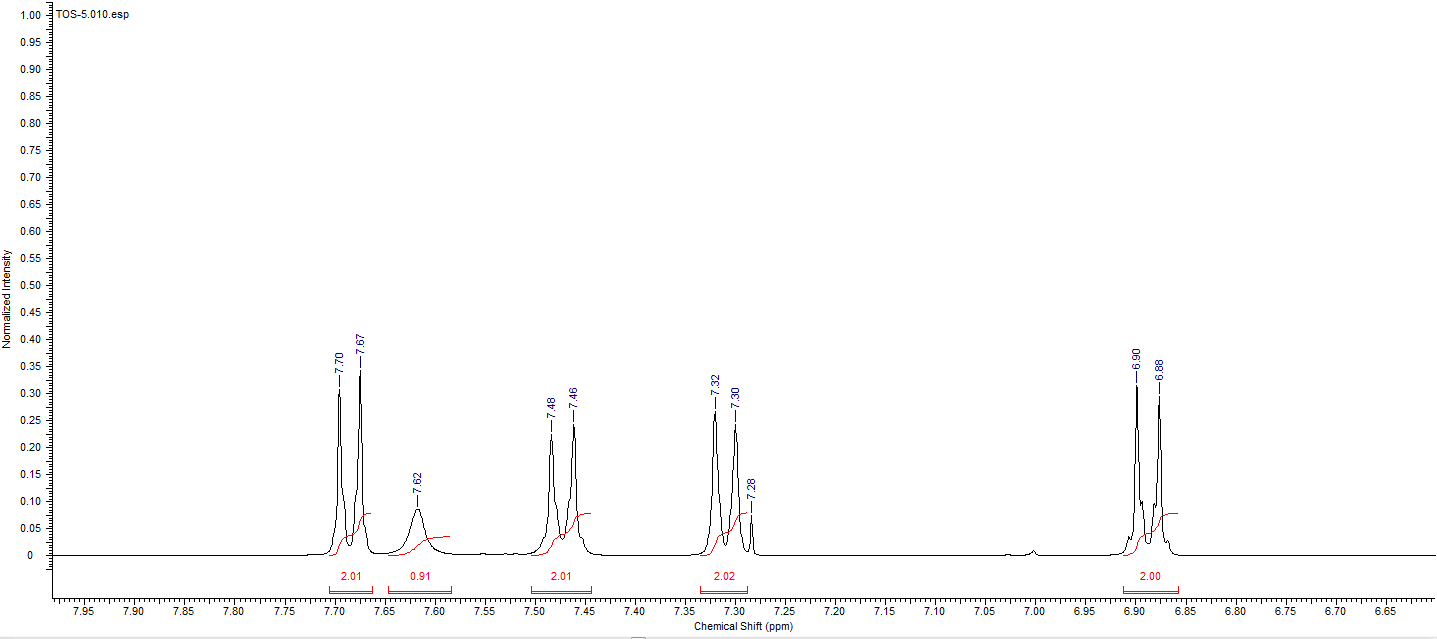


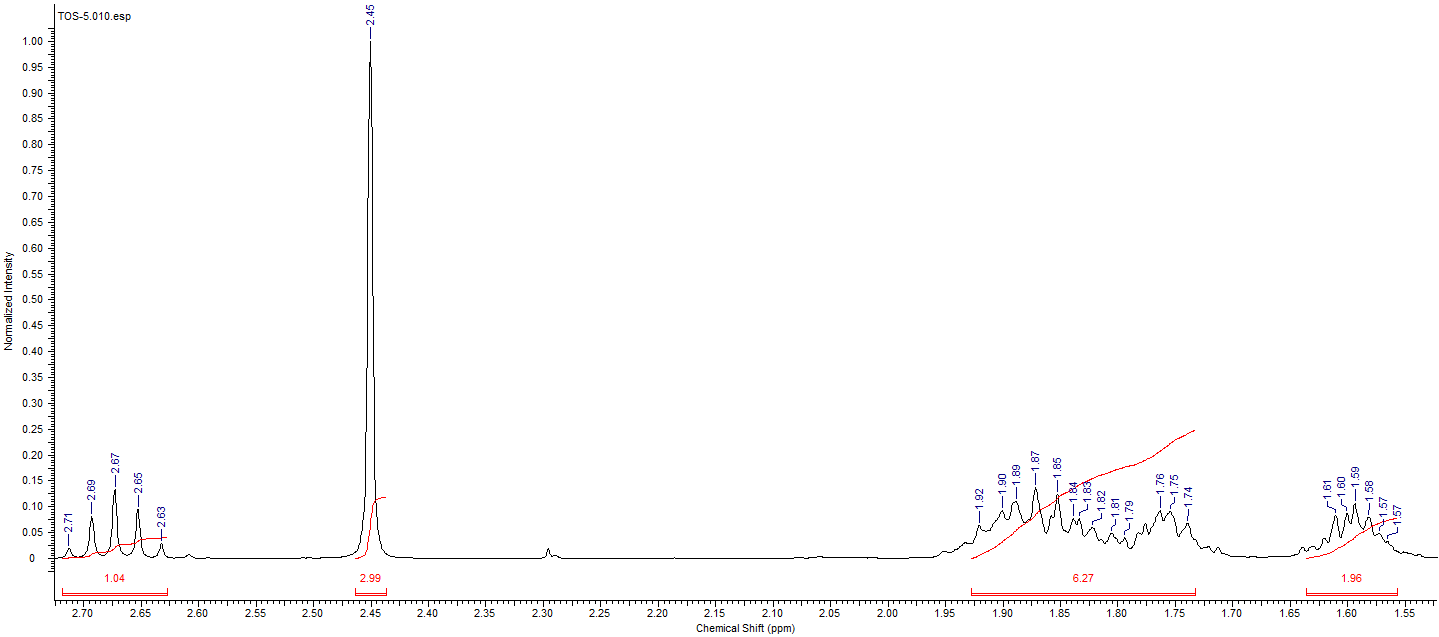


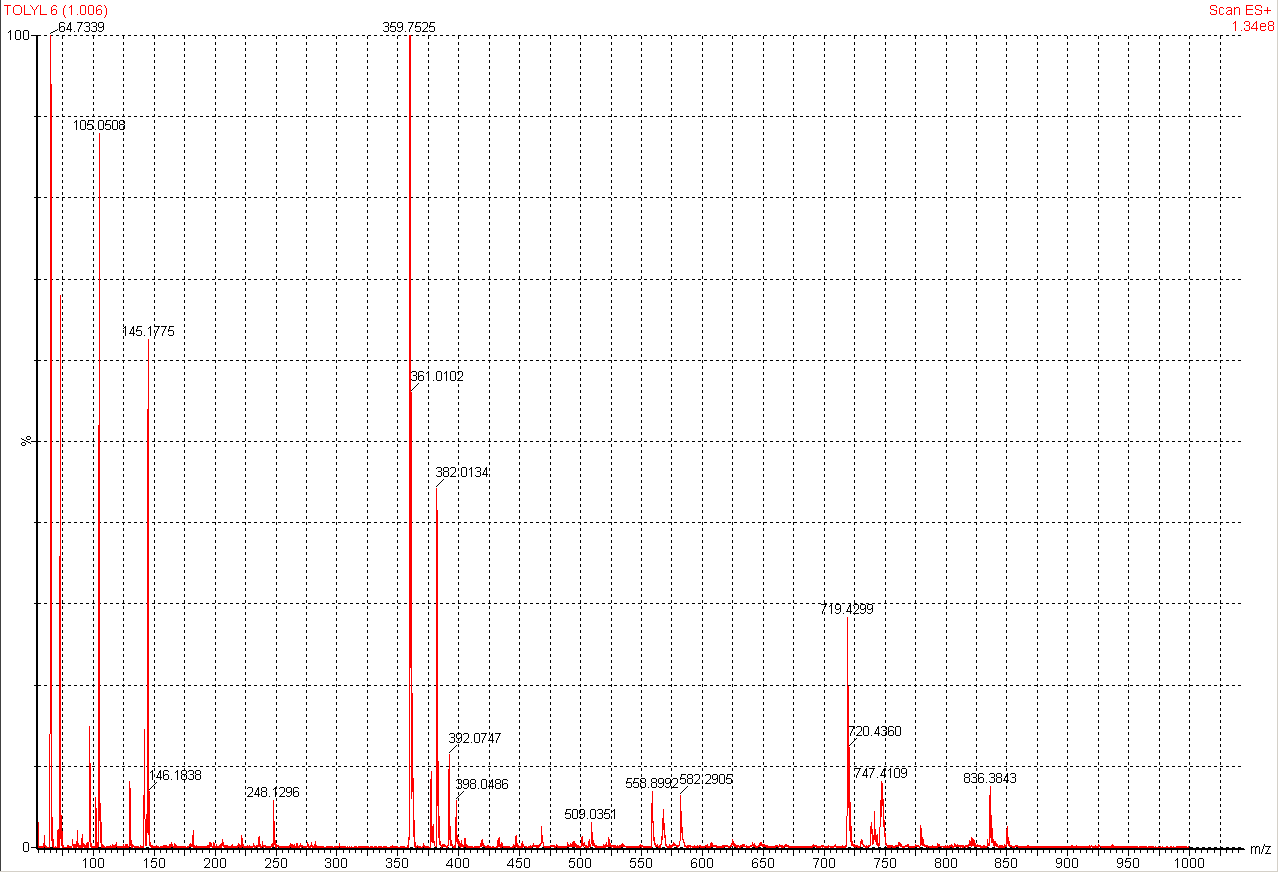


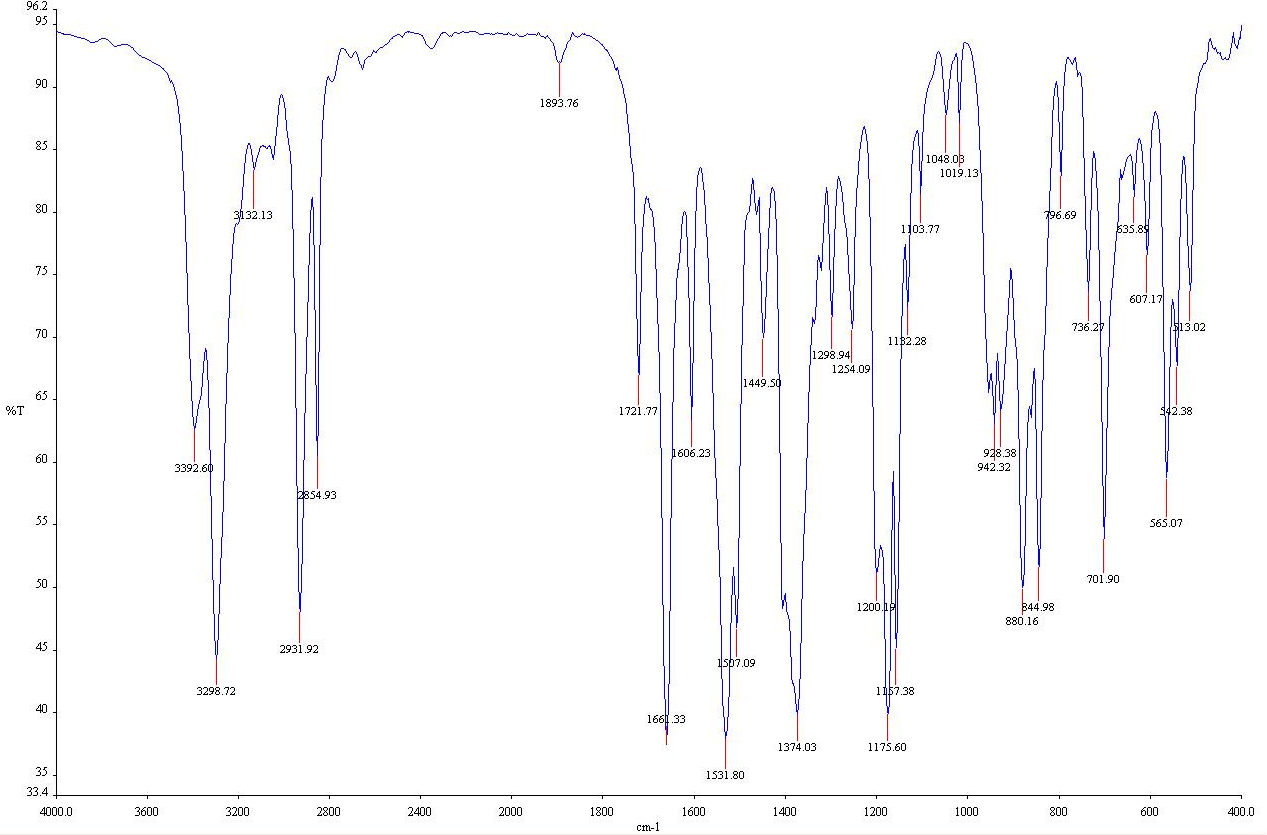


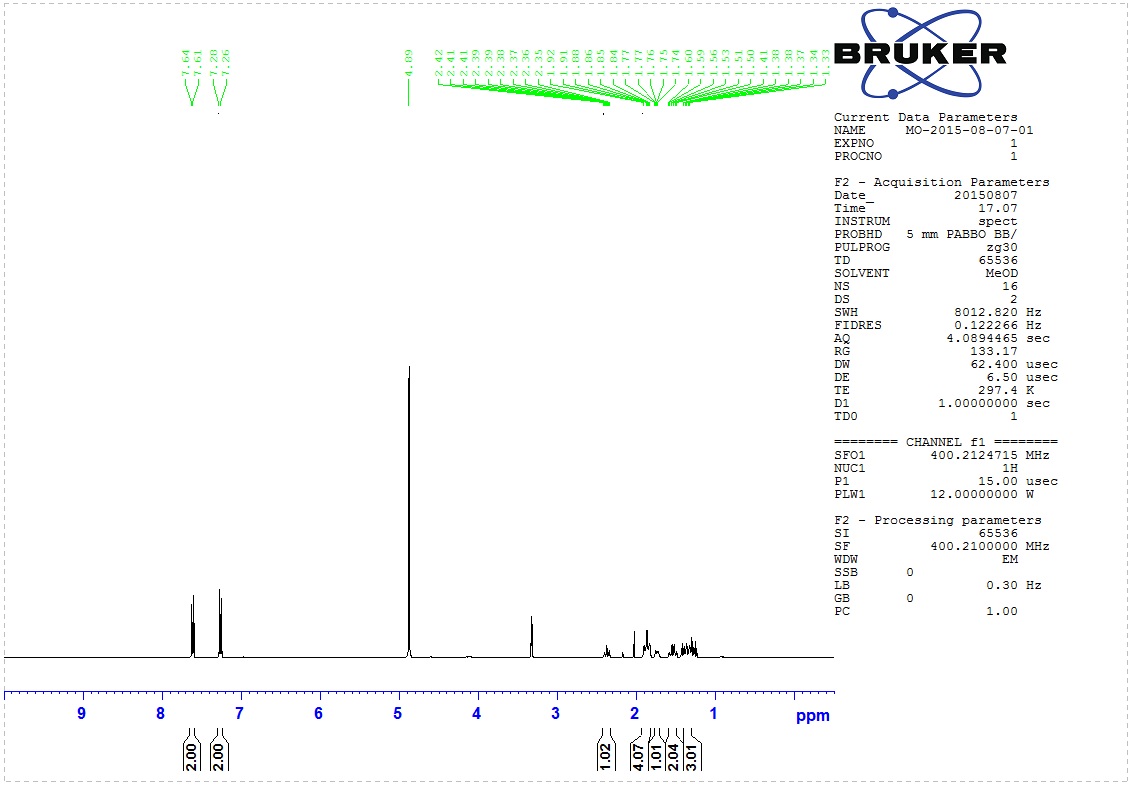


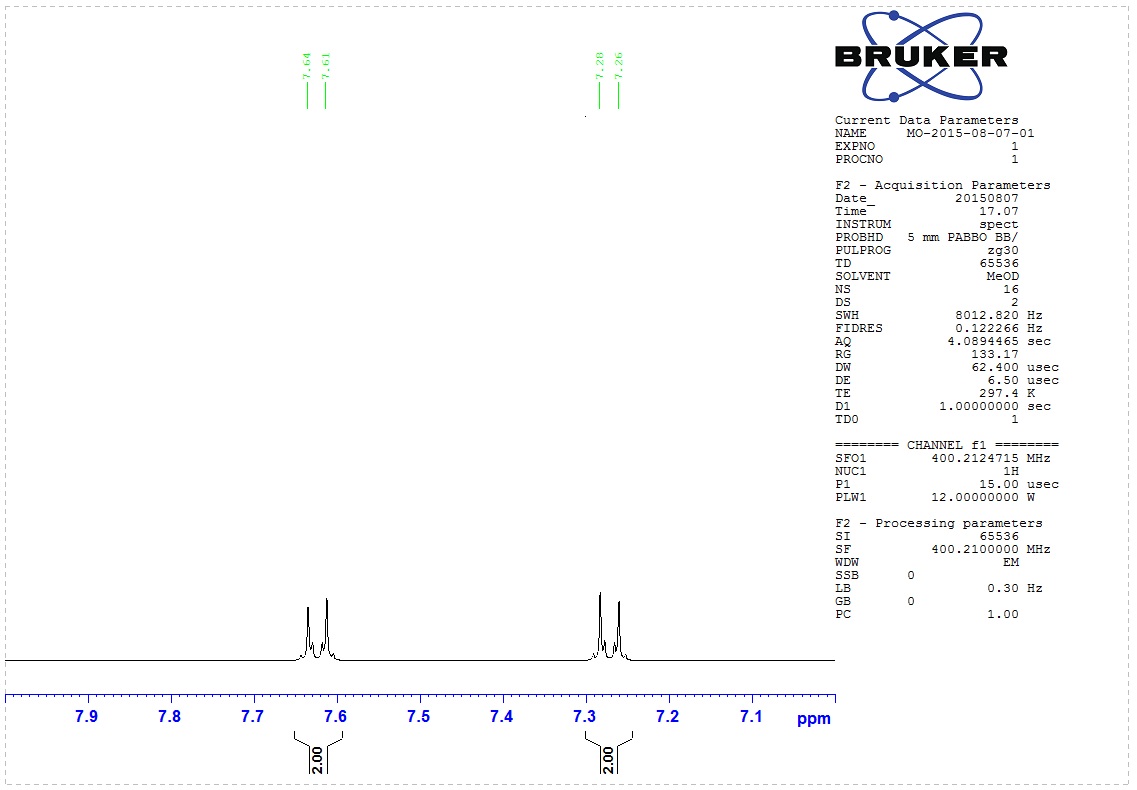


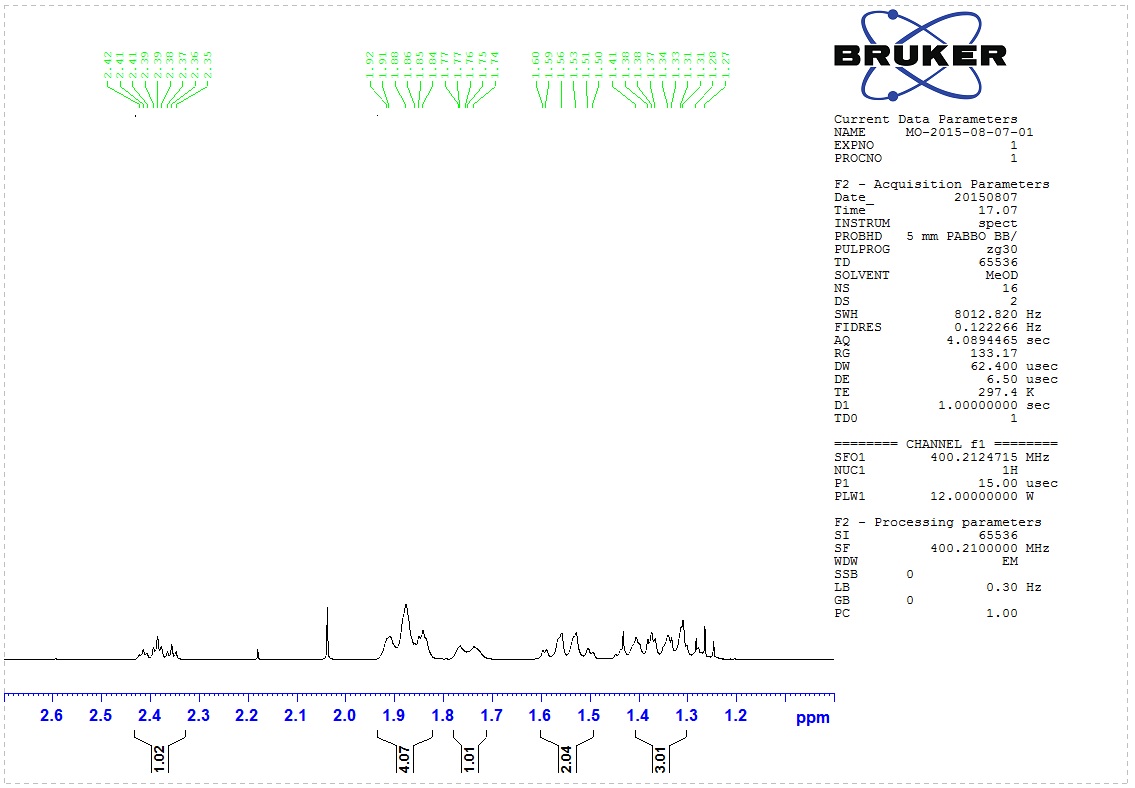


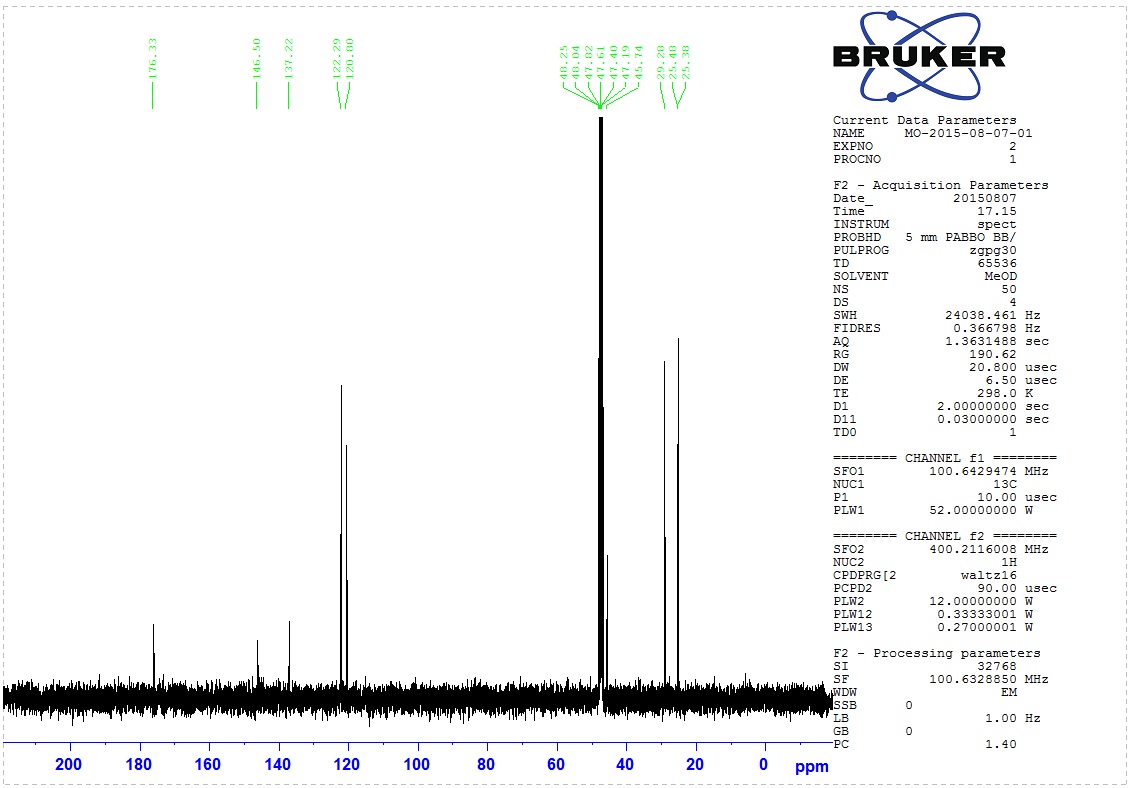


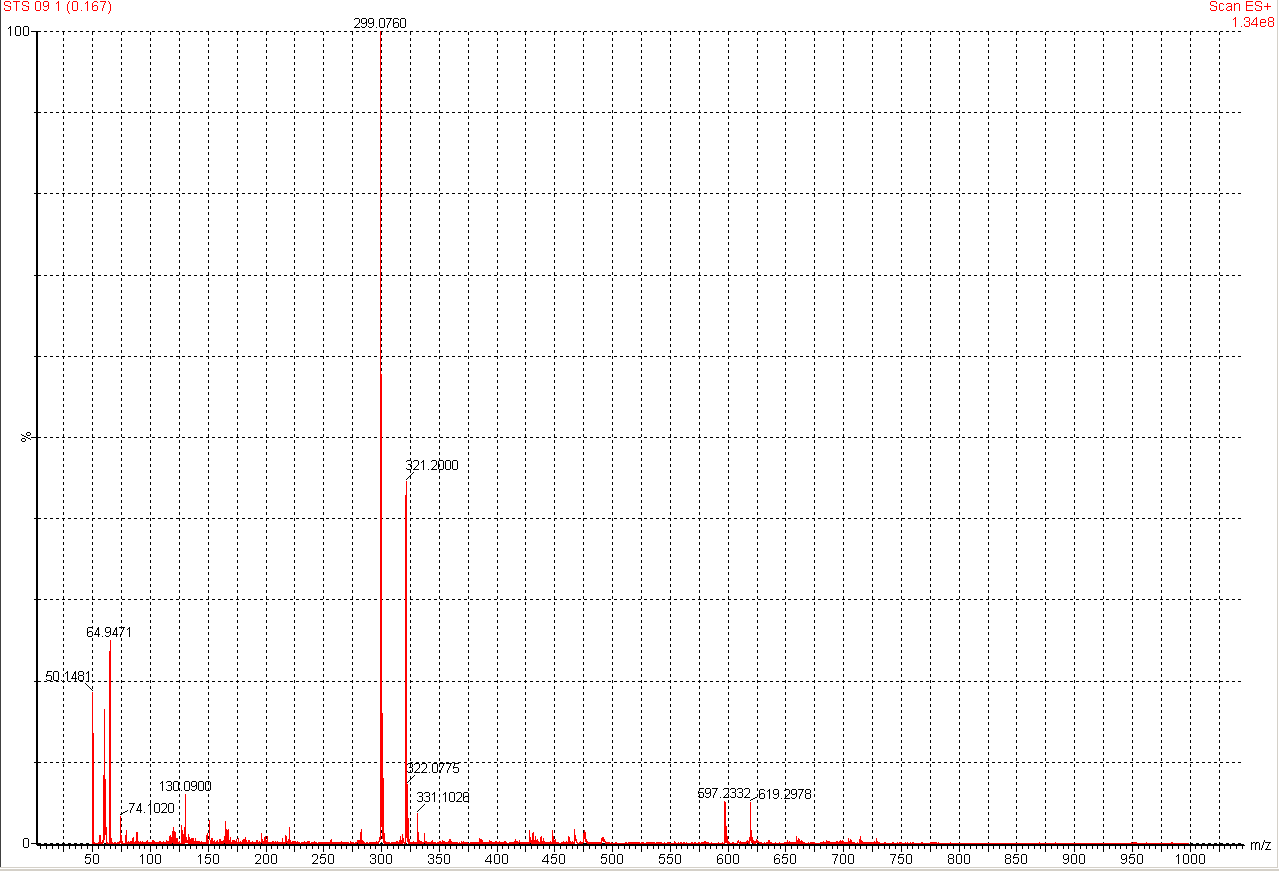


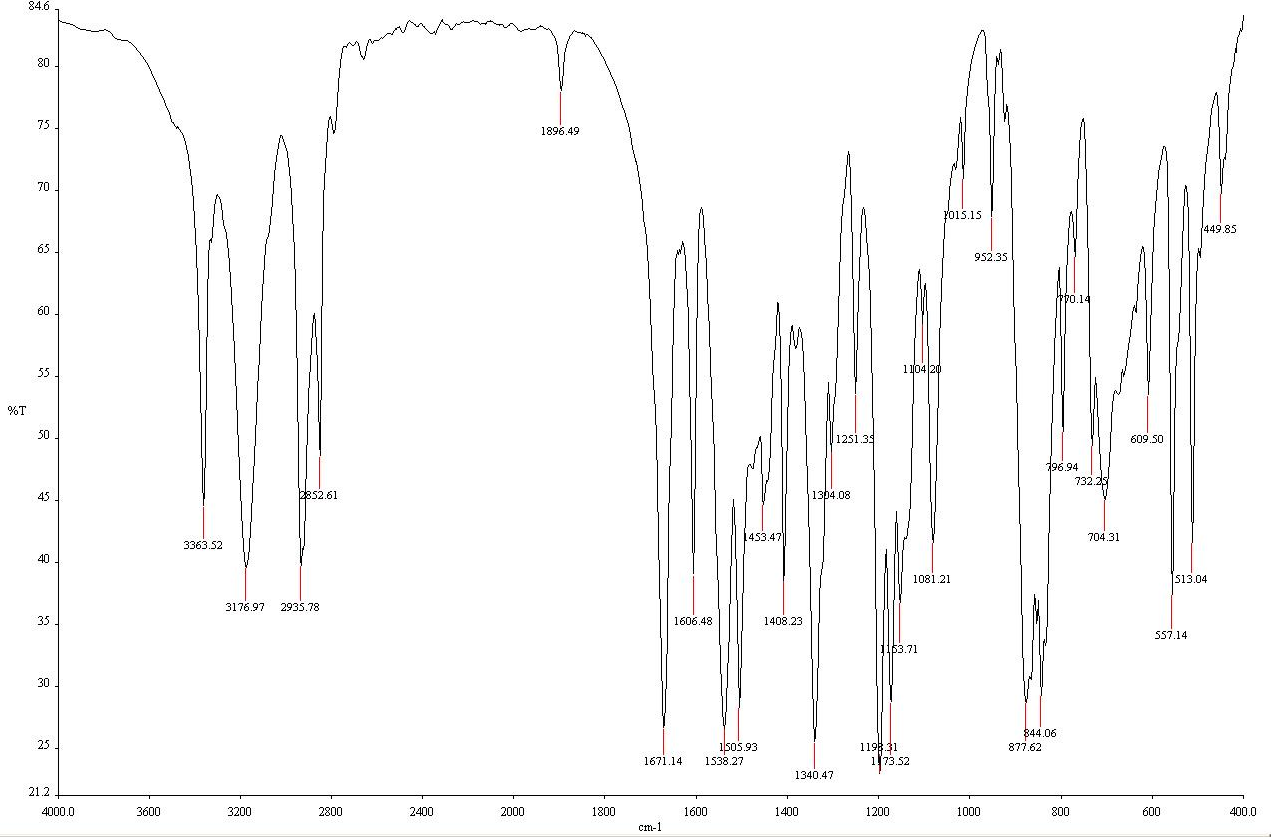


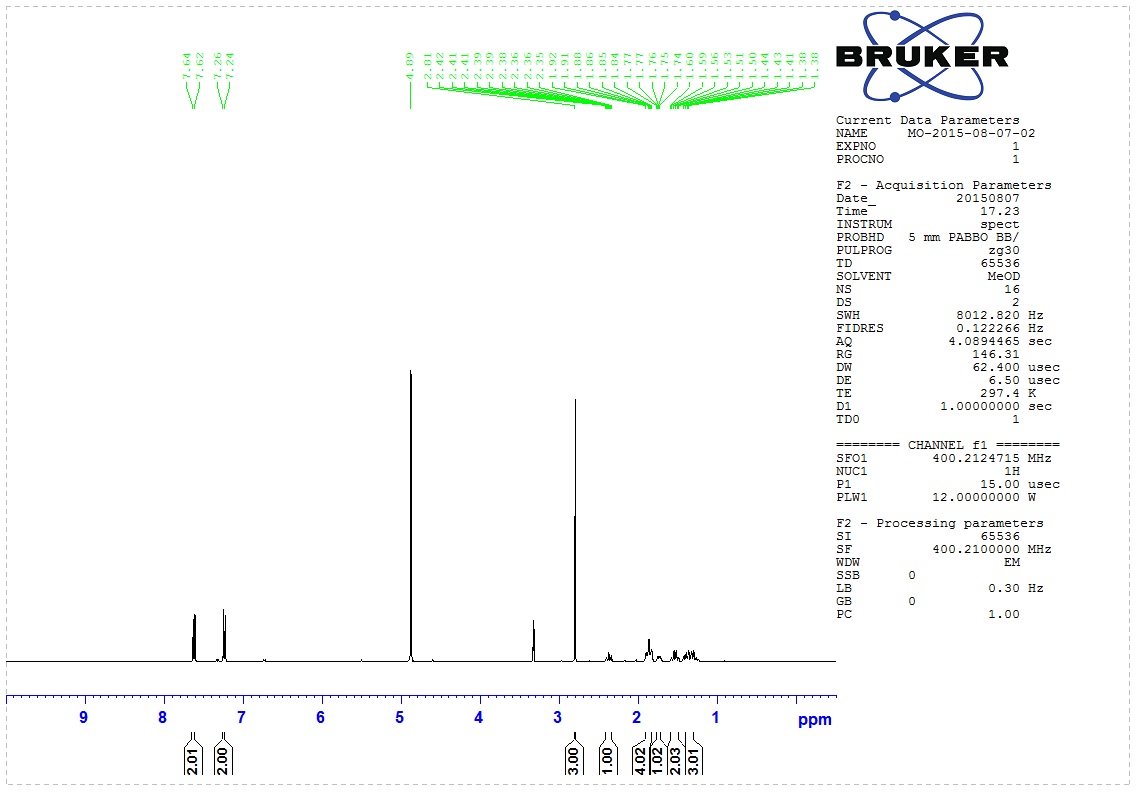


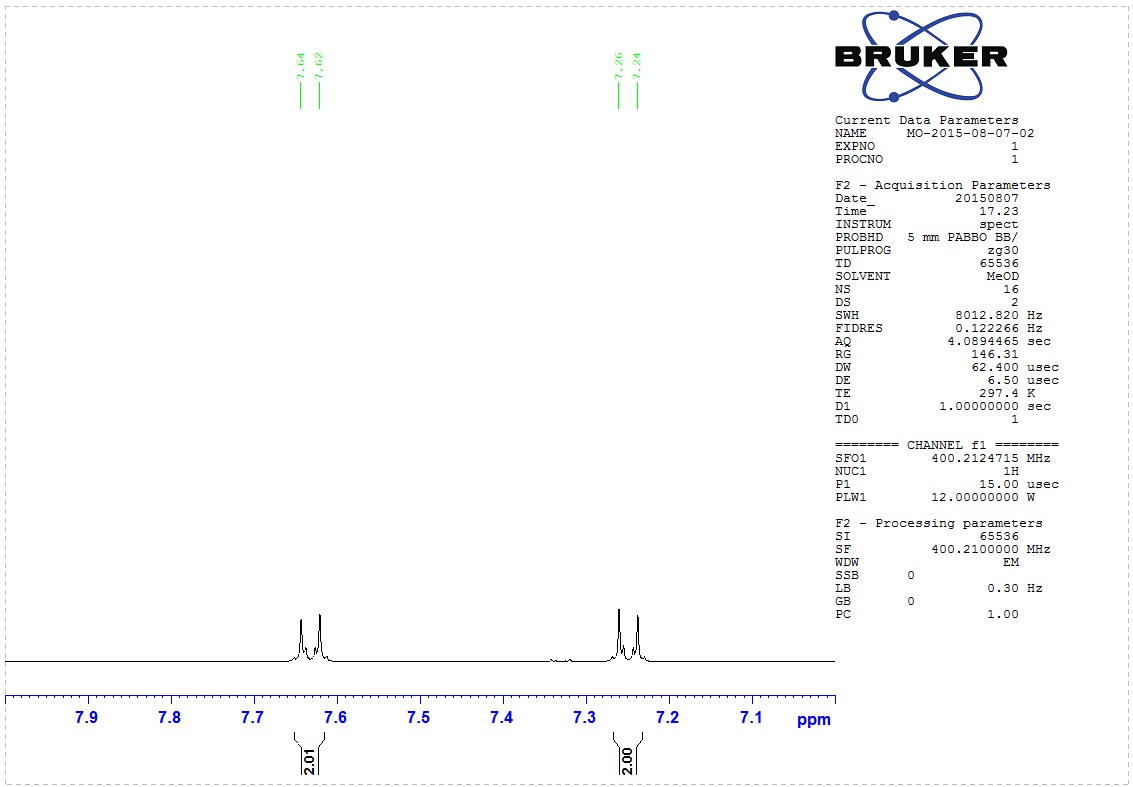


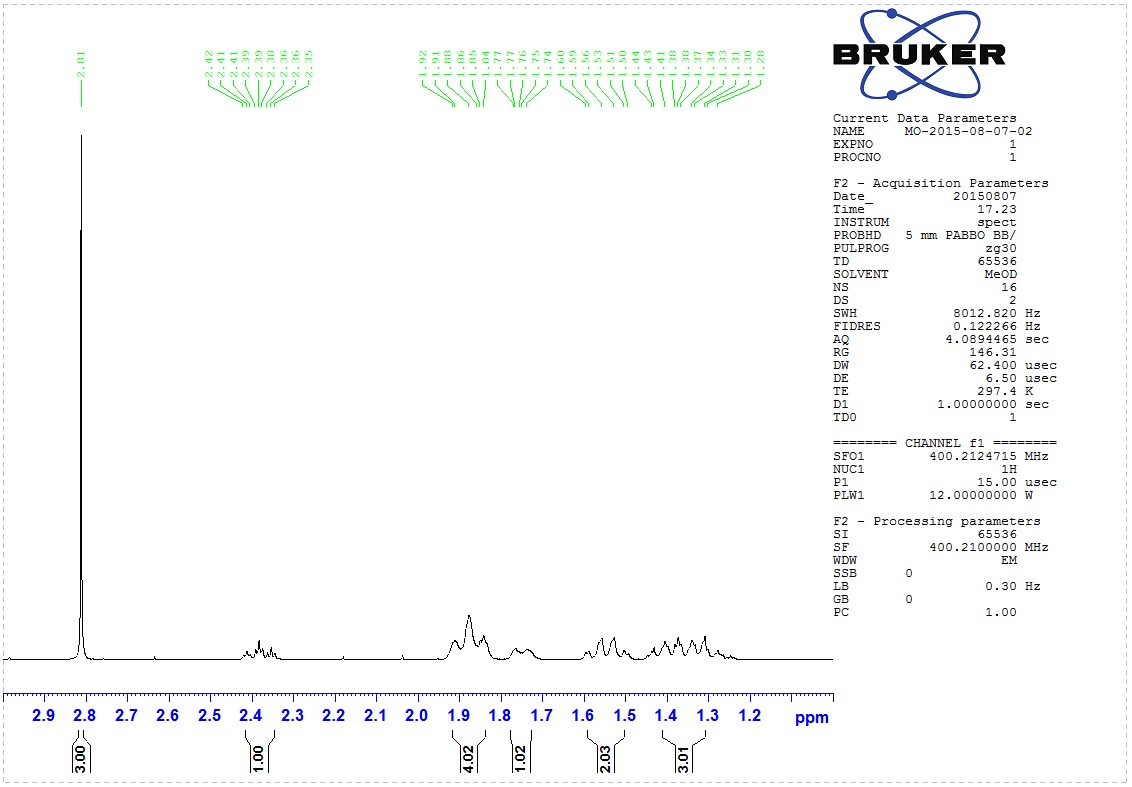


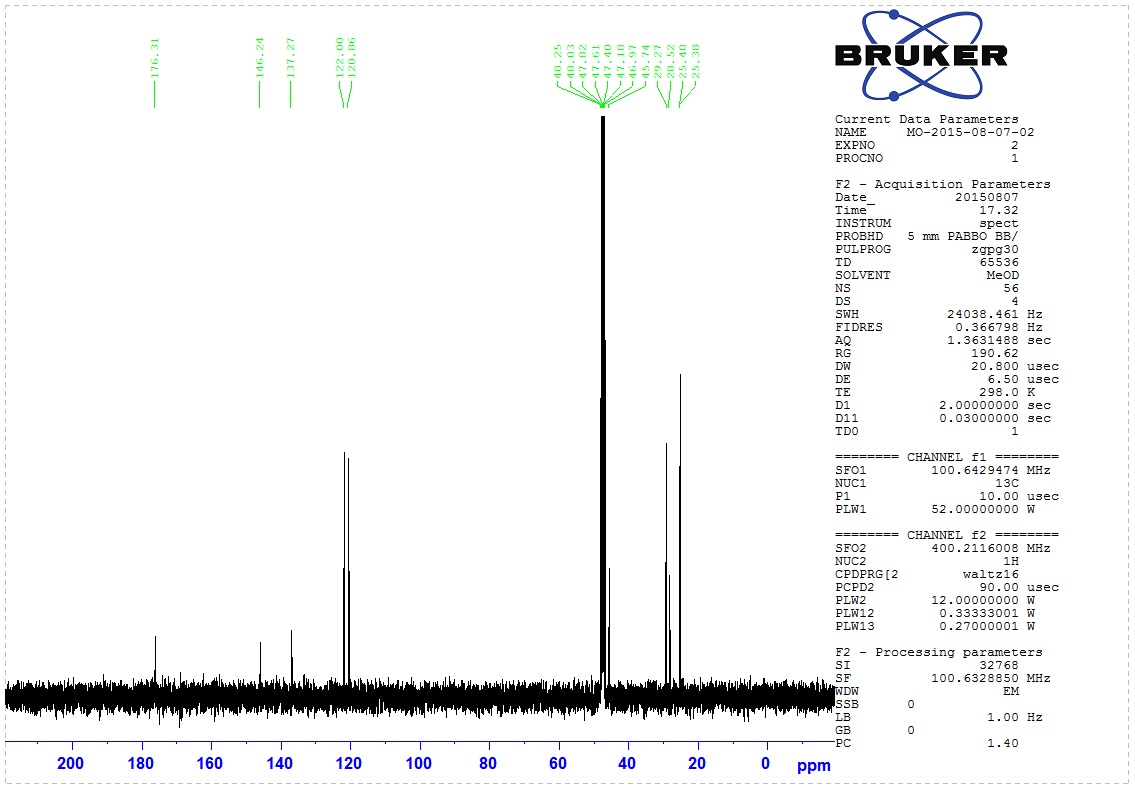


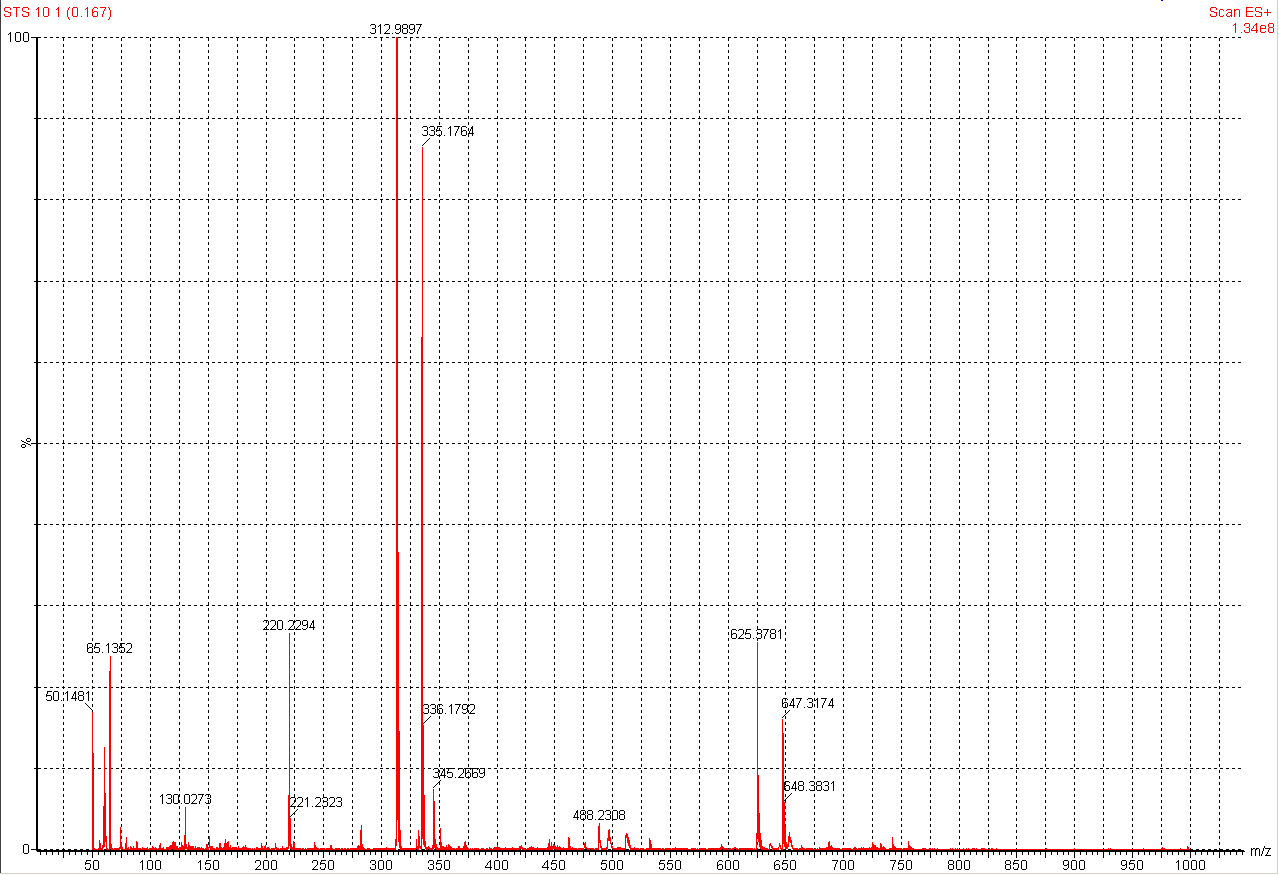


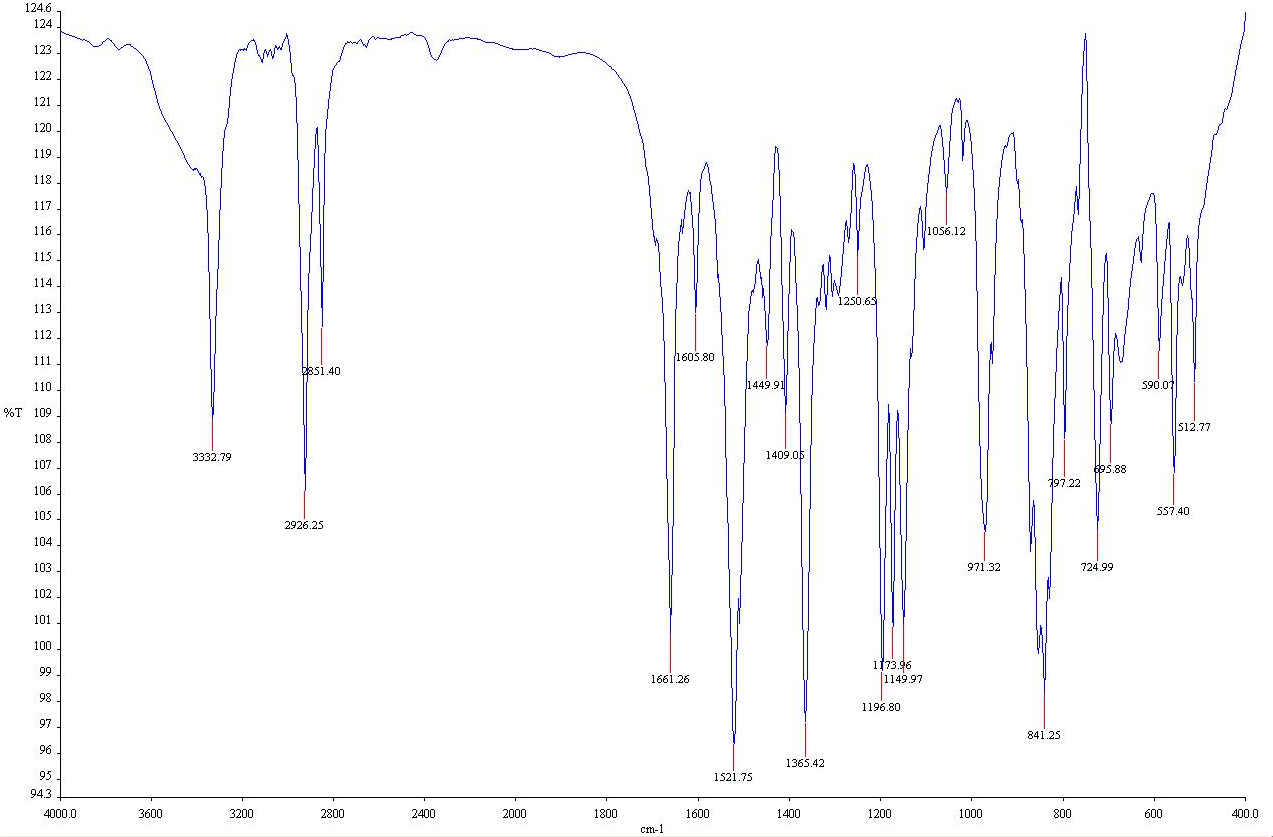


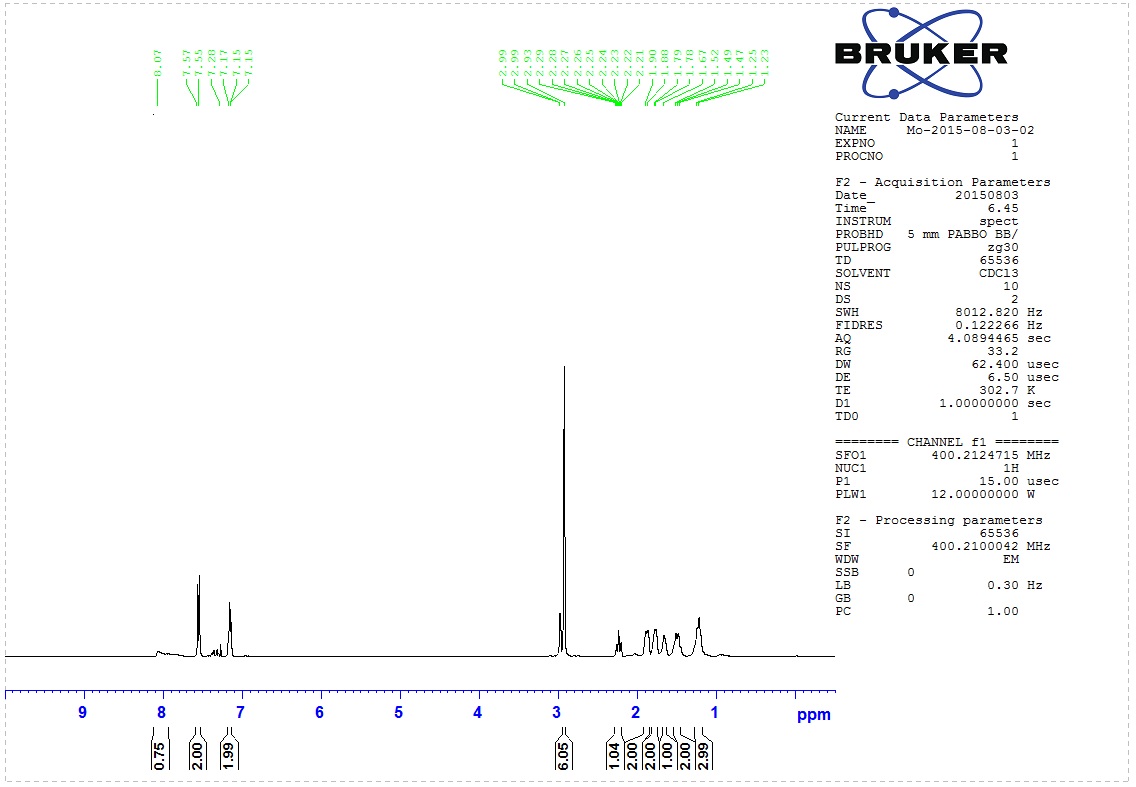


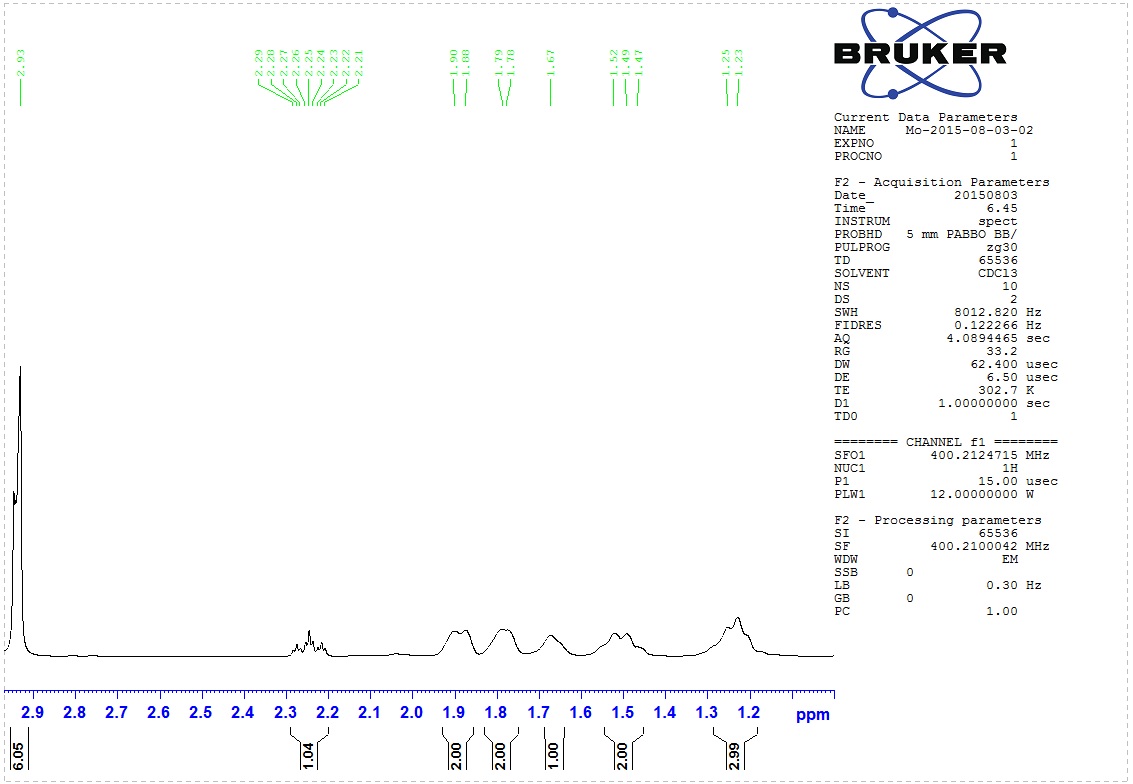


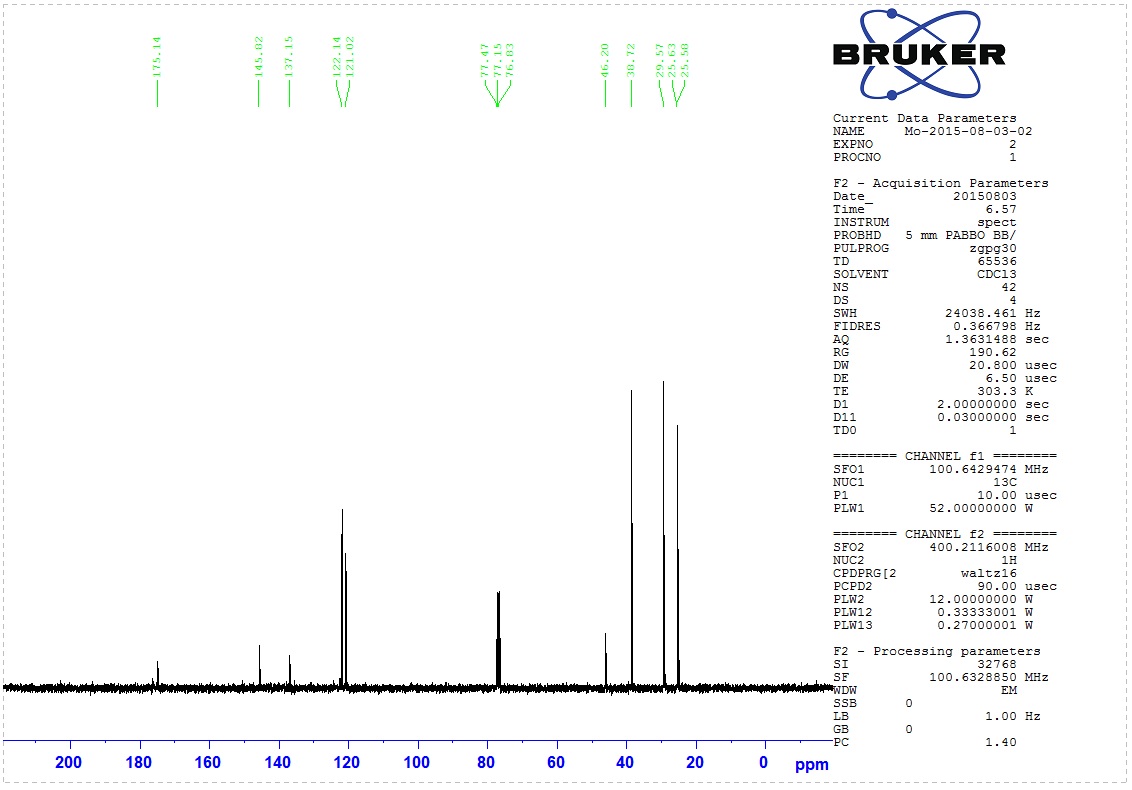


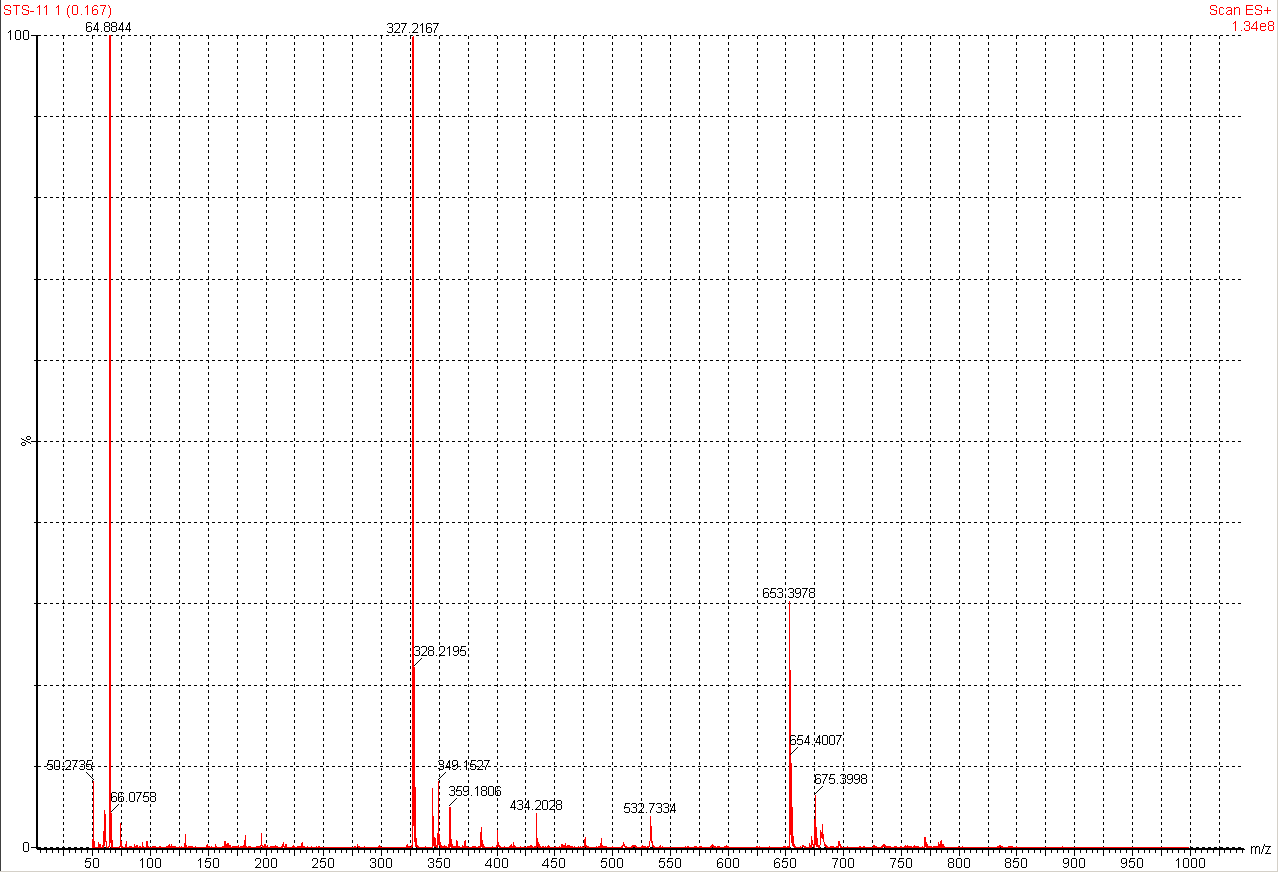


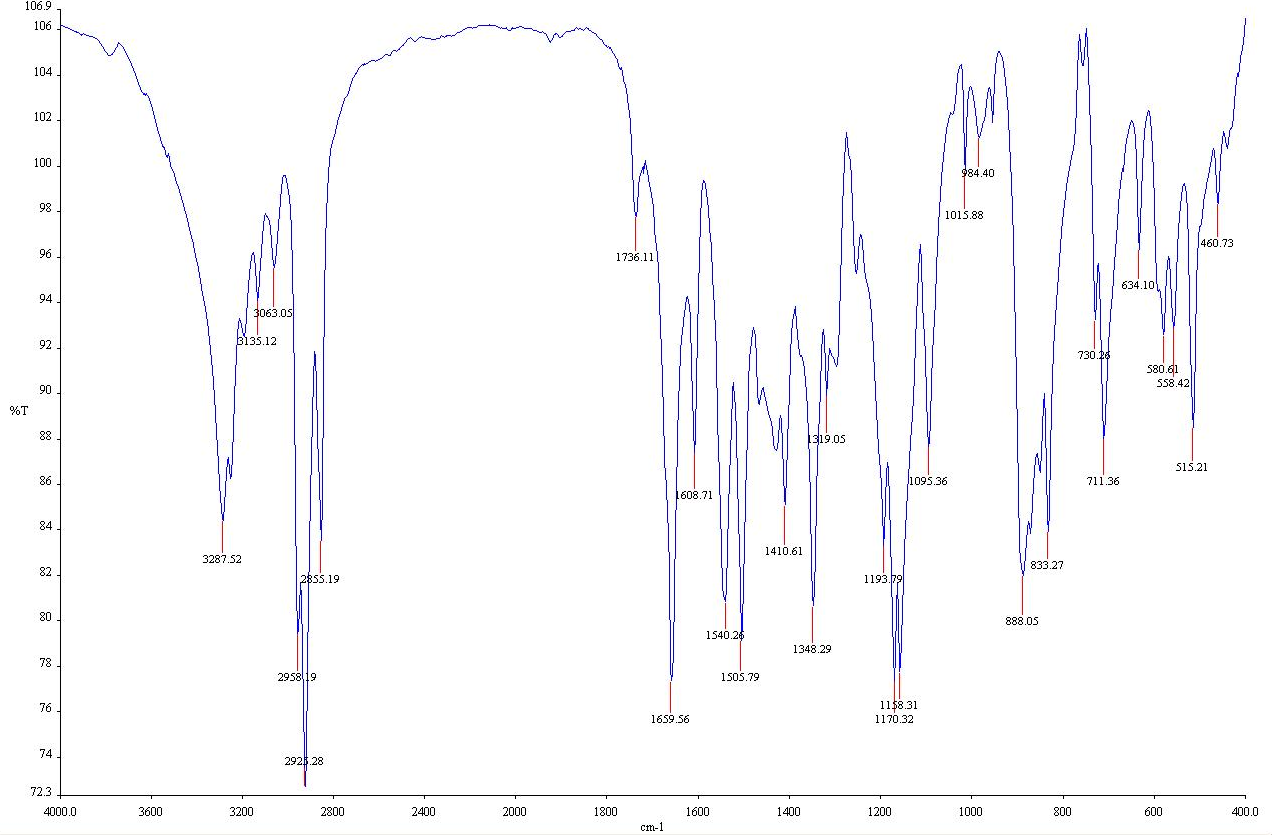


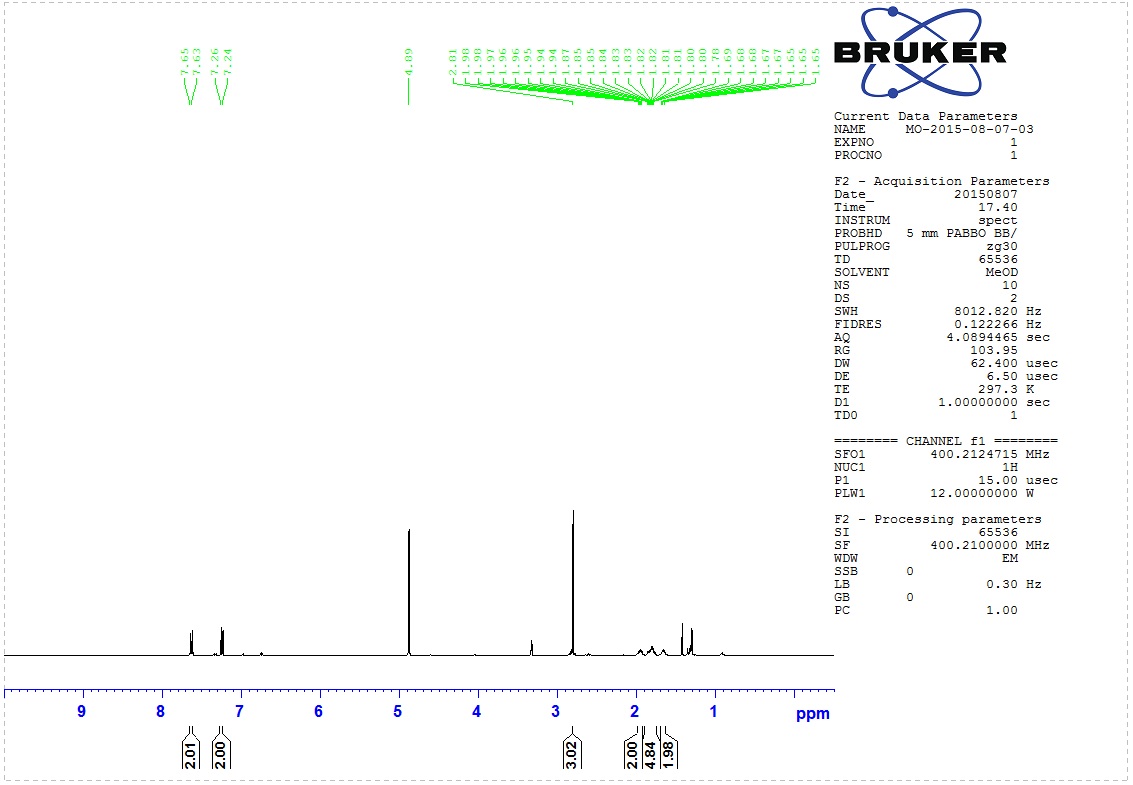


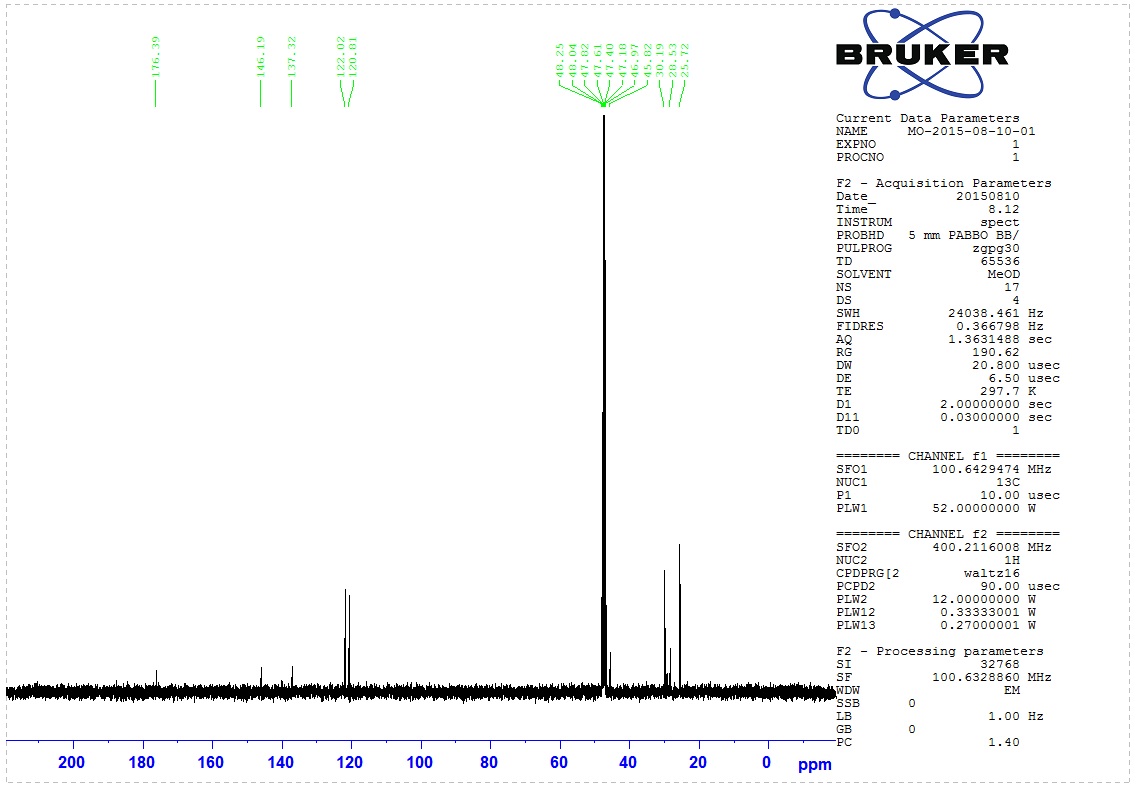


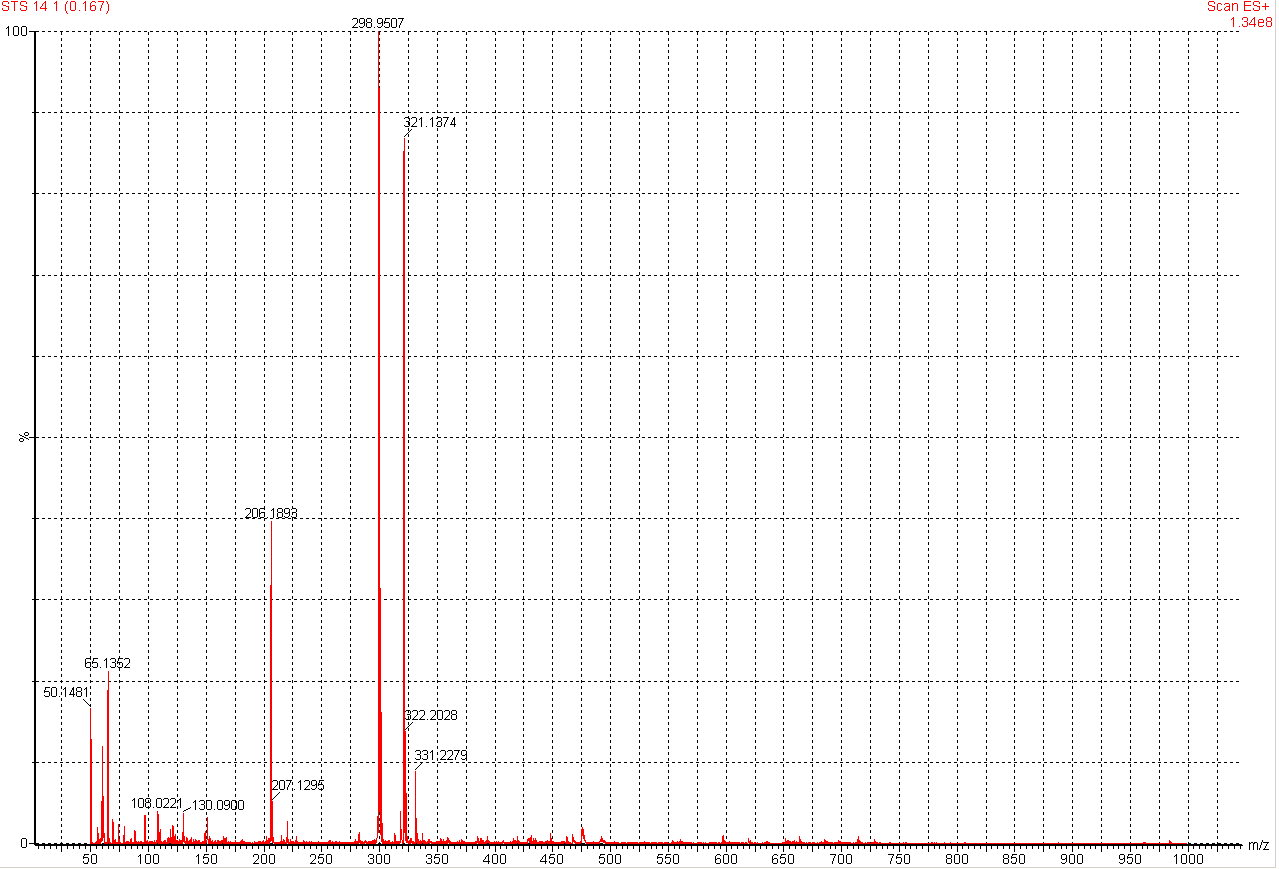

Supplement: Supplementary data — IR, 1H NMR, 13C NMR, and LC–MS charts of the final compounds. [file mmc1.doc]
